# The immunogenicity of pneumococcal conjugate vaccinations in patients with asplenia and hyposplenia: a systematic review

**DOI:** 10.1038/s41541-026-01514-9

**Published:** 2026-07-06

**Authors:** B. van Put, G. E. Loe-Sack-Sioe, L. G. Visser, S. P. Jochems, A. H. E. Roukens, H. Jolink

**Affiliations:** 1 Subdepartment of Infectious Diseases, Leiden University Center for Infectious Diseases (LUCID), Leiden University Medical Center, Leiden, The Netherlands; 2 Subdepartment of Research, Leiden University Center for Infectious Diseases (LUCID), Leiden University Medical Center, Leiden, The Netherlands

**Keywords:** Diseases, Immunology, Medical research, Microbiology

## Abstract

Pneumococcal conjugate vaccines (PCVs) are important preventive tools against invasive pneumococcal disease (IPD) in asplenic patients. This systematic review assesses current data and knowledge gaps on the immunogenicity of PCVs in asplenic patients. Twenty-seven studies were included assessing post-PCV IgG, opsonophagocytic functionality (OPA), or B-cell responses. We show that PCVs induce a strong serotype-specific IgG and OPA response in asplenic adults and children with various underlying conditions. Data in this review suggest that PPV23 has limited benefits for adults, but has been shown to broaden and boost the vaccine response in children with sickle cell disease. Moreover, we detected a lack of data on B-cell responses post-PCV and the optimal timing for vaccination around splenectomy. In conclusion, administration of PCVs in asplenic patients is supported by this review. The role of PPV23 in asplenic adults is probably limited, which supports the expected replacement of PPV23 by higher valent PCVs.

## Introduction

Individuals with impaired splenic function are at increased risk for severe bloodborne infection referred to as overwhelming post-splenectomy infection (OPSI). This complication is caused in particular by encapsulated bacteria and erythrocyte-invading parasites (such as *Plasmodium falciparum* and Babesia species)^1–3^. *Streptococcus pneumoniae* is the most common cause of OPSI (~90%) and leads to an increased rate of pneumonia and invasive pneumococcal disease (IPD) in asplenic patients^4,5^. OPSI has an estimated incidence rate ranging from ~0.13 in general to 1.8–3 in children per 100 person-years and is typically associated with mortality rates up to 50–70%^4,6–8^. The incidence rate peaks between the 1st and 5th year post-splenectomy, in certain age groups (<5 and >60 years of age), and in certain risk groups (especially when additionally immunocompromised), but remains permanently elevated throughout one’s lifetime^4,7^.

Various clinical conditions can lead to an absent (asplenia) or reduced (hyposplenia) splenic function. For example, splenectomy may be indicated to control post-traumatic bleeding or as a therapeutic intervention for hereditary spherocytosis, certain hemoglobinopathies (e.g., beta-thalassemia), and hematological malignancies (e.g. Hodgkin’s lymphoma). Also, splenic removal may be inevitable during surgery for several malignant and benign processes of the pancreas. Hyposplenia can be induced by underlying hematological, oncological, autoimmune, or infectious conditions^6^. Notably, the incidence and degree of hyposplenia in these populations have been scarcely studied, with the exception of sickle cell disease (SCD)^4^. In SCD, hyposplenia results from auto-splenectomy due to bouts of occlusive sickling of erythrocytes and subsequent splenic infarction^9^. Decline of splenic function in children with SCD is reported to commence before the age of 1 year and is complete at the age of 5 years^9^. Although some studies have shown persistence of splenic function until later in young-adult life^10^. The extensive heterogeneity among asplenia and hyposplenia-inducing conditions complicates the conduct of adequate research in asplenic patients, which leads to insufficient substantiation of the current standard of care. The current guidelines recommend preventive measures, which include vaccination (pneumococci, meningococci, and *Haemophilus influenzae* type b), antibiotic prophylaxis, and patient education. Although robust randomized controlled trials are lacking, observational studies show that implementation of these measures has led to a decrease in OPSI incidence^11^.

Most research in the context of asplenia and prevention of OPSI has been focused on *S. pneumoniae.* In general, the immunogenicity of polysaccharide antigens (either through natural infection or vaccination) is reduced in the absence of a functional spleen^12,13^. Specific splenic immunological features are important for eliciting an immune response against T-cell-independent antigens, such as pneumococcal capsular polysaccharides^4,5,12,13^. These include the surveillance and filtering of pathogens from the bloodstream and the production of natural IgM by splenic marginal zone memory B-cells^4,14^. The absence of these first-line defense mechanisms in asplenia results in an increased risk for uncontrolled and overwhelming spread of infections, as adaptive immunity requires days to develop and engage^4^. This immunological impairment combined with the capacity of the pneumococcal polysaccharide capsule to inhibit complement-mediated opsonization render the opsonophagocytic functionality of IgG antibodies particularly important for these patients^4,5,15^. Collectively, asplenic patients benefit from long-lasting protective antibodies with broad-serotype coverage, which rapidly opsonize invading pathogens and prevent dissemination. Consequently, prevention of infections caused by capsulated bacteria through vaccination is prioritized in the current management of this population^16^.

Until licensure of the 20-valent pneumococcal conjugate vaccine for adults in 2022 and for children in 2024, the internationally recommended vaccination schedule consisted of the highest available polyvalent pneumococcal conjugate vaccine (PCV) followed by the 23-valent pneumococcal polysaccharide vaccine (PPV23)^4,6^. In this way, the PCV-specific benefits, such as induction of immunological memory, are combined with protection against the broadest possible range of disease-inducing serotypes. However, PPV23 induces a T-cell independent (TI) B-cell response, which is characterized by a lack of long-term protection as no immunological memory, affinity maturation, and class switching of memory B-cells is induced^17^. To elicit a more sustained T-cell-dependent (TD) memory response, polysaccharides have been conjugated to carrier proteins in PCVs. Hereby, parts of the carrier protein will be presented in the context of MHC-II to T-cells, which provide help for B-cells to produce anti-polysaccharide antibodies and enter the germinal center reaction (GCR)^17^.

PCVs have been shown to induce higher post-immunization IgG binding antibody titers and improved pneumococcal opsonophagocytic activity in asplenic patients (adults and children) when compared to PPV23^18–21^. It is currently unknown whether the antibody response in asplenic and hyposplenic patients to PCV is inferior compared to that of healthy individuals. Similarly, the optimal timing for vaccination around splenectomy and the need and benefits of PCV booster doses are unclear.

The objective of this systematic review is to summarize the current data on the immunogenicity of PCVs in asplenic patients, to generate a comprehensive and practical overview of existing knowledge, and to identify knowledge gaps for future research in this vulnerable population.

## Results

**Fig. 1:**
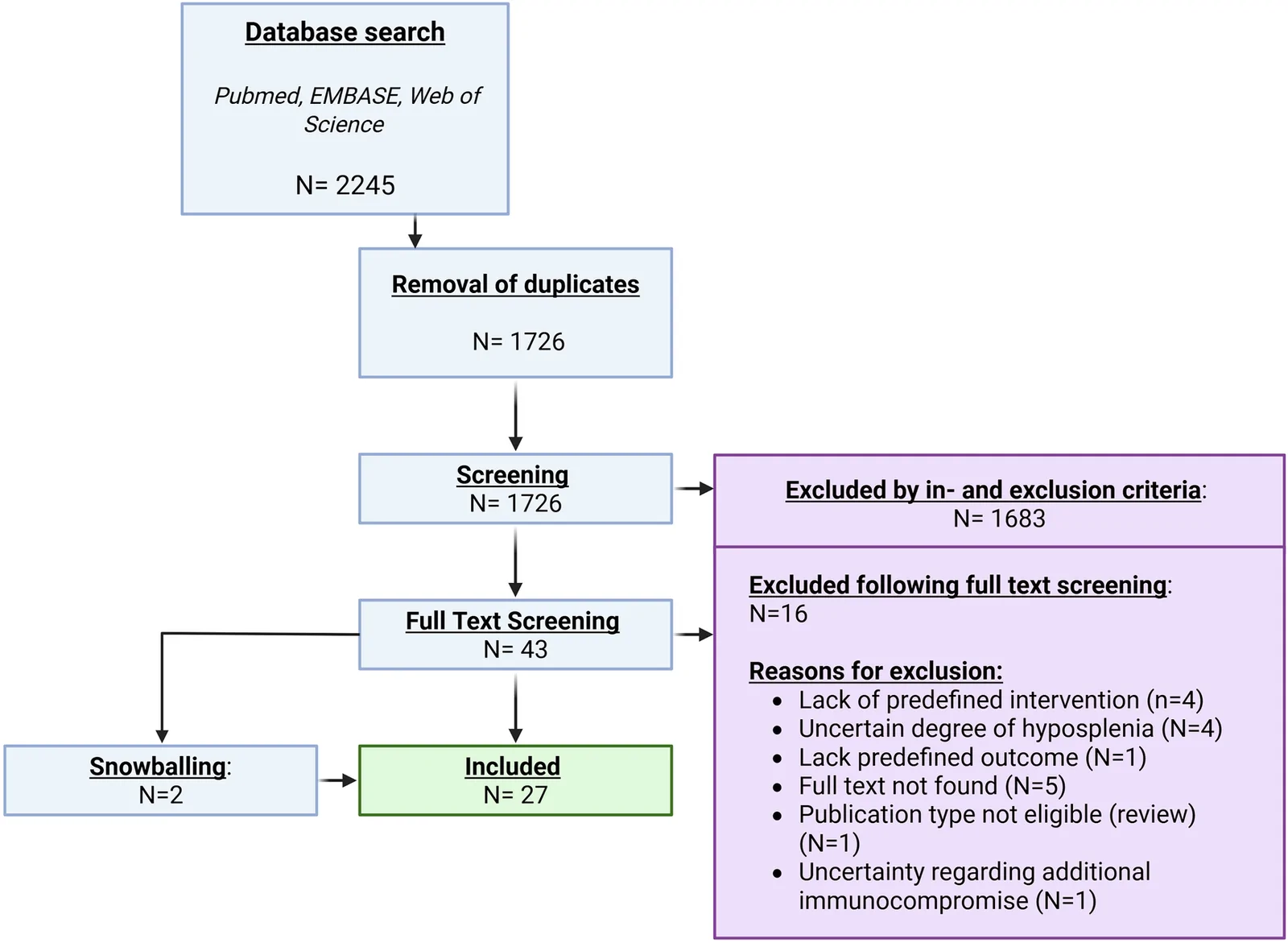
Flowchart of the screening and inclusion process. The number of articles per step is indicated by N.

Our search resulted in 2245 hits with 519 duplicates. 1726 articles remained available for screening of title and abstract, of which 43 articles fulfilled the general inclusion and exclusion criteria. For five titles, the content was untraceable, resulting in a total of 38 articles eligible for full-text screening, after which 25 articles were included. The reference lists of these articles were screened, and two additional articles that met eligibility criteria were added. In the end, 27 articles were included in this systematic review (Fig. 1).

### Studies

The included articles consisted of eight randomized controlled trials, 16 prospective nonrandomized clinical trials, and three observational studies assessing longevity. In total, 750 asplenic individuals (mean trial population size = 49 with an SD of 37.5) and 942 individuals with sickle cell disease (mean trial population size = 86 with an SD of 64.4) were assessed in the included studies. For studies with heterogeneous study populations, the underlying diseases are listed in the legend of Table S1 (Supplementary Materials 2). Nine trials included a total of 371 controls, which consisted of healthy individuals or not-splenectomized but otherwise comparable patients (e.g., patients with B-thalassemia but no splenectomy). Twelve trials studied adults or mixed-aged populations, and 15 trials studied a pediatric population. The PCVs that were assessed comprise PCV7 (*n* = 11), PCV10 (*n* = 2), PCV13 (*n* = 10), and PCV15 (*n* = 1). The vaccination schedules comprised: a single PCV dose (*n* = 8), a PCV followed by PPV23 (*n* = 5), two sequential doses of PCV (*n* = 3), and extended multiple-dose schedules followed by PPV23 (*n* = 8). Seven pediatric trials assessed a multiple-dose childhood vaccination schedule starting at 2 months up to 2 years of age. The RCTs compared PCV with PPV23 (*n* = 5), two different PCVs (*n* = 1), different extended schedules (*n* = 1), and the effect of zinc supplementation versus placebo (*n* = 1). The characteristics of the studies are given in Table 1 and extensive information can be found in the Supplementary Materials (Tables S1 and S2).

**Table 1:** Risk of bias assessment: randomized controlled trials using Rob2

Study	Domain 1	Domain S cross-over RCTs	Domain 2^a^	Domain 3	Domain 4	Domain 5	Final assessment
Vernacchio^19^	SC		L	L	SC	SC	Some concerns
Vernacchio^20^	SC		L	L	SC	SC	Some concerns
Smets^29^	L		L	L	SC	SC	Some concerns
Orthopoulos^28^	SC		L	L	SC	SC	Some concerns
Rezai^41^ ^b^	SC	H	L	SC	L	SC	High risk
Sari^59^	SC		L	L	SC	SC	Some concerns
Melica^21^	L		SC	SC	L	SC	Some concerns
Quin^40^	L		L	L	L	L	Low risk

Domain 1: The randomization process, Domain 2: Deviations from intended interventions, Domain 3: Missing outcome data, Domain 4: Measurement of outcome, Domain 5: Selection of reported results. Domains are scored with low risk (l), some concerns (sc), or high risk (H). The Final assessment is scored with low risk of bias, some concerns, or high risk of bias.

^a^We choose to assess the risk of bias of the ‘intention-to-treat’ effect (Domain: assignment to intervention).

^b^For this trial, the Rob2 tool for individually randomized cross-over (or other matched) trials was used.

### Patients

The adults in the included studies assessing the primary response were asplenic due to sickle cell disease or had received splenectomy due to trauma, solid organ malignancy, hematological malignancy (not active during vaccination), benign processes requiring resection, beta-thalassemia, or hereditary spherocytosis. The children in the included studies were asplenic due to sickle cell disease, congenital asplenia, or had received splenectomy due to hereditary spherocytosis, trauma, or benign processes requiring resection.

### Risk of bias: randomized controlled trials

In most trials, we classified the risk of bias as ‘Some concerns,’ in one trial as ‘Low risk,’ and in one trial as ‘High risk’ (Table 1). The full assessment can be found in Supplementary Materials 2. The most frequent source of bias occurred in the randomization process, which was often incompletely described, in the absence of blinding, and the absence of an available protocol or statistical plan.

### Risk of bias: nonrandomized clinical trials

The risk assessment of bias in nonrandomized trials is displayed in Table 2. The scoring system is described per domain in Supplementary Materials 1. Most trials were categorized as ‘FAIR,’ and the risk of bias was mostly the result of small sample sizes, heterogeneity in the study population, absence of a control group, and the lack or inability to perform subgroup analysis and adjustment for confounders.

**Table 2:** Assessment of the risk of bias of nonrandomized studies using the Newcastle Ottawa Scale for cohort studies

Study	Selection				Comparability	Outcome			
	Representativeness exposed cohorts	Selection of non-exposed cohort	Ascertainment of exposure	Outcome not present at start study	Comparability of cohorts through design or analysis	Assessment outcome	Follow-up duration sufficient	Adequacy of follow-up	Final^a^
Nowak^31^	0	1	1	1	1	1	1	0	GOOD
O’Brien^30^	1	1	1	1	1	1	1	0	GOOD
Stoehr^36^	1	0	1	1	0	1	1	1	GOOD
Reinert^34^	1	0	1	1	0	1	1	0	FAIR
Mikoluc^42^	0	0	1	1	1	1	0	1	FAIR
Stanford^23^	0	0	1	1	1	1	1	1	GOOD
Meerveld^27^	0	0	1	1	1	1	1	0	FAIR
Rosado^13^	0	1	1	0	1	1	0	1	FAIR
Rivera^35^	0	0	1	1	0	1	0	1	FAIR
Papadatou^25^	1	0	1	1	1	1	1	1	GOOD
Papadatou^26^	0	0	1	1	1	1	1	1	GOOD
De Montalembert^37^	1	0	1	1	1	1	1	0	GOOD
Nived^24^	0	0	1	1	0	1	1	0	FAIR
Disu^32^	1	1	1	1	1	1	0	1	GOOD
Sirima^33^	1	1	1	1	1	1	1	1	GOOD
Gazi^60^	0	1	1	1	1	1	0	1	GOOD
Szenborn^39^	0	0	1	1	1	1	1	0	FAIR
Papadatou^38^	1	0	1	1	1	1	1	1	GOOD
Takeshita^18^	0	0	1	0	0	1	0	1	FAIR

^a^GOOD: 6–9 starts, FAIR: 3–5 starts, POOR: 0–2 points.

### Serological response

The outcomes and conclusions of the included trials are displayed in Table 3. In total, 22 studies assessed serotype-specific IgG binding responses, and four studies assessed the general anti-pneumococcal IgG binding response without distinguishing between serotypes. The performed assays and definitions of an adequate response or threshold of protection applied in the trial were variable, as can be observed in Tables S1 and S2 (Supplementary Materials).

**Table 3:** Summary of included studies (study characteristics and primary outcomes)

Pediatric studies assessing the IgG and/or OPA response after pneumococcal conjugate vaccination								
Study		Design	Patients	*N*	Vaccines (nr doses)	Groups	Outcomes	
							PS- or pneumococcal-specific IgG response (↑/sig↑/No↑)*	OPA Response (↑/sig↑/No↑)*
Vernacchio et al.^19^		RCT	>2 yrs	23	PCV7 (2) + PPV23 (1) vs PPV23 (1)	Group PCV7 + PPV23	1st PCV7: sig↑ 6/7 PS 2nd PCV7: sig↑ 2/7 PS PPV23: sig↑ 2/7 PS	NA
						Group PPV23	sig↑ 7/7 PS	NA
Vernacchio et al.^20^		RCT	>2 yrs	23	PCV7 (2) + PPV23 (1) vs PPV23 (1)	Group PCV7 + PPV23	NA	↑ 7/7 PS
						Group PPV23	NA	↑ 7/7 PS
Smets et al.^29^		RCT	<5 yrs	21	PCV7 (1) vs PPV23 (1)	Group PCV7	Sig↑ 2/7 PS	NA
						Group PPV23	No ↑	NA
Quinn et al.^40^		RCT	2–17 yrs	103	PCV13 (1) vs PCV15 (1)	Group PCV13	↑13/15 PS	↑13/15 PS
						Group PCV15	↑15/15 PS	↑15/15 PS
Nowak-Wegrzyn. et al.^31^		CS	2–12 m	38	PCV7 (3) + PPV23 (1) or PCV7 (1) + PPV23 (1)	Group 3xPCV7 + PPV23	NA	3rd PCV7: sig↑ 2/2 PS PPV23: sig↑ 2/2 PS
						Group 1xPCV7 + PPV23	NA	PCV7: sig↑ 2/2 PS PPV23: sig↑ 2/2 PS
O’Brien et al.^30^		CS	2–12 m	47	PCV7 (3) + PPV23 (1) or PCV7 (1) + PPV23 (1)	Group 3xPCV7 + PPV23	3rd PCV7: sig↑ 7/7 PS PPV23: sig↑ 7/7 PS	NA
						Group 1xPCV7 + PPV23	PCV7: sig↑ 7/7 PS PPV23: sig↑ 7/7 PS	NA
Stoehr et al.^36^		CS	2–18 yrs	39	PCV7 (1) + PPV23 (1)	-	PCV7: sig↑ 7/7 PS PPV23: sig↑ 2/7 PS	NA
Reinert et al.^34^		CS	all 2 m	51	PCV7 (3) + PPV23 (1)		3rd PCV7: ↑ 7/7 PS PPV23: ↑ 7/7 PS	3rd PCV7: ↑ 4/4 PS
Mikoluc et al.^42^		CS	4–19 yrs	30	PCV7 (1)	-	Sig↑ 7/7 PS	NA
Rivera-Olivero et al.^35^		CS	2–59 m	22	PCV7 (3) or PCV7 (2) or PCV7 (1)	-	All schedules: ↑ 7/7 PS	NA
De Montalembert et al.^37^		CS	6–17 yrs	158	PCV13 (2)	-	1st PCV13: sig↑ 13/13 PS 2nd PCV13: sig↑ 13/13 but lower than >1st	1st PCV13: sig↑ 13/13 PS 2nd PCV13: sig↑ and higher than 1st for 4 PS
Disu et al.^32^		CS	5 m–5 yrs	151	PCV13 (1)	-	sig↑ anti-pneumococcal IgG	NA
Szenborn et al.^39^		CS	2–17 yrs	18	PCV10 (1)	-	↑ 10/10 PS	↑ 7/10 PS
Sirima et al.^33^		CS	2–23 m	150	PCV10 (4) or PCV10 (3) or PCV10 (2)	Group 4x PCV10	3rd PCV10: ↑10/10 PS Booster: ↑10/10 PS	3rd PCV10: ↑10/10 PS Booster: ↑10/10 PS
						Group 3x PCV10	2nd PCV10: ↑10/10 PS Booster: ↑10/10 PS	2nd PCV10: ↑10/10 PS Booster: ↑10/10 PS
						Group 2x PCV10	1st PCV10: ↑10/10 PS Booster: ↑10/10 PS	1st PCV10: ↑10/10 PS Booster: ↑10/10 PS
**Adult studies assessing the IgG and/or OPA response after pneumococcal conjugate vaccination**								
**Study**		**Design**	**Patients**	***N***	**Vaccines (nr doses)**	**Groups**	**Outcomes**	
							**PS- or pneumococcal-specific IgG response** **(↑/ sig ↑/ No ↑)**	**OPA** **Response** **(↑/ sig ↑/ No ↑)**
Orthopoulos et al.^28^		RCT	12–41 yrs	35	PCV7 (1) + PPV23 (2) vs PCV7 (2) + PPV23 (1)	-	Both groups 1st PCV7: Sig↑ 2nd PCV/PPV23: no ↑ Booster PPV23: Sig↑ 2PS* in group 2 *shared PS	NA
Rezai et al.^41^		RCT	20–44 yrs	47	PCV13 (1) + PPV23 (1) vs PPV23 (1) + PCV13 (1)	-	Both groups After 2nd dose: Sig↑ anti-pneumococcal IgG	NA
Sari et al.^59^		RCT	12–38 yrs	56	PCV13 (1) + zinc vs placebo	-	Sig↑ anti-pneumococcal IgG	NA
Melica et al.^21^		RCT	≥18 yrs	128	PCV13 (1) + PPV23 (1) vs PPV23 (1)	Group PCV13 + PPV23	↑13/13 PS	NA
						Group PPV23	↑13/13 PS (<group 1)	
Stanford et al.^23^		CS	18–81 yrs	111	PCV7 (1) + PPV23 (1)	-	PCV7: ↑ 7/7 PS PPV23 (*n* = 33): No ↑	NA
Meerveld-Eggink et al.^27^		CS	≥18 yrs	54	PCV7 (2) + PPV23 (1)	-	1st PCV7: sig ↑ 7/7 PS 2nd PCV7: sig ↑ 1/7 PS PPV23: No ↑	NA
Nived et al.^24^		CS	≥18 yrs	24	PCV13 (1)	-	sig ↑ : 9/12 PS + ↑: 2/11 PS	NA
Papadatou et al.^25^		CS	19–48 yrs	39	PCV13 (1)	-	Sig ↑ : 5/5 PS	NA
Papadatou et al.^26^		CS	≥18 yrs	24	PCV13 (1)	-	Sig ↑ : 3/3 PS	NA
**Studies assessing the B-cell response following vaccination with pneumococcal conjugate vaccines in asplenic adults and children**								
**Study**		**Design**	**Patients**	***N***	**Vaccines (nr doses)**	**Outcomes**		
Rosado et al.^13^		CSS	1–18 yrs 18–82 yrs	78	History of PCV7 and/or PPV23	Assay: ELISPOT History of PPV23: proportion of IgM and switched pneumococcal-specific memory B-cells and secretion significantly reduced compared to HCs History of PCV7 prior to splenectomy or PPV23: anti-pneumococcal IgG memory B-cells reduced compared to HCs. History of PCV7 post-splenectomy: anti-pneumococcal IgG memory B-cells similar to HCs.		
Papadatou et al.^25^		CS	19–48 yrs	24	PCV13 (1)	Assay: ELISPOT Pre-vaccination: all had memory B-cells for at least 1/5 PS Post-vaccination day 28: sig↑ in memory B-cells for 5/5 PS		
Gazi et al.^60^		CS	20–53 yrs	14	PCV13 (1) + PPV23 (1)	Assay: ELISPOT Pre-vaccination: PCV13-specific memory B-cells were significantly lower than controls Post PCV13: similar increase in patients and controls Post-PPV23: no increase in both groups		

*Summary of study outcomes (increase, statistically significant increase, or no increase) of PS-specific or total pneumococcal-specific IgG and/or OPA titers compared to baseline. The proportion of serotypes with an increase (number with increase/total number serotypes tested) is displayed… untill… (no increase in titer).

*RCT* randomized controlled trial, *CS* cohort study, *CSS* cross-sectional study, *N* size of patient study population, *nr* number, *PS* pneumococcal serotype, *sig* statistically significant, *OPA* opsonophagocytic activity, *PPV23* 23-valent pneumococcal polysaccharide vaccine, *vs* versus, *yrs* years, *m* months, *PCV* pneumococcal conjugate vaccine, *NA* not applicable, *SCD* sickle cell disease, *HCs* healthy controls, *PCs* patient controls without splenectomy, *GMC* geometric mean concentration.

### Primary response in asplenic adults

Although most study populations were considered naive for the investigated PCV, it remains difficult to determine to what extent the measured antibody response reflects a true first encounter of the immune system with the antigen, as previous pneumococcal carriage or receipt of lower-valent PCVs can influence baseline immunity. As a result, PS-specific IgG levels measured in adults (and older children) prior to PCV vaccination were often already detectable, as can be observed in Tables S2 and S3, Supplementary Materials 2. After primary vaccination with PCV7 or PCV13, a consistent and statistically significant increase in PS-specific IgG geometric mean concentrations (GMCs) for most tested serotypes was observed in all studies. Peak response was measured 3–12 weeks after vaccination. Some trials used a threshold of protection to report their outcomes. These trials reported that 94–100% of participants exceeded the WHO threshold of ≥0.35 µg/mL^22^ and 46–100% had IgG GMCs ≥1.00 µg/mL after one dose of PCV7 or PCV13^23–27^. The fold increase was used as outcome measure in three trials, which found that higher baseline GMCs and previous doses of PPV23 negatively influenced fold increase after vaccination^21,25,26^. Lastly, one trial compared splenectomized patients with beta-thalassemia to controls and found no significant difference in the primary response after vaccination with PCV7^28^. Collectively, these included data show that PCV7 and -13 induce a statistically significant increase in PS-specific IgG in asplenic patients.

### Multiple-dose schedules and PPV23 vaccination in asplenic adults

One trial assessed a 2-dose PCV7 schedule and found no significant increase in PS-specific IgG GMCs after the 2nd PCV7 at 6 weeks, except for PS 4^27^. Three trials analyzed the IgG binding antibody response to PPV23 1–2 months after a PCV7 or -13 and reported no significant amplification in PS-specific IgG GMCs for serotypes shared between PPV23 and the PCV^23,27,28^. Similarly, PPV23 as a booster 6–12 months after PCV7 was assessed in three studies, which reported no increase or broadening of the response^23,27,28^. Collectively, these data do not indicate a clear benefit of an additional PCV7 or PPV23 dose in enhancing the IgG binding response in asplenic adults.

### Primary response in asplenic children

In contrast to adults and older children, baseline PS-specific IgG GMCs measured in infants (<1 one year of age) were typically low. Uniformly, primary vaccination with one or several doses of PCV7, PCV10, PCV13, and PCV15 induced a statistically significant increase in PS-specific IgG binding GMCs for most tested PS in all studies. The response was measured 3–12 weeks after the single dose or schedule. Nine trials used a threshold of protection to report their outcomes, and in these, 95.6–97.8% of patients exceeded the WHO threshold of ≥0.35 µg/mL after PCV7 and -10^19,29–32^ and 78–100% exceeded the IgG threshold of ≥1.00 µg/mL after PCV7^29,33^. Four trials included age-matched controls, and these show no significant differences between patients and controls^30–33^. In one of these trials, healthy controls more often had a ≥4-fold increase of GMCs post-PCV compared to patients with SCD; however, baseline GMCs in the SCD group were higher, which probably explains this difference^32^. Collectively, these data show that PCV7, -10, -13, and -15 induce a significant pneumococcal-specific IgG response in asplenic children with no evidence of reduced immunogenicity compared with healthy controls.

### Extended schedules in asplenic children

Several extended childhood vaccination schedules were evaluated, with no observed differences in IgG responses attributable to schedule composition^19,30,31,33–35^. PCV10 was assessed in one trial, which assessed a 2-dose, 3-dose, and 4-dose schedule with no reported differences in the IgG response based on the number of doses^33^. Likewise, PCV7 was assessed in two trials: one assessing a 1-dose and 3-dose schedule and another assessing a 1-dose, 2-dose, and 3-dose schedule, with no reported differences. Notably, intervals, age of the participants, and timing of vaccination differed considerably, which hampers head-to-head comparison between schedules, as can be observed in Tables S1 and S2 (Supplementary Materials 2).

### Booster vaccination with PPV23 or PCV in asplenic children

Two trials compared a single dose of PPV23 to a single- or multiple-dose PCV7 schedule and reported higher IgG GMCs and a broader response (to more PS) after PCV vaccination^19,29^. Boosting with either PPV23 or PCV was more often studied in children compared to adults. PPV23 administered 2–18 months after the first PCV vaccination was assessed in four trials^19,30,34,36^. In contrast to adults, the addition of PPV23 broadened and amplified the PS-specific IgG response in asplenic children with SCD (all around 2 years of age). Two studies assessed a PCV as booster. First, a second PCV13 at 6 months led to a significant increase in IgG levels in children with a mean age of 13.3 years^37^. Second, a booster of PCV10 4–5 months after a 3-dose primary schedule in infants (starting at 6 months of age) did not show a clear increase in PS-specific IgG levels^33^. Due to the difference in age groups, we cannot compare these results. Collectively, PPV23 was shown to enhance and broaden the IgG response in young children with SCD, in contrast to asplenic adults. The booster response of PCVs is still undetermined, as few data are available.

### Longevity of the vaccine response in adults and children

In adults, the longevity of the PCV-induced IgG binding antibody response was assessed in four studies (Table 4): three cross-sectional studies measuring at various time points post vaccination^13,18,24^, and one controlled prospective follow-up study, which assessed antibody persistence 5 years after PCV13^38^. Nived et al. reported above 0.35 µg/mL-threshold GMCs for all PS when measured at a random time point after vaccination. Rosado et al. and Takeshita et al. measured at random time points after vaccination and compared between patients with previous receipt of PPV23 or PCV; patients with previous PCV had significantly higher IgG GMCs^13,18^. However, to draw firm conclusions based on these trials is difficult, as the time since vaccination and splenectomy were not standardized and the outcomes were not compared with baseline levels. In the controlled follow-up study by Papadatou et al., a significant decrease in PS-specific IgG binding GMCs (all remaining above 1.00 µg/mL) was detected 5 years after receipt of PCV13^38^.

**Table 4:** Studies without intervention assessing the longevity of pneumococcal conjugate-induced responses

Study	Patients	*N*	Controls	Vaccination history	Timing measurement	Outcomes
Nived et al.^24^ Sweden Cross-sectional study	Adults Splenectomized	9	-	History of PPV23 (*n* = 28) or PCV13 + PPV23 (*n* = 4)	Not reported	All: IgG GMCs were >0.35 ug/mL for 12/12 PS
Papadatou et al.^38^ Greece Controlled follow-up study	Adults Splenectomy + β-thalassemia	34	-	PCV13	5 years after PCV13 (1)	GMCs of 6/6 PS sig lower compared to day 28 after PCV13 (1 PS dropped below baseline) GMCs were ≥1.0 ug/mL for 5/6 PS and 0.93 ug/mL for 1/6 PS No correlation found between memory B-cell response and previous number of PPV23 (all >5 years previously)
Takeshita et al.^18^ Japan Cross-sectional study	Children + young adults Congenital asplenia Splenectomy	23	100 (HCs)	<5 years: PCV13 (*n* = 1), PCV13 + PPV23 (*n* = 1), PPV23 (*n* = 1) >5 years: PPV23 (*n* = 12) No vaccine (*n* = 5)	< or >5 years after vaccination	All: IgG GMCs were ≥0.2 ug/mL for 6/6 PS in all patients Previous PPV23 sig lower IgG GMCs for 4/6 PS compared to previous PCV7

*N* number of participants, *PPV23* 23-valent pneumococcal polysaccharide vaccine, *PCV* pneumococcal conjugate vaccine, *GMC* geometric mean concentration, *PS* pneumococcal serotype, *sig* statistically significant, *HCs* healthy controls.

In children, residual response prior to boosting was only assessed in studies assessing a multiple-dose extended PCV schedule (Tables S1 and S2, Supplementary Materials). These trials report a significant decrease in the primary IgG binding response when measured 6–18 months after the last dose^30,31,34,37^. No differences between asplenic patients and healthy controls regarding the longevity of earlier induced responses were observed in these pediatric trials. Collectively, in adults, evidence is limited to a single controlled study demonstrating that IgG levels may decline significantly within 5 years after vaccination, but no information is available on the kinetics of this decrease. For young children, data suggest that shortly (6 months) after the last vaccine, a decrease of PS-specific IgG levels can occur, which supports the use of multiple-dose schedules.

### Opsonophagocytic response

The opsonophagocytic (OPA) response was studied in seven prospective studies for PCV7, PCV10, PCV13, and PCV15^20,21,31,33,37,39,40^, using various assays (Table S1, Supplementary Materials). Only one study assessed the OPA response in adults, in patients with SCD^21^. In this study, a schedule of PCV13 followed by PPV23 after 1 month led significantly more often to a ≥4-fold rise of the OPA geometric mean titer (GMT) and to higher GMTs compared to a single dose of PPV23, for PS shared between both vaccines. In children with SCD or splenectomy, the OPA response was assessed in six trials, which uniformly showed a significant increase in opsonophagocytic activity post-PCV7, -10, -13, and -15, with the majority of participants achieving a GMT ≥ 8 for most or all tested PS (Table S3, Supplementary Materials). Notably, two trials found an additional increase in the opsonophagocytic activity after a second dose of PCV10 and -13, while this was not detected in the IgG binding antibody GMCs^20,39^. Accordingly, IgG GMCs only moderately correlated with OPA GMTs^20,31^. No differences in OPA titers could be observed between different multivalent PCVs. One study compared children with SCD to healthy controls and did not find a significant difference^33^. Collectively, these data show that a significant OPA response can be induced after PCV vaccination in both asplenic adults and children. Also, a possible benefit of a second PCV (10 and 13) is suggested by two trials.

### B-cell responses

Three studies assessed B-cell kinetics with ELISPOT (Table 4). Ghazi et al. compared vaccine-specific B-cell responses after administration of PCV13 followed by PPV23 in asplenic adults compared to healthy controls. In this study, asplenic patients had lower PCV13-specific IgG-producing B-cells at baseline but showed a statistically significant increase upon PCV13 vaccination, similar to healthy controls. Rosado et al. assessed the presence of anti-pneumococcal-specific memory B-cells (IgM and IgG) in splenectomized adults and children with a variable vaccination history comprising PPV23 and/or PCVs. They found reduced numbers of IgM and IgG memory B-cells with anti-pneumococcal specificity when the patient was vaccinated with PPV23 at any time point or with PCV7 or -13 prior to splenectomy. By contrast, when vaccinated with PCV after splenectomy, pneumococcal-specific IgM memory B-cells were still reduced, but pneumococcal-specific IgG titers and memory B-cell counts were normal and similar to those of healthy controls. Lastly, Papadatou et al. found a significant increase in the PS-specific IgG memory B-cells starting at 28 days after PCV13. Collectively, these few studies on the PS-specific B-cell response after PCV13 vaccination in asplenic adults show that IgG memory B-cells can be induced by PCVs. For children, data on this topic are lacking.

### Correlates and predictors for vaccine response

In adults, the most often studied variable was previous receipt of PPV23. Three studies found a negative effect of prior PPV23 doses on the response to PCV7 and -13 when administered 2 months to 5 years previously; the fold increase, IgG binding antibody GMCs, and the number of PS-specific IgM memory B-cells were lower when compared with less or no doses of PPV23^25,28,41^.

Two studies found that this negative effect was no longer present when 4–5 years after PPV23 vaccination had passed^38,42^.

For children, one study found a negative effect of previous PPV23 on both GMCs and OPA after PCV. As in adults, two other studies did not find this negative effect when 5 years had passed. Collectively, these studies show a potential effect of previous PPV23 vaccination on the IgG and memory B-cell response to subsequent PCV7 and -13 vaccination in asplenic individuals. However, there is no available information on the exact kinetics or duration of this effect.

### Timing of vaccination

No studies were found investigating the timing of vaccination with PCVs relative to the time of splenectomy.

## Discussion

This systematic review summarizes the currently available data on the immunogenicity of vaccination with PCVs in asplenic and hyposplenic individuals. Patients in the included studies were asplenic due to SCD, congenital asplenia, or underwent splenectomy because of hereditary spherocytosis, trauma, solid organ and hematological malignancies, benign processes requiring resection, and other hematological diseases. The assessed PCVs in this review induced an adequate vaccine-specific IgG binding antibody response between 3 and 12 weeks after primary vaccination in both adults and children. No direct comparison between different underlying conditions was performed in the included studies, but substantial differences are not suggested by the data. In addition, PCV7, -10-, -13, and 15-induced IgG antibodies are shown to have adequate opsonophagocytic functionality, similar to healthy controls. Of note, the included studies assessed the lower-valent PCVs, and the most recently licensed PCVs, such as PCV20 and PCV21, are not represented in the current data. We will discuss the additional findings and identified knowledge gaps in more detail in the following paragraphs.

First, few trials included healthy controls. It remains to be determined whether PCV-induced immunity is significantly impaired in asplenic patients. Currently available data, which compares antibody responses between healthy controls and infants with SCD, do not show reduced immunogenicity of primary and booster PCV7, -10, -13 vaccination in patients. However, the splenic function of infants with SCD is substantially different from that of older patients and patients with complete asplenia after splenectomy. In individuals with SCD, splenic function declines with age due to repeated splenic infarction caused by vaso-occlusion of sickle-shaped erythrocytes^9^. This process starts before the first year of life and eventually leads to auto-splenectomy with complete loss of splenic function during childhood in most SCD patients^9,10^. Therefore, we cannot generalize these outcomes to older patients and patients with other underlying conditions.

Second, data on the longevity of PCV-induced immune responses are limited. In the few available trials, most PCV7 and -13-induced serotype-specific IgG levels remained detectable during the first 5 years after vaccination and declined when 5 years had passed. However, the robustness of this data is limited as four of these trials determined antibody levels at random time points and solely reported whether the vaccination was given less or more than 5 years ago. Notably, a decrease in circulating PS-specific IgG levels after vaccination is also observed in healthy individuals^43,44^. Over time, vaccine-induced protection increasingly depends on recall responses rather than the persistence of circulating antibodies. However, in asplenic patients, the sustained presence of anti-pneumococcal antibodies with opsonophagocytic activity is of particular importance to prevent invasive pneumococcal disease, as these patients lack other first defense mechanisms^4,5^. This stresses the need to monitor vaccine-induced IgG levels in asplenic patients over time in order to assess the need for boosting. Therefore, we recommend evaluating the persistence of anti-pneumococcal IgG and opsonophagocytic activity in asplenic patients from around 5 years post PCV vaccination onward.

Third, the additional benefit of a PCV booster remains unclear for asplenic patients. Trials that assessed a PCV10 and -13 booster after 4–6 months did not show a clear increase in the pneumococcal serotype-specific IgG levels. Nevertheless, two trials showed amplification of the OPA response after boosting, while this was not reflected in significant changes in IgG GMCs^20,39^. These observations reflect the difficulty in fully assessing and quantifying the antibody response after PCV booster vaccination. The booster response cannot be completely captured with assessment of the IgG binding antibody response alone, which mainly reflects (the final) part of vaccine-induced immunity. Each of the subsequent immunological events leading to vaccine-induced immunity can vary and be influenced. The T-cell dependent nature of PCV-induced responses allows for a follicular response, including (re)entering of B-cells in germinal center reactions (GCRs) and subsequent induction of IgM and IgG memory B-cells, long-lived plasma cells, and class-switched B-cells carrying B-cell receptors (BCR) and corresponding immunoglobulins with increased affinity^17^. Theoretically, the sequential PCV doses potentially lead to improvement of the immunological memory and Ig/BCR binding affinity. Knowledge regarding how PCVs induce these GCRs and whether these immunological pathways differ between asplenic and healthy individuals is needed to provide a more complete understanding of the PCV booster response. Additionally, modifiable factors such as the timing of boosting and vaccine formulation in the prevention against invasive pneumococcal disease (IPD) could be identified when assessing these underlying immunological pathways. In this light, few trials assessed PCV-induced B-cell kinetics, and these mainly focused on the vaccine-specific memory B-cell response in the peripheral blood. Papadatou et al. (2014) and Ghazi et al. (2017) show that PCV13 can induce a significant serotype-specific memory B-cell response and thereby induce immunological memory in asplenic patients after primary vaccination. No trials have assessed the booster response at the B-cell level.

Several trials have compared a primary PCV7 or PCV13 to a primary PPV23 or have assessed PPV23 as booster. These trials suggest that PPV23 is less immunogenic than PCVs and provide little evidence of additional benefit when administered 2–12 months after PCV7 or -13 in adult asplenic patients, based on IgG and OPA responses. This is in line with current knowledge and can be explained by specific immunological impairments associated with the asplenic state. In the absence of a functional spleen, the IgM memory B-cell pool is reduced, which leads to reduced immunogenicity of T-cell independent pneumococcal polysaccharide vaccines, as observed in this review^4,5,14,45,46^. In contrast to adults, a PPV23 booster 11–12 months after the last PCV in children with SCD (≥2 years of age) led to both amplification and broadening of the earlier induced PCV response in these patients. This difference can probably be explained by the presence of residual splenic function early in life of patients with SCD. For children with splenectomy and hence no residual splenic function, PPV23 immunogenicity is expected to resemble that of asplenic adults, as described in this review. Overall, the available data suggest little benefit of PPV23 vaccination for asplenic adults in terms of boosting or broadening the response, although the data are limited.

No comparative trials were found that focused on the optimal timing of vaccination around splenectomy. Currently, it is recommended to vaccinate prior to splenectomy, and this is widely employed^47,48^. However, there are limited data supporting this practice, and it is primarily based on empirical reasoning. It remains to be determined whether it is beneficial to engage splenic function, as an important secondary lymphoid organ, in the primary vaccine response or whether to vaccinate after splenectomy and thereby prevent the removal of newly vaccine-induced immunological memory homed in the spleen. Rosado et al. (2013) described reduced pneumococcal-specific IgG memory B-cell counts in patients vaccinated with PCV7 or PCV13 prior to splenectomy versus patients vaccinated post-splenectomy^13^. However, this was not a controlled study, and intervals between sampling, vaccination, and splenectomy were not standardized. Future studies should focus on differences in the vaccination response when vaccinating pre- or post-splenectomy and consider both antibody and B-cell responses. Theoretically, by removing the spleen, a substantial part of the total B-cell pool is taken out, as a significant part of B-cells home to the spleen^13,49,50^. As a result, a part of newly induced memory B-cells after PCV vaccination could be removed by splenectomy, which potentially translates into lower recall responses after boosting later on. It is unclear how significant this is in the clinical setting.

This review has some limitations due to factors that affect data quality and decrease the generalizability of the outcomes. First, the trial populations were heterogeneous in several studies because they included patients with varying asplenia-inducing conditions or different indications for splenectomy^24,27,29,42^. In addition, an adequate subgroup analysis was often not performed or was not possible due to a small sample size in these studies (e.g., for congenital asplenia). Also, controls were included in the minority of trials and substantially more often in pediatric trials. These factors all decrease the ability to generalize the outcomes to specific patient groups and to compare the outcomes with healthy individuals. Furthermore, for some underlying conditions (e.g., specific inherited forms of asplenia such as polysplenia secondary to heterotaxy syndrome), studies were not available, and the outcomes of this review cannot be directly generalized to these groups. For patients with conditions known to induce functional asplenia in some patients (e.g., celiac disease), vaccine studies were available but could not be included in this review because the degree of asplenia was not measured. Also, to patients who are additionally immunocompromised beside the asplenic-state, conclusions of this review cannot directly apply. For these, one should combine the effects of asplenia with those of the other immunocompromising condition(s) in order to formulate conclusions and recommendations regarding vaccination. Furthermore, the timing of response measurement (3–12 weeks) and the applied laboratory assays used to measure the serological response and OPA responses varied between studies. Hereby, a head-to-head comparison between the outcomes of different studies and the merging of data are hampered.

An additional factor complicating pneumococcal vaccination research in general is the current lack of a validated and internationally accepted correlate of protection or response. As a result, outcomes used to assess the vaccine response and the definitions of protection are highly variable between studies, as reflected in this review. This additionally complicates the comparison and merging of the outcomes. To account for this, we primarily focused on an increase in PS-specific IgG or OPA levels after vaccination as a measure of the response without inferring a direct correlation with protection. The World Health Organization recommends a serotype-specific IgG binding antibody threshold of 0.35 µg/mL, which is based on pooled data from three trials assessing PCV7 in children in an era when PCVs were newly introduced^22^. Recently, higher thresholds such as 1.3 µg/mL have been applied, especially for immunocompromised individuals^51^. Outcomes on thresholds (such as % achieving a threshold) were added as a supplement to allow comparison with other articles and not for primary interpretation. Of note, an outcome based on IgG does not directly reflect functionality or durability of the response. The OPA response reflects functionality of pneumococcal-specific antibodies and provides better insight than binding antibodies alone, and only moderately correlated with IgG in this review. Additionally, vaccine-specific immune cells (mostly memory B-cells) and associated induced pathways can be studied to improve our understanding of the overall performance of PCVs. In our opinion, future studies should aim to capture determinants of the entire process by studying the immunological mechanisms leading to immunity and memory, such as measurement of memory B-cells and assessment of the GCR after PCV vaccination, rather than focus on the end products.

In the near future, higher valent PCVs are expected to replace PPV23 in the guidelines for asplenic patients as their serotype coverage is approaching that of PPV23^52^. The included data in this review suggest a lack of benefit of PPV23 in asplenic adults, which could support this change in strategy. By contrast, for children with SCD, PPV23 was beneficial through broadening and boosting earlier PCV responses. However, this review shows a consistent response to PCV vaccination in both asplenic adults and children. When we combine these observations with the benefits of PCVs, particularly their ability of inducing long-term memory responses, we expect that PCVs will also become the preferred vaccine for asplenic children in the future.

Based on this review, multiple knowledge gaps regarding pneumococcal conjugate vaccination in asplenic patients are identified. These can be divided into two domains. First, we need more sufficiently powered prospective controlled clinical trials that assess the newest PCVs and evaluate the optimal timing for vaccination around splenectomy. In these studies, an additional focus should lie on the effect of the increased valency on Ig levels to assess whether limits exist in the number of antigens to which the immune system can respond simultaneously. Particular effort should be made to improve generalizability through the selection of more homogeneous trial populations and adequate reporting of other factors known to impact vaccine immunogenicity, such as additional immunocompromising conditions and active malignancies. Second, we need to improve our fundamental immunological knowledge of the asplenic state and the role of the spleen in vaccine responses. Exploration of how PCVs induce immunological memory in asplenic patients by studying GCRs, vaccine-specific memory B-cells, class switching, and long-lived plasma cells will provide more information about the sustainability and quality of these vaccine responses. Additionally, investigating whether subsequent PCV doses improve the affinity of B-cell receptors and immunoglobulins and induce class switching will substantiate whether boosting is beneficial. Sampling of GCRs by fine needle aspiration has been proven feasible and well tolerated in clinical studies when using minimally invasive fine needle aspiration, as described in several studies assessing COVID-19 and seasonal influenza vaccines^53–57^.

In conclusion, PCVs seem to be on the right track to become the main players in preventing IPD for asplenic and hyposplenic populations, and data from this review support the use of the higher valent PCVs in these individuals. Data on the optimal timing of vaccination around splenectomy are lacking. The impact of the asplenic state on the immunogenicity of these vaccines remains to be unraveled in the future. Filling these knowledge gaps may provide possibilities to further improve the prevention of IPD in asplenic patients.

## Methods

We followed the Preferred Reporting Items for Systematic Reviews and Meta-Analyses (PRISMA 2020) methodology^58^. The review protocol was registered and published in the online PROSPERO register (ID: CRD42024600319).

### Search

On November 5th, 2024, the search was performed on PubMed, EMBASE, and Web of Science. To capture pre-licensure studies, we included studies from 1990 onward. A final update of the search was performed on the 1st of August 2025, with no additional articles identified. The outcome of the search was uploaded in Rayyan software, after which duplicates were removed.

### Search strategy

The search strategy was constructed with the help of a librarian and was based on our clinical question: What is the immunogenicity of PCVs in asplenic and hyposplenic individuals? A corresponding PICO (Supplementary Materials 1.1) was constructed, which served as the base of the search. The search strategy, including entry terms, can be found in the Supplementary Materials 1.1. Snowballing was applied to all included articles to complement the systematic search.

### Inclusion and exclusion criteria

Studies assessing the immunogenicity of PCVs in patients with asplenia and documented hyposplenia were included. Asplenia was defined as the absence of splenic function following splenectomy. Hyposplenia was defined as reduced splenic function as determined by the presence of Howell-Jolly bodies or a pitted red cell count >4% in the peripheral blood. Individuals with sickle cell disease were considered hyposplenic, regardless of whether splenic function was measured. Individuals of all ages were included. Vaccination could either be a primary or booster regimen and contained in a single- or multiple-dose schedule. Studies reporting immunogenicity outcomes (such as the IgG binding antibody response, the opsonophagocytic activity (OPA), B-cell response) that have been correlated directly or indirectly to efficacy were eligible for inclusion. Studies assessing asplenia in otherwise immunocompromised populations (e.g., use of immunosuppressive treatment, presence of a concomitant hematological malignancy, and/or primary or secondary immune disorder) were excluded because we aimed to isolate the effect of asplenia on vaccine responses as much as possible. Also, case reports and case series with a sample size <10 were not included.

### Screening and data extraction

After removal of duplicates, articles were screened by title and abstract and remaining articles by full text. The screening was performed independently by two researchers (B.P. and H.J.). Discrepancies were solved by consensus. In case no consensus was achieved, a third researcher (G.L.S.S.) was available for consultation. Inclusion and exclusion criteria predefined in the protocol were applied during this process. Data to be collected was predefined in the protocol in a collection table and comprised: name of first author, year of the study, study design (with vaccine and schedule assessed), population (number of patient participants and healthy controls), and underlying disease. The following outcomes were collected: number of participants, vaccine type and vaccination schedule, and immunogenicity. Immunogenicity outcomes could be either pneumococcal serotype-specific IgG binding antibody titer and derivates such as fold rise and thresholds of protection, OPA titer and proportion above a certain OPA titer, or B-cell responses such as pneumococcal serotype(PS)-specific memory B-cell response.

### Risk assessment

The risk of bias was assessed independently for each included article by two researchers (G.L.S.S. and B.P.). In case no consensus was achieved, a third researcher (H.J.) was available for consultation. For randomized controlled trials, the Cochrane Risk of Bias (RoB 2) was applied, categorizing the risk of bias as low, some concerns, or high (Supplementary Materials 1.2). The risk of bias was determined using the Rob2 tool for individually randomized parallel-group trials (*n* = 7) or individually randomized cross-over trials (*n* = 1). For nonrandomized clinical trials, the Newcastle-Ottawa scale (NOS) was used, categorizing the trials as good, fair, or poor. Briefly, NOS uses stars to assess each domain; the domain of comparability can be scored with a maximum of two stars and the other domains with a maximum of one star (Supplementary Materials 1.3). Discrepancies were solved by consensus.

### Data analysis

Data are summarized, presented, and compared in a systematic manner, using tables comprising study characteristics, immunological outcomes (when possible stratified by serotype), immunological assessments/techniques used, and criteria for adequate response (when applied in the study). If possible, data are stratified by type of vaccine, patient group, underlying condition, and schedule. We did not perform a meta-analysis due to differences in applied assays and outcomes (such as the definition of response), which prohibited the merging of these data in a single analysis.

## Supplementary information

Supplementary information

## Data Availability

All data that were used to generate conclusion have been included in the manuscript and/or supplementary materials.
