# Supplementary material for: The immunogenicity of pneumococcal conjugate vaccinations in patients with asplenia and hyposplenia: a systematic review
Source: NPJ Vaccines. 2026 Jul 6;11:199. doi: 10.1038/s41541-026-01514-9 (PMC13714245; doi:10.1038/s41541-026-01514-9)
Supplement: Supplementary file 1 [file 41541_2026_1514_MOESM1_ESM.pdf]

Supplementary materials: *The immunogenicity of pneumococcal conjugate vaccinations in patients with asplenia and hyposplenia: a systematic review*

Supplementary Materials 1

1. The search strategy
2. Risk of Bias Randomized controlled trials
3. Risk of Bias Nonrandomized trials for cohort studies
4. PRISMA checklist

Supplementary Materials 2

1. Table S1 Study characteristics
2. Table S2 Outcomes per study
3. Table S3 all immunological outcomes per study

1. Search Strategy and PICO

Review question

**Full review question**<br>Provide the full review question in sentence format, and then break up the question according to the PICO framework (or other frameworks as appropriate).	
The immunogenicity of pneumococcal conjugate vaccines in patients with asplenia, demonstrated hyposplenism or sickle cell disease (always accompanied by hyposplenism).	
**Population**	Adult or paediatric patients with asplenia (post-splenectomy or congenital), demonstrated hyposplenism (Howell Jolly Bodies or PRC,) or with sickle cell disease.
**Intervention**	Primary or booster vaccination with pneumococcal conjugate vaccine
**Comparison**	Not applicable
**Outcome**	Immunogenicity, described as serological response (vaccine-induced PS-specific IgG and/or IgM GMCs), opsonophagocytic capacity (OPA) GMCs of vaccine-induced antibodies, vaccine-induced PS-specific B cell kinetics.

<u>Pubmed</u>

P	"Spleen"[Mesh] OR "Splenectomy"[Mesh] OR "Splenectom*"[tw] OR "Splenic Diseases"[Mesh] OR "Asplen*"[tw] OR "Hyposplen*"[tw] OR "Splenic hypofunction*"[tw] OR "Splenic Infarction"[Mesh] OR "Splenic Infarction*"[tw] OR "Anemia, Sickle Cell"[Mesh] OR "Sickle Cell disease"[tw] OR "Sickle Cell diseases"[tw] OR "hyposplenia*"[tw] OR "Sickle Cell anaemia*"[tw] OR "beta-Thalassemia"[Mesh] OR "beta-Thalass*"[tw] OR "betaThalass*"[tw] OR "Celiac Disease"[Mesh] OR "Celiac Disease*"[tw] OR "Inflammatory Bowel Diseases"[Mesh] OR "Inflammatory Bowel Disease*"[tw] OR "Colitis, Ulcerative"[Mesh] OR "Ulcerative Colitis"[tw] OR "Crohn Disease"[Mesh] OR "Crohn Disease*"[tw] OR ("Spleen"[Mesh] OR "Spleen"[tw] AND "Embolization, Therapeutic"[Mesh] OR "Therapeutic Embolization"[tw] OR "Therapeutic Embolisation"[tw]) OR "Splenic embolization"[tw] OR "Splenic embolisation"[tw] OR "Whipple Disease"[tw] OR "Lupus Erythematosus, Systemic"[Mesh] OR "systemic lupus erythematosus"[tw]
I	"Vaccines"[Mesh] OR "Vaccine*"[tw] AND "Streptococcus pneumoniae"[Mesh] OR "Streptococcus pneumoniae"[tw]) OR "Pneumococcal Vaccines"[Mesh] OR "Pneumococcal Vaccine*"[tw] OR ("Streptococcus pneumoniae"[Mesh] OR "Streptococcus pneumonia*"[tw] AND "Vaccines, Conjugate"[Mesh] OR "Conjugate Vaccine*"[tw] OR "Conjugated Vaccine*"[tw]) OR "Pneumococcal conjugate

	vaccine*"[tw] OR "Pneumococcal conjugated vaccine*"[tw] OR "PCV7"[tiab] OR "PCV10"[tiab] OR "PCV13"[tiab] OR "PCV15"[tiab] OR "PCV20"[tiab] OR " Synflorix"[tw] OR "Vaxneuvance"[tw] OR " Vaxneuvance"[tw] OR "Apexxnar"[tw] OR "Pnu-Imune Vaccine*"[tw] OR "Pnu Imune Vaccine*"[tw] OR "Pneumococcal Polysaccharide Vaccine*"[tw] OR "Pneumovax*"[tw]
C	*N.A.*
O	Not included in the search, according to advice of librarian, as this would narrow the search down too much.

Executed: 05-11-2024
By: Bridget van Put
Hits: 1648
Hits after application of time window 01-01-1990 until 05-11-2024 and selection of article type: 276

<u>Web of Science</u>

P	1: TS=("Splenectom*" OR "Asplen*" OR "Hyposplen*" OR "Splenic hypofunction*" OR "Splenic Infarction*" OR "Sickle Cell disease" OR "Sickle Cell diseases" OR "hyposplenia*" OR "Sickle Cell anaemia*" OR "beta-Thalass*" OR "betaThalass*" OR "Celiac Disease*" OR "Inflammatory Bowel Disease*" OR "Ulcerative Colitis" OR "Crohn Disease*" OR ("Spleen" AND "Therapeutic Embolization" OR "Therapeutic Embolisation") OR "Splenic embolization" OR "Splenic embolisation" OR "Whipple Disease" OR "systemic lupus erythematosus")
I	2: TS=(("Vaccine*" AND "Streptococcus pneumoniae") OR "Pneumococcal Vaccine*" OR ("Streptococcus pneumonia*" AND "Conjugate Vaccine*" OR "Conjugated Vaccine*") OR "Pneumococcal conjugate vaccine*" OR "Pneumococcal conjugated vaccine*" OR "PCV7" OR "PCV10" OR "PCV13" OR "PCV15" OR "PCV20" OR " Synflorix" OR "Vaxneuvance" OR " Vaxneuvance" OR "Apexxnar" OR "Pnu-Imune Vaccine*" OR "Pnu Imune Vaccine*" OR "Pneumococcal Polysaccharide Vaccine*" OR "Pneumovax*")
C	*N.A.*
O	Not included in the search, according to advice of librarian, as this would narrow the search down too much.

Executed: 05-11-2024
By: Bridget van Put
Hits: 791
Hits after application of time window 01-01-1990 until 05-11-2024 and selection of article type: 674

Supplementary materials: *The immunogenicity of pneumococcal conjugate vaccinations in patients with asplenia and hyposplenia: a systematic review*

Embase

# ▼	Searches	Results	Type	Actions	
19	18 and "Article" [Publication Type]	1295	Advanced	Display Results	More
18	17 and 1990:2025.(sa_year).	2659	Advanced	Display Results	More
17	13 and 16	2978	Advanced	Display Results	More
16	14 or 15	31334	Advanced	Display Results	More
15	("Pneumococcal conjugate vaccine*" or "Pneumococcal conjugated vaccine*" or "PCV7" or "PCV10" or "PCV13" or "PCV15" or "PCV20" or " Synflorix" or "Vaxneuvance" or " Vaxneuvance" or "Apexxnar" or "Pnu-Imune Vaccine*" or "Pnu Imune Vaccine*" or "Pneumococcal Polysaccharide Vaccine*" or "Pneumovax*").ti,ab.	9030	Advanced	Display Results	More
14	Pneumococcus vaccine/ or ((Streptococcus pneumoniae/ or Streptococcus pneumonia/ or "Streptococcus pneumoniae".ti,ab. or "Streptococcus pneumonia".ti,ab. or " Pneumococcal".ti,ab.) and (vaccine/ or conjugate vaccine/ or "Vaccine*".ti,ab. or "Conjugate Vaccine*".ti,ab. or "Conjugated Vaccine*".ti,ab.))	31202	Advanced	Display Results	More
13	1 or 2 or 3 or 4 or 5 or 6 or 7 or 8 or 9 or 10 or 11 or 12	715118	Advanced	Display Results	More
12	("Splenic embolization" or "Splenic embolisation" or "Whipple Disease" or "systemic lupus erythematosus").ti,ab.	89781	Advanced	Display Results	More
11	(("Spleen" and "Therapeutic Embolization") or "Therapeutic Embolisation").ti,ab.	101	Advanced	Display Results	More
10	("Splenectom*" or "Asplen*" or "Hyposplen*" or "Splenic hypofunction*" or "Splenic Infarction*" or "Sickle Cell disease" or "Sickle Cell diseases" or "hyposplenia*" or "Sickle Cell anaemia*" or "beta-Thalass*" or "betaThalass*" or "Celiac Disease*" or "Inflammatory Bowel Disease*" or "Ulcerative Colitis" or "Crohn Disease*").ti,ab.	269000	Advanced	Display Results	More
9	systemic lupus erythematosus.mp. or systemic lupus erythematosus/	131806	Advanced	Display Results	More
8	artificial embolization.mp. or artificial embolism/ or artificial embolization/	71823	Advanced	Display Results	More
7	inflammatory bowel disease.mp. or ulcerative colitis/ or inflammatory bowel disease/ or Crohn disease/	239009	Advanced	Display Results	More
6	celiac disease.mp. or celiac disease/	40804	Advanced	Display Results	More
5	beta thalassemia.mp. or beta thalassemia/	22768	Advanced	Display Results	More
4	sickle cell anemia.mp. or sickle cell anemia/	46778	Advanced	Display Results	More
3	partial splenectomy/ or splenectomy/ or splenectomy.mp.	47797	Advanced	Display Results	More
2	spleen defect/ or spleen infarction/ or spleen surgery/ or spleen injury/ or spleen/ or spleen disease.mp. [mp=title, abstract, heading word, drug trade name, original title, device manufacturer, drug manufacturer, device trade name, keyword heading word, floating subheading word, candidate term word]	135490	Advanced	Display Results	More
1	1 spleen defect/ or spleen infarction/ or spleen surgery/ or spleen injury/ or spleen/ or spleen disease/	135274	Advanced	Display Results	More

Executed: 05-11-2024
Embase
By: Bridget van Put
Hits: 2978
Hits after application of time window 01-01-1990 until 05-11-2024 and selection of article type: 1295

2. Assessment of the Risk of Bias for randomized controlled trials using the Rob 2 tool

Revised Cochrane risk-of-bias tool for randomized trials (RoB 2) TEMPLATE FOR COMPLETION

Edited by Julian PT Higgins, Jelena Savović, Matthew J Page, Jonathan AC Sterne
on behalf of the RoB2 Development Group

Version of 22 August 2019

The development of the RoB 2 tool was supported by the MRC Network of Hubs for Trials Methodology Research (MR/L004933/2- N61), with the support of the host MRC ConDuCT-II Hub (Collaboration and innovation for Difficult and Complex randomised controlled Trials In Invasive procedures - MR/K025643/1), by MRC research grant MR/M025209/1, and by a grant from The Cochrane Collaboration.

This work is licensed under a Creative Commons Attribution-NonCommercial-NoDerivatives 4.0 International License.

Study details

Reference

Vernacchio L, Neufeld EJ, MacDonald K, Kurth S, Murakami S, Hohne C, King M, Molrine D. Combined schedule of 7-valent pneumococcal conjugate vaccine followed by 23-valent pneumococcal vaccine in children and young adults with sickle cell disease. J Pediatr. 1998 Aug;133(2):275-8. doi: 10.1016/s0022-3476(98)70235-5. PMID: 9709721.

Study design

- X Individually-randomized parallel-group trial
- ☐ Cluster-randomized parallel-group trial
- ☐ Individually randomized cross-over (or other matched) trial

For the purposes of this assessment, the interventions being compared are defined as

Experimental:	Extended schedule: PCV7+PCV7+PPV23	Comparator:	One dose of PPV23

**Specify which outcome is being assessed for risk of bias**	Height of pneumococcal serotype specific IgG antibody concentration (immunogenicity)
**Specify the numerical result being assessed.** In case of multiple alternative analyses being presented, specify the numeric result (e.g. RR = 1.52 (95% CI 0.83 to 2.77) and/or a reference (e.g. to a table, figure or paragraph) that uniquely defines the result being assessed.	Pneumococcal serotype specific IgG Geometric Mean Concentration in ug/mL.

Is the review team's aim for this result…?

- ☐ to assess the effect of ***assignment to intervention*** (the 'intention-to-treat' effect)
- ☐ ~~to assess the effect of *adhering to intervention* (the 'per-protocol' effect)~~

If the aim is to assess the effect of *adhering to intervention*, select the deviations from intended intervention that should be addressed (at least one must be checked):

- ☐ occurrence of non-protocol interventions
- ☐ failures in implementing the intervention that could have affected the outcome
- ☐ non-adherence to their assigned intervention by trial participants

Which of the following sources were obtained to help inform the risk-of-bias assessment? (tick as many as apply)

- ☐ **Journal article(s) with results of the trial**
- ☐ Trial protocol
- ☐ Statistical analysis plan (SAP)
- ☐ Non-commercial trial registry record (e.g. ClinicalTrials.gov record)
- ☐ Company-owned trial registry record (e.g. GSK Clinical Study Register record)
- ☐ "Grey literature" (e.g. unpublished thesis)
- ☐ Conference abstract(s) about the trial
- ☐ Regulatory document (e.g. Clinical Study Report, Drug Approval Package)
- ☐ Research ethics application
- ☐ Grant database summary (e.g. NIH RePORTER or Research Councils UK Gateway to Research)
- ☐ Personal communication with trialist
- ☐ Personal communication with the sponsor

Risk of bias assessment

Responses underlined in green are potential markers for low risk of bias, and responses in red are potential markers for a risk of bias. Where questions relate only to sign posts to other questions, no formatting is used.

Domain 1: Risk of bias arising from the randomization process

Signalling questions	Comments	Response options
**1.1 Was the allocation sequence random?**	No information (text: 'subjects were randomized to receive a single dose of 23-valent pneumococcal vaccine or two doses of 7-valent pneumococcal-CRM197 conjugate vaccine). Nothing stated about randomization method.	NI
**1.2 Was the allocation sequence concealed until participants were enrolled and assigned to interventions?**		NI

**1.3 Did baseline differences between intervention groups suggest a problem with the randomization process?**	Baseline characteristics seem to be logically balanced between groups.	N
**Risk-of-bias judgement**		Some concerns
Optional: What is the predicted direction of bias arising from the randomization process?	It is not clear how participants are randomized, however there are no signs of problems or irregularities in the randomization process.	Unpredictable

Domain 2: Risk of bias due to deviations from the intended interventions (*effect of assignment to intervention*)

**Signalling questions**	**Comments**	**Response options**
**2.1. Were participants aware of their assigned intervention during the trial?**	Two schedules comprising different amounts of vaccination (1 vs 3) were administered without use of placebo. Furthermore it is not stated whether administered of the vaccines were blinded for type of vaccine or timepoint in the schedule.	Y
**2.2. Were carers and people delivering the interventions aware of participants' assigned intervention during the trial?**		PY
**2.3. If Y/PY/NI to 2.1 or 2.2: Were there deviations from the intended intervention that arose because of the trial context?**	It is stated that all participants completed the study and the samples from all participants were available. It seems that there were no deviations. The N was unequal (N=23) and participant numbers in the demographics table match those of the outcome table.	PN
**2.4 If Y/PY to 2.3: Were these deviations likely to have affected the outcome?**	*N.A.*	NA
**2.5. If Y/PY/NI to 2.4: Were these deviations from intended intervention balanced between groups?**	*N.A.*	NA

**2.6 Was an appropriate analysis used to estimate the effect of assignment to intervention?**		PY
**2.7 If N/PN/NI to 2.6: Was there potential for a substantial impact (on the result) of the failure to analyse participants in the group to which they were randomized?**		NA
**Risk-of-bias judgement**		Low
Optional: What is the predicted direction of bias due to deviations from intended interventions?		Towards null

Domain 3: Missing outcome data

Signalling questions	Comments	Response options
**3.1 Were data for this outcome available for all, or nearly all, participants randomized?**	All completed the trial and for all data is available.	Y
**3.2 If N/PN/NI to 3.1: Is there evidence that the result was not biased by missing outcome data?**		NA
**3.3 If N/PN to 3.2: Could missingness in the outcome depend on its true value?**		NA
**3.4 If Y/PY/NI to 3.3: Is it likely that missingness in the outcome depended on its true value?**		NA

**Risk-of-bias judgement**		Low
Optional: What is the predicted direction of bias due to missing outcome data?		Towards null

Domain 4: Risk of bias in measurement of the outcome

Signalling questions	Comments	Response options
**4.1 Was the method of measuring the outcome inappropriate?**	Outcomes seem to be pre-specified and according to international standards, measured by an internationally accepted assay (ELISA with pre-absorption of C-polysaccharide, standardized with reference plasma pool 89-SF (Food and Drug Administration, Bethesda, Md.). However, still there is no consensus concerning the best method of measuring the pneumococcal vaccine response.	PN
**4.2 Could measurement or ascertainment of the outcome have differed between intervention groups?**	Measurement of the primary outcome was performed on the same moment. Also, the primary outcome comprises a lab assay result. Also, the same assay was used.	PN
**4.3 If N/PN/NI to 4.1 and 4.2: Were outcome assessors aware of the intervention received by study participants?**	It is not stated whether outcome assessor were blinded.	NI
**4.4 If Y/PY/NI to 4.3: Could assessment of the outcome have been influenced by knowledge of intervention received?**	Assessment can be influenced when the assessor is not blinded for treatment allocation, as a few steps in the assay process (ELISA) can ben performed differently when having this knowledge. Therefore, it is not possible to exclude.	PY
**4.5 If Y/PY/NI to 4.4: Is it likely that assessment of the outcome was influenced by knowledge of intervention received?**	It is not possible to exclude but it does not seem likely that outcomes were influenced. However, it seems more likely that assessors were blinded but this information is lacking, and therefore we cannot answer this question and we choose PN.	PN

**Risk-of-bias judgement**		Some concerns
Optional: What is the predicted direction of bias in measurement of the outcome?	Due to lack of information	Unpredictable

Domain 5: Risk of bias in selection of the reported result

Signalling questions	Comments	Response options
**5.1 Were the data that produced this result analysed in accordance with a pre-specified analysis plan that was finalized before unblinded outcome data were available for analysis?**	Unclear in absence of available statistical plan or protocol. However, methods section describes the statistics used to analyze the results.	NI
**Is the numerical result being assessed likely to have been selected, on the basis of the results, from...**		
**5.2. ... multiple eligible outcome measurements (e.g. scales, definitions, time points) within the outcome domain?**	It is a generally accepted and widely used parameter, resulting from simple statistics.	PN
**5.3 ... multiple eligible analyses of the data?**		PN
**Risk-of-bias judgement**		Some concerns

Optional: What is the predicted direction of bias due to selection of the reported result?		Towards null

Overall risk of bias

**Risk-of-bias judgement**	Multiple domains are scored with some concerns, however based on the basis of those rankings it is not likely that bias should be scored as an overall high risk of bias.	**Some concerns**
Optional: What is the overall predicted direction of bias for this outcome?		Towards null

This work is licensed under a Creative Commons Attribution-NonCommercial-NoDerivatives 4.0 International License.

Study details

**Reference**	**Vernacchio L, Romero-Steiner S, Martinez JE, MacDonald K, Barnard S, Pilishvili T, Carlone GM, Ambrosino DM, Molrine DC. Comparison of an opsonophagocytic assay and IgG ELISA to assess responses to pneumococcal polysaccharide and pneumococcal conjugate vaccines in children and young adults with sickle cell disease. J Infect Dis. 2000 Mar;181(3):1162-6. doi: 10.1086/315307. PMID: 10720547.**

Study design

- X Individually-randomized parallel-group trial
- ☐ Cluster-randomized parallel-group trial
- ☐ Individually randomized cross-over (or other matched) trial

For the purposes of this assessment, the interventions being compared are defined as

Experimental:	Extended schedule: PCV7+PCV7+PPV23	Comparator:	One dose of PPV23

**Specify which outcome is being assessed for risk of bias**	Serotype-specific anti-pneumococcal 0psonophagocytic activity (OPA)
**Specify the numerical result being assessed.** In case of multiple alternative analyses being presented, specify the numeric result (e.g. RR = 1.52 (95% CI 0.83 to 2.77) and/or a reference (e.g. to a table, figure or paragraph) that uniquely defines the result being assessed.	OPA titer in ug/mL

Is the review team's aim for this result…?

- ☐ to assess the effect of *assignment to intervention* (the 'intention-to-treat' effect)
- ☐ ~~to assess the effect of *adhering to intervention* (the 'per-protocol' effect)~~

If the aim is to assess the effect of *adhering to intervention*, select the deviations from intended intervention that should be addressed (at least one must be checked):

- ☐ occurrence of non-protocol interventions
- ☐ failures in implementing the intervention that could have affected the outcome
- ☐ non-adherence to their assigned intervention by trial participants

Which of the following sources were obtained to help inform the risk-of-bias assessment? (tick as many as apply)

☐ **Journal article(s) with results of the trial**
☐ Trial protocol
☐ Statistical analysis plan (SAP)
☐ Non-commercial trial registry record (e.g. ClinicalTrials.gov record)
☐ Company-owned trial registry record (e.g. GSK Clinical Study Register record)
☐ “Grey literature” (e.g. unpublished thesis)
☐ Conference abstract(s) about the trial
☐ Regulatory document (e.g. Clinical Study Report, Drug Approval Package)
☐ Research ethics application
☐ Grant database summary (e.g. NIH RePORTER or Research Councils UK Gateway to Research)
☐ Personal communication with trialist
☐ Personal communication with the sponsor

Risk of bias assessment

Responses underlined in green are potential markers for low risk of bias, and responses in red are potential markers for a risk of bias. Where questions relate only to sign posts to other questions, no formatting is used.

Domain 1: Risk of bias arising from the randomization process

Signalling questions	Comments	Response options
**1.1 Was the allocation sequence random?**	No information (text: ‘subjects were randomized to receive a single dose of 23-valent pneumococcal vaccine or two doses of 7-valent pneumococcal-CRM197 conjugate vaccine). Nothing stated about randomization method.	NI
**1.2 Was the allocation sequence concealed until participants were enrolled and assigned to interventions?**		NI

**1.3 Did baseline differences between intervention groups suggest a problem with the randomization process?**		N
**Risk-of-bias judgement**		Some concerns
Optional: What is the predicted direction of bias arising from the randomization process?		Unpredictable

Domain 2: Risk of bias due to deviations from the intended interventions (*effect of assignment to intervention*)

Signalling questions	Comments	Response options
**2.1. Were participants aware of their assigned intervention during the trial?**	Two schedules comprising different amounts of vaccination (1 vs 3) were administered without use of placebo. Furthermore it is not stated whether administered of the vaccines were blinded for type of vaccine or timepoint in the schedule.	Y
**2.2. Were carers and people delivering the interventions aware of participants' assigned intervention during the trial?**		PY
**2.3. If Y/PY/NI to 2.1 or 2.2: Were there deviations from the intended intervention that arose because of the trial context?**	It is stated that all participants completed the study and the samples from all participants were available. It seems that there were no deviations. The N was unequal (N=23) and participant numbers in the demographics table match those of the outcome table.	PN
**2.4 If Y/PY to 2.3: Were these deviations likely to have affected the outcome?**	*N.A.*	NA

**2.5. If Y/PY/NI to 2.4: Were these deviations from intended intervention balanced between groups?**	*N.A.*	NA
**2.6 Was an appropriate analysis used to estimate the effect of assignment to intervention?**		PY
**2.7 If N/PN/NI to 2.6: Was there potential for a substantial impact (on the result) of the failure to analyse participants in the group to which they were randomized?**	*N.A.*	NA
**Risk-of-bias judgement**		Low
Optional: What is the predicted direction of bias due to deviations from intended interventions?		Towards null

Domain 3: Missing outcome data

**Signalling questions**	**Comments**	**Response options**
**3.1 Were data for this outcome available for all, or nearly all, participants randomized?**	All completed the trial and for all participants the ELISA results are available in the primary article where to an adequate reference has been made. It is stated in the methods that from all participants at all timepoint samples have been collected. However, in this article the number of participants from which samples have been collected is not numerically stated, and therefore it is scored with a PY instead of a Y.	PY
**3.2 If N/PN/NI to 3.1: Is there evidence that the result was not biased by missing outcome data?**	*N.A.*	NA
**3.3 If N/PN to 3.2: Could missingness in the outcome depend on its true value?**	*N.A.*	NA

**3.4 If Y/PY/NI to 3.3: Is it likely that missingness in the outcome depended on its true value?**		NA
**Risk-of-bias judgement**		Low
Optional: What is the predicted direction of bias due to missing outcome data?		Towards null

Domain 4: Risk of bias in measurement of the outcome

**Signalling questions**	**Comments**	**Response options**
**4.1 Was the method of measuring the outcome inappropriate?**	Outcomes seem to be pre-specified and measured with a well described OPA flowcytometry assay and measured by an internationally accepted assay (ELISA with pre-absorption of C-polysaccharide, standardized with reference plasma pool 89-SF (Food and Drug Administration, Bethesda, Md.).	PN
**4.2 Could measurement or ascertainment of the outcome have differed between intervention groups?**	Measurement of the primary outcome was performed on the same moment. Also, the primary outcome comprises a lab assay result and the same assay was constantly used.	PN
**4.3 If N/PN/NI to 4.1 and 4.2: Were outcome assessors aware of the intervention received by study participants?**	It is not stated whether assessors were blinded.	NI
**4.4 If Y/PY/NI to 4.3: Could assessment of the outcome have been influenced by knowledge of intervention received?**	Assessment can be influenced when the assessor is not blinded for treatment allocation, as a few steps in the assay processes can be performed differently when having this knowledge. Therefore, it is not possible to exclude.	PY
**4.5 If Y/PY/NI to 4.4: Is it likely that assessment of the outcome was**	It is not possible to exclude but it does not seem likely that outcomes were influenced. However, it seems more likely that assessors were blinded but	PN

**influenced by knowledge of intervention received?**	this information is lacking, and therefore we cannot answer this question and we choose PN.	
**Risk-of-bias judgement**		Some concerns
Optional: What is the predicted direction of bias in measurement of the outcome?		Unpredictable

Domain 5: Risk of bias in selection of the reported result

Signalling questions	Comments	Response options
**5.1 Were the data that produced this result analysed in accordance with a pre-specified analysis plan that was finalized before unblinded outcome data were available for analysis?**	Unclear in absence of available statistical plan or protocol. However, methods section describes the statistics used to analyze the results.	NI
**Is the numerical result being assessed likely to have been selected, on the basis of the results, from...**		
**5.2. ... multiple eligible outcome measurements (e.g. scales, definitions, time points) within the outcome domain?**	It is a generally accepted and widely used parameter, resulting from simple statistics.	PN
**5.3 ... multiple eligible analyses of the data?**		PN

**Risk-of-bias judgement**		Some concerns
Optional: What is the predicted direction of bias due to selection of the reported result?		Towards null

Overall risk of bias

**Risk-of-bias judgement**		**Some concerns**
Optional: What is the overall predicted direction of bias for this outcome?		Towards null

This work is licensed under a Creative Commons Attribution-NonCommercial-NoDerivatives 4.0 International License.

Study details

**Reference**	**Smets F, Bourgois A, Vermylen C, Brichard B, Slacmuylders P, Leyman S, Sokal E. Randomised revaccination with pneumococcal polysaccharide or conjugate vaccine in asplenic children previously vaccinated with polysaccharide vaccine. Vaccine. 2007 Jul 20;25(29):5278-82. doi: 10.1016/j.vaccine.2007.05.014. Epub 2007 Jun 4. PMID: 17576024.**

Study design

X Individually-randomized parallel-group trial
☐ Cluster-randomized parallel-group trial
☐ Individually randomized cross-over (or other matched) trial

For the purposes of this assessment, the interventions being compared are defined as

Experimental: One dose of PCV7 Comparator: One dose of PVV23

Specify which outcome is being assessed for risk of bias — Pneumococcal serotype specific IgG fold rise (% ≥4 fold rise)

Specify the numerical result being assessed. In case of multiple alternative analyses being presented, specify the numeric result (e.g. RR = 1.52 (95% CI 0.83 to 2.77) and/or a reference (e.g. to a table, figure or paragraph) that uniquely defines the result being assessed.

Is the review team's aim for this result...?

☐ to assess the effect of *assignment to intervention* (the 'intention-to-treat' effect)
☐ ~~to assess the effect of *adhering to intervention* (the 'per-protocol' effect)~~

If the aim is to assess the effect of *adhering to intervention*, select the deviations from intended intervention that should be addressed (at least one must be checked):

☐ occurrence of non-protocol interventions
☐ failures in implementing the intervention that could have affected the outcome
☐ non-adherence to their assigned intervention by trial participants

Which of the following sources were <u>obtained</u> to help inform the risk-of-bias assessment? (tick as many as apply)

☐	**Journal article(s) with results of the trial**
☐	Trial protocol
☐	Statistical analysis plan (SAP)
☐	Non-commercial trial registry record (e.g. ClinicalTrials.gov record)
☐	Company-owned trial registry record (e.g. GSK Clinical Study Register record)
☐	“Grey literature” (e.g. unpublished thesis)
☐	Conference abstract(s) about the trial
☐	Regulatory document (e.g. Clinical Study Report, Drug Approval Package)
☐	Research ethics application
☐	Grant database summary (e.g. NIH RePORTER or Research Councils UK Gateway to Research)
☐	Personal communication with trialist
☐	Personal communication with the sponsor

Risk of bias assessment

Responses underlined in green are potential markers for low risk of bias, and responses in red are potential markers for a risk of bias. Where questions relate only to sign posts to other questions, no formatting is used.

Domain 1: Risk of bias arising from the randomization process

Signalling questions	Comments	Response options
**1.1 Was the allocation sequence random?**	Yes, randomization was performed with a computer based on inclusion number.	Y
**1.2 Was the allocation sequence concealed until participants were enrolled and assigned to interventions?**		Y

**1.3 Did baseline differences between intervention groups suggest a problem with the randomization process?**	No, no statistical differences are detected in the baseline characteristics except for treatment allocation (no female participants in the PPV23 group) and groups seem to be equally. This stands out but then gender was very skewed in the total population (5 females versus 14 males).	PN
**Risk-of-bias judgement**		Low
Optional: What is the predicted direction of bias arising from the randomization process?		Towards null

Domain 2: Risk of bias due to deviations from the intended interventions (*effect of assignment to intervention*)

**Signalling questions**	**Comments**	**Response options**
**2.1. Were participants aware of their assigned intervention during the trial?**	It is not described whether participants and researchers/ research personnel were blinded for treatment allocation.	NI
**2.2. Were carers and people delivering the interventions aware of participants' assigned intervention during the trial?**		NI
**2.3. If Y/PY/NI to 2.1 or 2.2: Were there deviations from the intended intervention that arose because of the trial context?**	Two participants missed blood tests and therefore were excluded. It is not known why these sampling moments were missed. It is not likely that this was induced by the trial context and due to lack of strong evidence for this, we choose PN.	PN
**2.4 If Y/PY to 2.3: Were these deviations likely to have affected the outcome?**	*N.A.*	NA
**2.5. If Y/PY/NI to 2.4: Were these deviations from intended intervention balanced between groups?**	*N.A.*	NA

**2.6 Was an appropriate analysis used to estimate the effect of assignment to intervention?**		PY
**2.7 If N/PN/NI to 2.6: Was there potential for a substantial impact (on the result) of the failure to analyse participants in the group to which they were randomized?**		NA
**Risk-of-bias judgement**		Low
Optional: What is the predicted direction of bias due to deviations from intended interventions?		Towards null

Domain 3: Missing outcome data

Signalling questions	Comments	Response options
**3.1 Were data for this outcome available for all, or nearly all, participants randomized?**	Data from 2 participants was missing, as the sample size was small 90% of data was available. It is unknown whether this made the group sizes and/or the gender distribution less equal. Therefore, we scored this as PN.	PN
**3.2 If N/PN/NI to 3.1: Is there evidence that the result was not biased by missing outcome data?**	This does not seem assessed.	PN
**3.3 If N/PN to 3.2: Could missingness in the outcome depend on its true value?**	That does not seem likely to be linked with the outcome of a vaccination. It is not stated that the 2 participants suffered from or died from pneumococcal infection.	PN
**3.4 If Y/PY/NI to 3.3: Is it likely that missingness in the outcome depended on its true value?**		PN

**Risk-of-bias judgement**		Low
Optional: What is the predicted direction of bias due to missing outcome data?		Towards null

Domain 4: Risk of bias in measurement of the outcome

**Signalling questions**	**Comments**	**Response options**
**4.1 Was the method of measuring the outcome inappropriate?**	Outcomes seem to be pre-specified and measured with internationally accepted and widely implemented assays.	PN
**4.2 Could measurement or ascertainment of the outcome have differed between intervention groups?**	Sampling timepoints and methods and measurement methods were similar for both groups.	N
**4.3 If N/PN/NI to 4.1 and 4.2: Were outcome assessors aware of the intervention received by study participants?**	Unclear, it is not described whether outcome assessors were blinded for treatment allocation.	NI
**4.4 If Y/PY/NI to 4.3: Could assessment of the outcome have been influenced by knowledge of intervention received?**	Assessment can be influenced when the assessor is not blinded for treatment allocation, as a few steps in the assay process (ELISA) can be performed differently when having this knowledge. Therefore, it is not possible to exclude.	PY
**4.5 If Y/PY/NI to 4.4: Is it likely that assessment of the outcome was influenced by knowledge of intervention received?**	It is not possible to exclude but it does not seem likely that outcomes were influenced. However, it seems more likely that assessors were blinded but this information is lacking, and therefore we cannot answer this question and we choose PN.	PN
**Risk-of-bias judgement**		Some concerns
Optional: What is the predicted direction of bias in measurement of the outcome?		Unpredictable

Domain 5: Risk of bias in selection of the reported result

Signalling questions	Comments	Response options
**5.1 Were the data that produced this result analysed in accordance with a pre-specified analysis plan that was finalized before unblinded outcome data were available for analysis?**	Unclear in absence of available statistical plan or protocol. However, methods section describes the statistics used to analyze the results.	NI
**Is the numerical result being assessed likely to have been selected, on the basis of the results, from...**		
**5.2. ... multiple eligible outcome measurements (e.g. scales, definitions, time points) within the outcome domain?**	Multiple eligible outcomes were tested (GMC rise, % above 0.2 ug/mL and % above 1.0 ug/mL and fold increase), these are all shortly described in the methods section but it is not stated which is chosen as primary outcome. All tested outcomes favor the same intervention (some are statistically significant and others not), however with a different degree. Therefore it stands out but does not seem to have influenced the final conclusion of the trial. Also, all outcomes have been fully reported.	Y
**5.3 ... multiple eligible analyses of the data?**	The same statistical test has been used.	PN
**Risk-of-bias judgement**		Some concerns
Optional: What is the predicted direction of bias due to selection of the reported result?	As the outcome has been assessed by various ways without a clear primary endpoint, bias cannot be excluded as there is no statistical analysis plan available, however all outcomes are 'known' endpoints in this field and therefore we do not assess this as high risk of bias.	Away from null

Overall risk of bias

**Risk-of-bias judgement**		**Some concerns**
Optional: What is the overall predicted direction of bias for this outcome?	Due to multiple ways of testing the outcome.	Away from null

This work is licensed under a Creative Commons Attribution-NonCommercial-NoDerivatives 4.0 International License.

Study details

**Reference**	**Orthopoulos GV, Theodoridou MC, Ladis VA, Tsousis DK, Spoulou VI. The effect of 23-valent pneumococcal polysaccharide vaccine on immunological priming induced by 7-valent conjugate vaccine in asplenic subjects with beta-thalassemia. Vaccine. 2009 Jan 14;27(3):350-4. doi: 10.1016/j.vaccine.2008.10.070. Epub 2008 Nov 14. PMID: 19010369.**

Study design

X Individually-randomized parallel-group trial
☐ Cluster-randomized parallel-group trial
☐ Individually randomized cross-over (or other matched) trial

For the purposes of this assessment, the interventions being compared are defined as

Experimental: PCV+PCV+PPV Comparator: PCV+PPV+PVV

**Specify which outcome is being assessed for risk of bias**	Pneumococcal serotype specific Geometric Mean Concentrations after PPV23-booster
**Specify the numerical result being assessed.** In case of multiple alternative analyses being presented, specify the numeric result (e.g. RR = 1.52 (95% CI 0.83 to 2.77) and/or a reference (e.g. to a table, figure or paragraph) that uniquely defines the result being assessed.	PS-specific IgG GMC in ug/mL

Is the review team's aim for this result…?

☐ to assess the effect of *assignment to intervention* (the 'intention-to-treat' effect)
☐ ~~to assess the effect of *adhering to intervention* (the 'per-protocol' effect)~~

If the aim is to assess the effect of *adhering to intervention*, select the deviations from intended intervention that should be addressed (at least one must be checked):

☐ occurrence of non-protocol interventions
☐ failures in implementing the intervention that could have affected the outcome
☐ non-adherence to their assigned intervention by trial participants

Which of the following sources were obtained to help inform the risk-of-bias assessment? (tick as many as apply)

- ☐ **Journal article(s) with results of the trial**
- ☐ Trial protocol
- ☐ Statistical analysis plan (SAP)
- ☐ Non-commercial trial registry record (e.g. ClinicalTrials.gov record)
- ☐ Company-owned trial registry record (e.g. GSK Clinical Study Register record)
- ☐ “Grey literature” (e.g. unpublished thesis)
- ☐ Conference abstract(s) about the trial
- ☐ Regulatory document (e.g. Clinical Study Report, Drug Approval Package)
- ☐ Research ethics application
- ☐ Grant database summary (e.g. NIH RePORTER or Research Councils UK Gateway to Research)
- ☐ Personal communication with trialist
- ☐ Personal communication with the sponsor

Risk of bias assessment

Responses underlined in green are potential markers for low risk of bias, and responses in red are potential markers for a risk of bias. Where questions relate only to sign posts to other questions, no formatting is used.

Domain 1: Risk of bias arising from the randomization process

Signalling questions	Comments	Response options
**1.1 Was the allocation sequence random?**	No information. Nothing stated about the randomization method.<br><br>Therefore, also no information about whether allocation sequence was concealed or not.	NI
**1.2 Was the allocation sequence concealed until participants were enrolled and assigned to interventions?**		NI

**1.3 Did baseline differences between intervention groups suggest a problem with the randomization process?**	No table with demographic characteristics has been provided. The mean age and gender distribution has been described, as well as the number of participants is both treatment allocations. It is unclear what the distribution of these few characteristics was in both treatment groups.	NI
**Risk-of-bias judgement**		Some concerns
Optional: What is the predicted direction of bias arising from the randomization process?	Important information is lacking in order to assess this domain.	Unpredictable

Domain 2: Risk of bias due to deviations from the intended interventions (*effect of assignment to intervention*)

**Signalling questions**	**Comments**	**Response options**
**2.1. Were participants aware of their assigned intervention during the trial?**	It is not stated whether participants and researchers/ research-personnel were blinded.	NI
**2.2. Were carers and people delivering the interventions aware of participants' assigned intervention during the trial?**		NI
**2.3. If Y/PY/NI to 2.1 or 2.2: Were there deviations from the intended intervention that arose because of the trial context?**	No, all participants have completed their schedule and the follow up.	PN
**2.4 If Y/PY to 2.3: Were these deviations likely to have affected the outcome?**	*N.A.*	NA
**2.5. If Y/PY/NI to 2.4: Were these deviations from intended intervention balanced between groups?**	*N.A.*	NA

**2.6 Was an appropriate analysis used to estimate the effect of assignment to intervention?**		PY
**2.7 If N/PN/NI to 2.6: Was there potential for a substantial impact (on the result) of the failure to analyse participants in the group to which they were randomized?**	*N.A.*	NA
**Risk-of-bias judgement**		Low
Optional: What is the predicted direction of bias due to deviations from intended interventions?		Towards null

Domain 3: Missing outcome data

Signalling questions	Comments	Response options
**3.1 Were data for this outcome available for all, or nearly all, participants randomized?**	All data was available.	Y
**3.2 If N/PN/NI to 3.1: Is there evidence that the result was not biased by missing outcome data?**	*N.A.*	NA
**3.3 If N/PN to 3.2: Could missingness in the outcome depend on its true value?**	*N.A.*	NA
**3.4 If Y/PY/NI to 3.3: Is it likely that missingness in the outcome depended on its true value?**		NA

**Risk-of-bias judgement**		Low
Optional: What is the predicted direction of bias due to missing outcome data?		Towards null

Domain 4: Risk of bias in measurement of the outcome

Signalling questions	Comments	Response options
**4.1 Was the method of measuring the outcome inappropriate?**	Outcomes seem to be pre-specified and according to international standards, measured by an internationally accepted assay. However, there is no consensus about which method is best for this outcome.	PN
**4.2 Could measurement or ascertainment of the outcome have differed between intervention groups?**	No, timepoints of sampling and intervention were similar between groups. The assay was also similar in both groups.	N
**4.3 If N/PN/NI to 4.1 and 4.2: Were outcome assessors aware of the intervention received by study participants?**	It is not stated that the outcome assessors were blinded.	NI
**4.4 If Y/PY/NI to 4.3: Could assessment of the outcome have been influenced by knowledge of intervention received?**	Assessment can be influenced when the assessor is not blinded for treatment allocation, as a few steps in the assay process (ELISA) can ben performed differently when having this knowledge. Therefore, it is not possible to exclude.	PY
**4.5 If Y/PY/NI to 4.4: Is it likely that assessment of the outcome was influenced by knowledge of intervention received?**	It is not possible to exclude but it does not seem likely that outcomes were influenced. However, it seems more likely that assessors were blinded but this information is lacking, and therefore we cannot answer this question and we choose PN.	PN
**Risk-of-bias judgement**		Some concerns
Optional: What is the predicted direction of bias in measurement of the outcome?		Towards null

Domain 5: Risk of bias in selection of the reported result

Signalling questions	Comments	Response options
**5.1 Were the data that produced this result analysed in accordance with a pre-specified analysis plan that was finalized before unblinded outcome data were available for analysis?**	Unclear in absence of available statistical plan or protocol. However, methods section describes the statistics used to analyze the results.	NI
**Is the numerical result being assessed likely to have been selected, on the basis of the results, from...**		
**5.2. ... multiple eligible outcome measurements (e.g. scales, definitions, time points) within the outcome domain?**	The primary outcome is clearly described and logically measured and assessed.	N
**5.3 ... multiple eligible analyses of the data?**		PN
**Risk-of-bias judgement**		Some concerns
Optional: What is the predicted direction of bias due to selection of the reported result?		Towards null

Overall risk of bias

**Risk-of-bias judgement**		**Some concerns**
Optional: What is the overall predicted direction of bias for this outcome?		Towards null

This work is licensed under a Creative Commons Attribution-NonCommercial-NoDerivatives 4.0 International License.

Study details

**Reference**	**Sari TT, Akib AAP, Gatot D, Roswita Harahap A, Bardosono S, Hadinegoro SRS. Pneumococcal vaccination for splenectomized patients with thalassemia major in Indonesia. Vaccine. 2017 Aug 16;35(35 Pt B):4583-4586. doi: 10.1016/j.vaccine.2017.07.011. Epub 2017 Jul 13. PMID: 28712490.**

Study design

X Individually-randomized parallel-group trial
☐ Cluster-randomized parallel-group trial
☐ Individually randomized cross-over (or other matched) trial

For the purposes of this assessment, the interventions being compared are defined as

Experimental: One dose of PCV13+placebo Comparator: One dose of PCV13+zinc sirup

**Specify which outcome is being assessed for risk of bias**	Anti-pneumococcal Geometric Mean Concentration
**Specify the numerical result being assessed.** In case of multiple alternative analyses being presented, specify the numeric result (e.g. RR = 1.52 (95% CI 0.83 to 2.77) and/or a reference (e.g. to a table, figure or paragraph) that uniquely defines the result being assessed.	Anti-Pn IgG GMC in ug/mL

Is the review team's aim for this result…?

☐ to assess the effect of *assignment to intervention* (the 'intention-to-treat' effect)
☐ ~~to assess the effect of *adhering to intervention* (the 'per-protocol' effect)~~

If the aim is to assess the effect of *adhering to intervention*, select the deviations from intended intervention that should be addressed (at least one must be checked):

☐ occurrence of non-protocol interventions
☐ failures in implementing the intervention that could have affected the outcome
☐ non-adherence to their assigned intervention by trial participants

Which of the following sources were <u>obtained</u> to help inform the risk-of-bias assessment? (tick as many as apply)

☐ **Journal article(s) with results of the trial**
☐ Trial protocol
☐ Statistical analysis plan (SAP)
☐ Non-commercial trial registry record (e.g. ClinicalTrials.gov record)
☐ Company-owned trial registry record (e.g. GSK Clinical Study Register record)
☐ “Grey literature” (e.g. unpublished thesis)
☐ Conference abstract(s) about the trial
☐ Regulatory document (e.g. Clinical Study Report, Drug Approval Package)
☐ Research ethics application
☐ Grant database summary (e.g. NIH RePORTER or Research Councils UK Gateway to Research)
☐ Personal communication with trialist
☐ Personal communication with the sponsor

Risk of bias assessment

Responses underlined in green are potential markers for low risk of bias, and responses in red are potential markers for a risk of bias. Where questions relate only to sign posts to other questions, no formatting is used.

Domain 1: Risk of bias arising from the randomization process

Signalling questions	Comments	Response options
**1.1 Was the allocation sequence random?**	Method of randomization is not described. Therefore it is also not evident whether allocation sequence was concealed or not.	NI
**1.2 Was the allocation sequence concealed until participants were enrolled and assigned to interventions?**		NI

**1.3 Did baseline differences between intervention groups suggest a problem with the randomization process?**	Baseline characterises are well described but no distinction has been made between the groups, therefore it is not possible to assess and answer this question.	NI
**Risk-of-bias judgement**		Some concerns
Optional: What is the predicted direction of bias arising from the randomization process?	Due to the lack of information.	Unpredictable

Domain 2: Risk of bias due to deviations from the intended interventions (*effect of assignment to intervention*)

Signalling questions	Comments	Response options
**2.1. Were participants aware of their assigned intervention during the trial?**	Participants were blinded towards their treatment allocation as the researcher used a placebo with the same shape and flavour and for the same duration of 4 weeks<br><br>It is unclear whether the researchers/research-personnel were blinded as well as this is not stated but is it possibly seen the use of the placebo. Therefore we assessed this question as probably no.	N
**2.2. Were carers and people delivering the interventions aware of participants' assigned intervention during the trial?**		PN
**2.3. If Y/PY/NI to 2.1 or 2.2: Were there deviations from the intended intervention that arose because of the trial context?**	*N.A.*	NA
**2.4 If Y/PY to 2.3: Were these deviations likely to have affected the outcome?**	*N.A.*	NA

**2.5. If Y/PY/NI to 2.4: Were these deviations from intended intervention balanced between groups?**	*N.A.*	NA
**2.6 Was an appropriate analysis used to estimate the effect of assignment to intervention?**	Participants were randomly assigned to receive zinc supplementation for 4 weeks after PCV and when receiving PPV also 4 weeks after PPV. A small number of participants was already vaccinated with PPV and therefore did not receive PPV23 after PCV13. Participant receiving PPV23 are separately analyzed from participants who do not received only PCV13. It is stated that the primary aim of this study is to investigate whether zinc supplementation improves response after PCV13+PPV23 and therefore this separation of analysis seems to be appropriate. Also, all participants are included on of the two analyses.	PY
**2.7 If N/PN/NI to 2.6: Was there potential for a substantial impact (on the result) of the failure to analyse participants in the group to which they were randomized?**	*N.A.*	NA
**Risk-of-bias judgement**		Low
Optional: What is the predicted direction of bias due to deviations from intended interventions?		Towards null

Domain 3: Missing outcome data

Signalling questions	Comments	Response options
**3.1 Were data for this outcome available for all, or nearly all, participants randomized?**	Yes	Y

**3.2 If N/PN/NI to 3.1: Is there evidence that the result was not biased by missing outcome data?**	*N.A.*	NA
**3.3 If N/PN to 3.2: Could missingness in the outcome depend on its true value?**	*N.A.*	NA
**3.4 If Y/PY/NI to 3.3: Is it likely that missingness in the outcome depended on its true value?**		NA
**Risk-of-bias judgement**		Low
Optional: What is the predicted direction of bias due to missing outcome data?		Towards null

Domain 4: Risk of bias in measurement of the outcome

**Signalling questions**	**Comments**	**Response options**
**4.1 Was the method of measuring the outcome inappropriate?**	Outcomes were pre-specified and the assay was well described.	N
**4.2 Could measurement or ascertainment of the outcome have differed between intervention groups?**	Measurement points were similar between groups, and the same assay has been used for both groups.	N
**4.3 If N/PN/NI to 4.1 and 4.2: Were outcome assessors aware of the intervention received by study participants?**	It is not described whether outcome assessors were blinded for treatment allocation.	NI

**4.4 If Y/PY/NI to 4.3: Could assessment of the outcome have been influenced by knowledge of intervention received?**	Assessment can be influenced when the assessor is not blinded for treatment allocation, as a few steps in the assay process (ELISA) can ben performed differently when having this knowledge. Therefore, it is not possible to exclude.	PY
**4.5 If Y/PY/NI to 4.4: Is it likely that assessment of the outcome was influenced by knowledge of intervention received?**	It is not possible to exclude but it does not seem likely that outcomes were influenced. However, it seems more likely that assessors were blinded but this information is lacking, and therefore we choose PN.	PN
**Risk-of-bias judgement**		Some concerns
Optional: What is the predicted direction of bias in measurement of the outcome?		Towards null

Domain 5: Risk of bias in selection of the reported result

Signalling questions	Comments	Response options
**5.1 Were the data that produced this result analysed in accordance with a pre-specified analysis plan that was finalized before unblinded outcome data were available for analysis?**	Unclear in absence of available statistical plan or protocol. However, methods section describes the statistics used to analyze the results.	NI
**Is the numerical result being assessed likely to have been selected, on the basis of the results, from...**		
**5.2. ... multiple eligible outcome measurements (e.g. scales, definitions, time points) within the outcome domain?**	The primary outcome is clearly described and logically measured and assessed using simple statistics.	N

**5.3 … multiple eligible analyses of the data?**		N
**Risk-of-bias judgement**		Some concerns
Optional: What is the predicted direction of bias due to selection of the reported result?		Towards null

Overall risk of bias

**Risk-of-bias judgement**		**Some concerns**
Optional: What is the overall predicted direction of bias for this outcome?		Towards null

This work is licensed under a Creative Commons Attribution-NonCommercial-NoDerivatives 4.0 International License.

Study details

**Reference**	**Melica G, Bartolucci P, Audureau E, Le Corvoisier P, Habibi A, Gellen J, Selmane D, Michel M, Lacabaratz C, Levy Y. Immunological Efficacy of Pneumococcal Vaccination Including the 13-Valent Pneumococcal Conjugate Vaccine in Adult Patients With Sickle Cell Disease: Results of the Randomized DREVAC Controlled Trial. Clin Infect Dis. 2023 Jun 8;76(11):1949-1958. doi: 10.1093/cid/ciad037. PMID: 36705266.**

Study design

X Individually-randomized parallel-group trial
☐ Cluster-randomized parallel-group trial
☐ Individually randomized cross-over (or other matched) trial

For the purposes of this assessment, the interventions being compared are defined as

Experimental: PCV13+PPV23 Comparator: PPV23

**Specify which outcome is being assessed for risk of bias**	PS-specific IgG geometric mean concentration
**Specify the numerical result being assessed.** In case of multiple alternative analyses being presented, specify the numeric result (e.g. RR = 1.52 (95% CI 0.83 to 2.77) and/or a reference (e.g. to a table, figure or paragraph) that uniquely defines the result being assessed.	Levels

Is the review team's aim for this result…?

☐ to assess the effect of *assignment to intervention* (the 'intention-to-treat' effect)
☐ ~~to assess the effect of *adhering to intervention* (the 'per-protocol' effect)~~

If the aim is to assess the effect of *adhering to intervention*, select the deviations from intended intervention that should be addressed (at least one must be checked):

☐ occurrence of non-protocol interventions
☐ failures in implementing the intervention that could have affected the outcome
☐ non-adherence to their assigned intervention by trial participants

Which of the following sources were <u>obtained</u> to help inform the risk-of-bias assessment? (tick as many as apply)

☐	**Journal article(s) with results of the trial**
☐	Trial protocol
☐	Statistical analysis plan (SAP)
☐	Non-commercial trial registry record (e.g. ClinicalTrials.gov record)
☐	Company-owned trial registry record (e.g. GSK Clinical Study Register record)
☐	“Grey literature” (e.g. unpublished thesis)
☐	Conference abstract(s) about the trial
☐	Regulatory document (e.g. Clinical Study Report, Drug Approval Package)
☐	Research ethics application
☐	Grant database summary (e.g. NIH RePORTER or Research Councils UK Gateway to Research)
☐	Personal communication with trialist
☐	Personal communication with the sponsor

Risk of bias assessment

Responses underlined in green are potential markers for low risk of bias, and responses in red are potential markers for a risk of bias. Where questions relate only to sign posts to other questions, no formatting is used.

Domain 1: Risk of bias arising from the randomization process

Signalling questions	Comments	Response options
**1.1 Was the allocation sequence random?**	It is described that randomization was centralized and stratified by the use of hydroxyurea, therefore these questions are scores as PY.	PY
**1.2 Was the allocation sequence concealed until participants were enrolled and assigned to interventions?**		PY

**1.3 Did baseline differences between intervention groups suggest a problem with the randomization process?**	Demographic baseline characteristics are described per treatment group and seem logically distributed, except for the comorbidity of hypertension. However, no statistical tests have been performed	PN
**Risk-of-bias judgement**		Low
Optional: What is the predicted direction of bias arising from the randomization process?		Towards null

Domain 2: Risk of bias due to deviations from the intended interventions (*effect of assignment to intervention*)

**Signalling questions**	**Comments**	**Response options**
**2.1. Were participants aware of their assigned intervention during the trial?**	It is not described that participants and researchers/research-personnel were blinded for their treatment allocation and, since the number of vaccinations was not equal in both groups, it is not described that placebo was used.	PY
**2.2. Were carers and people delivering the interventions aware of participants' assigned intervention during the trial?**		PY
**2.3. If Y/PY/NI to 2.1 or 2.2: Were there deviations from the intended intervention that arose because of the trial context?**	Unclear, it seems that the per-protocol population was 118 (PCV13+PPV23 group=57 and PPV23=61) and the intention to treat population 128 (PCV13+PPV23=63 and PPV23=65). However, it is not described how this different has been arisen and what the characteristics were of these participants.	NI
**2.4 If Y/PY to 2.3: Were these deviations likely to have affected the outcome?**	For the primary outcome, a mITT analysis has been performed and in both groups deviations have occurred. Therefore it does not seem likely that these deviations have affected the primary outcome.	PN

**2.5. If Y/PY/NI to 2.4: Were these deviations from intended intervention balanced between groups?**	In the pCV13+PPV23 group 6 were lost and in the PPV23 group 4. This seems sufficiently balanced. However, the deviations were not described and therefore it is unclear to what extend these deviations proceeded. Important information is lacking to answer this question.	NI
**2.6 Was an appropriate analysis used to estimate the effect of assignment to intervention?**	For the primary outcome, a mITT analysis was used which is considered appropriate. However, for all the other analyses it seems that a per-protocol analysis has been conducted	PY
**2.7 If N/PN/NI to 2.6: Was there potential for a substantial impact (on the result) of the failure to analyse participants in the group to which they were randomized?**	*N.A.*	NA
**Risk-of-bias judgement**		Some concerns
Optional: What is the predicted direction of bias due to deviations from intended interventions?		Away from null

Domain 3: Missing outcome data

**Signalling questions**	**Comments**	**Response options**
**3.1 Were data for this outcome available for all, or nearly all, participants randomized?**	For the primary outcome, 92% of participants had available data for the mITT analysis.	PN
**3.2 If N/PN/NI to 3.1: Is there evidence that the result was not biased by missing outcome data?**		PN
**3.3 If N/PN to 3.2: Could missingness in the outcome depend on its true value?**	Not described thus unclear.	NI

**3.4 If Y/PY/NI to 3.3: Is it likely that missingness in the outcome depended on its true value?**	It is not likely that the missingness in the outcome is depended on its true value as the outcome is % of adequate response towards a certain amount of serotypes and it is not described that these participants were lost to follow up due to pneumococcal infection.	PN
**Risk-of-bias judgement**		Some concerns
Optional: What is the predicted direction of bias due to missing outcome data?		Away from null

Domain 4: Risk of bias in measurement of the outcome

**Signalling questions**	**Comments**	**Response options**
**4.1 Was the method of measuring the outcome inappropriate?**		PN
**4.2 Could measurement or ascertainment of the outcome have differed between intervention groups?**	This was similar for groups.	N
**4.3 If N/PN/NI to 4.1 and 4.2: Were outcome assessors aware of the intervention received by study participants?**	It is described that outcome assessors were blinded.	N
**4.4 If Y/PY/NI to 4.3: Could assessment of the outcome have been influenced by knowledge of intervention received?**	*N.A.*	NA
**4.5 If Y/PY/NI to 4.4: Is it likely that assessment of the outcome was influenced by knowledge of intervention received?**		NA

**Risk-of-bias judgement**		Low
Optional: What is the predicted direction of bias in measurement of the outcome?		Towards null

Domain 5: Risk of bias in selection of the reported result

Signalling questions	Comments	Response options
**5.1 Were the data that produced this result analysed in accordance with a pre-specified analysis plan that was finalized before unblinded outcome data were available for analysis?**	The protocol and statistical plan are not available. The method section describes the statistics used and states that subgroup analyses were prespecified. Many different analyses have been conducted, some with the mITT population and some with the PP population.	NI
**Is the numerical result being assessed likely to have been selected, on the basis of the results, from...**		
**5.2. ... multiple eligible outcome measurements (e.g. scales, definitions, time points) within the outcome domain?**	The primary endpoint is stated in the method section. However, with this amount of measurements for the same outcome and the absence of the protocol/SAP available to analyse, it is not possible to excluded whether multiple analyses have been performed before choosing the primary outcome. Also, outcomes are difficult to evaluate simultaneously as some have been assed with the mITT population and some with the PP population. We lack information to excluded this but also there is no evidence that the primary outcome was picked afterwards.	NI
**5.3 ... multiple eligible analyses of the data?**	It is unclear whether outcomes have been selected afterwards as only for the primary outcome the mITT has been performed and for the other outcomes only the PP population. Although it does not seem likely that data was handled inappropriately but important information is lacking.	NI
**Risk-of-bias judgement**		Some concerns
Optional: What is the predicted direction of bias due to selection of the reported result?		Away from null

Overall risk of bias

**Risk-of-bias judgement**		**Some concerns**
Optional: What is the overall predicted direction of bias for this outcome?		Away from null

This work is licensed under a Creative Commons Attribution-NonCommercial-NoDerivatives 4.0 International License.

Study details

**Reference**	**Quinn CT, Wiedmann RT, Jarovsky D, Lopez-Medina E, Rodriguez HM, Papa M, Boggio G, Shou Q, Dagan R, Richmond P, Feemster K, McFetridge R, Tamms G, Lupinacci R, Musey L, Bickham K. Safety and immunogenicity of V114, a 15-valent pneumococcal conjugate vaccine, in children with SCD: a V114-023 (PNEU-SICKLE) study. Blood Adv. 2023 Feb 14;7(3):414-421. doi:**

Study design

X Individually-randomized parallel-group trial
☐ Cluster-randomized parallel-group trial
☐ Individually randomized cross-over (or other matched) trial

For the purposes of this assessment, the interventions being compared are defined as

Experimental: One dose of PCV15 Comparator: One dose of PCV13

Specify which outcome is being assessed for risk of bias — Pneumococcal specific geometric mean concentration

Specify the numerical result being assessed. In case of multiple alternative analyses being presented, specify the numeric result (e.g. RR = 1.52 (95% CI 0.83 to 2.77) and/or a reference (e.g. to a table, figure or paragraph) that uniquely defines the result being assessed. — Pneumococcal specific IgG GMCs in ug/mL.

Is the review team's aim for this result…?

☐ to assess the effect of *assignment to intervention* (the 'intention-to-treat' effect)
☐ ~~to assess the effect of *adhering to intervention* (the 'per-protocol' effect)~~

If the aim is to assess the effect of *adhering to intervention*, select the deviations from intended intervention that should be addressed (at least one must be checked):

☐ occurrence of non-protocol interventions
☐ failures in implementing the intervention that could have affected the outcome
☐ non-adherence to their assigned intervention by trial participants

Which of the following sources were <u>obtained</u> to help inform the risk-of-bias assessment? (tick as many as apply)

☐	**Journal article(s) with results of the trial**
☐	Trial protocol
☐	Statistical analysis plan (SAP)
☐	Non-commercial trial registry record (e.g. ClinicalTrials.gov record)
☐	Company-owned trial registry record (e.g. GSK Clinical Study Register record)
☐	"Grey literature" (e.g. unpublished thesis)
☐	Conference abstract(s) about the trial
☐	Regulatory document (e.g. Clinical Study Report, Drug Approval Package)
☐	Research ethics application
☐	Grant database summary (e.g. NIH RePORTER or Research Councils UK Gateway to Research)
☐	Personal communication with trialist
☐	Personal communication with the sponsor

Risk of bias assessment

Responses underlined in green are potential markers for low risk of bias, and responses in red are potential markers for a risk of bias. Where questions relate only to sign posts to other questions, no formatting is used.

Domain 1: Risk of bias arising from the randomization process

Signalling questions	Comments	Response options
**1.1 Was the allocation sequence random?**	Randomization was performed using an interactive response technology system.	Y
**1.2 Was the allocation sequence concealed until participants were enrolled and assigned to interventions?**		Y

**1.3 Did baseline differences between intervention groups suggest a problem with the randomization process?**	Baseline characteristics are displayed per group and in total.	N
**Risk-of-bias judgement**		Low
Optional: What is the predicted direction of bias arising from the randomization process?		Towards null

Domain 2: Risk of bias due to deviations from the intended interventions (*effect of assignment to intervention*)

**Signalling questions**	**Comments**	**Response options**
**2.1. Were participants aware of their assigned intervention during the trial?**	The study was double blinded for participants (their guardians) and for the researchers who assessed endpoints.	N
**2.2. Were carers and people delivering the interventions aware of participants' assigned intervention during the trial?**		N
**2.3. If Y/PY/NI to 2.1 or 2.2: Were there deviations from the intended intervention that arose because of the trial context?**	*N.A.*	NA
**2.4 If Y/PY to 2.3: Were these deviations likely to have affected the outcome?**	*N.A.*	NA
**2.5. If Y/PY/NI to 2.4: Were these deviations from intended intervention balanced between groups?**	*N.A.*	NA

**2.6 Was an appropriate analysis used to estimate the effect of assignment to intervention?**		Y
**2.7 If N/PN/NI to 2.6: Was there potential for a substantial impact (on the result) of the failure to analyse participants in the group to which they were randomized?**	*N.A.*	NA
**Risk-of-bias judgement**		Low
Optional: What is the predicted direction of bias due to deviations from intended interventions?		Towards null

Domain 3: Missing outcome data

Signalling questions	Comments	Response options
**3.1 Were data for this outcome available for all, or nearly all, participants randomized?**	2 patients were lost to follow up, this comprises approximately 2% of all participants.	Y
**3.2 If N/PN/NI to 3.1: Is there evidence that the result was not biased by missing outcome data?**	*N.A.*	NA
**3.3 If N/PN to 3.2: Could missingness in the outcome depend on its true value?**	*N.A.*	NA
**3.4 If Y/PY/NI to 3.3: Is it likely that missingness in the outcome depended on its true value?**		NA

**Risk-of-bias judgement**		Low
Optional: What is the predicted direction of bias due to missing outcome data?		Towards null

Domain 4: Risk of bias in measurement of the outcome

**Signalling questions**	**Comments**	**Response options**
**4.1 Was the method of measuring the outcome inappropriate?**	The assay measuring the primary endpoint is a less often used method developed by the same pharmaceutical company as the intervention (PCV15) that is assessed as the experimental intervention in this trial. However, the method has been described in detail in 2 articles whereto a reference has been made by the authors. Therefore, this rises no concerns. The serotype specific GMCs are a regular outcome when assessing PCVs.	PN
**4.2 Could measurement or ascertainment of the outcome have differed between intervention groups?**	Measurement timepoints and the used assay was similar for both groups.	N
**4.3 If N/PN/NI to 4.1 and 4.2: Were outcome assessors aware of the intervention received by study participants?**	Outcome assessors we described to be blinded to treatment allocation.	N
**4.4 If Y/PY/NI to 4.3: Could assessment of the outcome have been influenced by knowledge of intervention received?**	*N.A.*	NA
**4.5 If Y/PY/NI to 4.4: Is it likely that assessment of the outcome was influenced by knowledge of intervention received?**		NA
**Risk-of-bias judgement**		Low

Optional: What is the predicted direction of bias in measurement of the outcome?		Towards null

Domain 5: Risk of bias in selection of the reported result

**Signalling questions**	**Comments**	**Response options**
**5.1 Were the data that produced this result analysed in accordance with a pre-specified analysis plan that was finalized before unblinded outcome data were available for analysis?**	The protocol and statistical plan are not available. However, It is stated on the website of the publisher that access to the full study data can be requested. Also, the article describes the used statistical tests in the method section and declares to have been performing an ad hoc test accompanied by description. Therefore this questions has been scored with PY.	PY
**Is the numerical result being assessed likely to have been selected, on the basis of the results, from...**		
**5.2. ... multiple eligible outcome measurements (e.g. scales, definitions, time points) within the outcome domain?**	Outcome is logical and results from simple statistics which are described in the methods section.	N
**5.3 ... multiple eligible analyses of the data?**		N
**Risk-of-bias judgement**		Low
Optional: What is the predicted direction of bias due to selection of the reported result?		Towards null

Overall risk of bias

**Risk-of-bias judgement**		**Low**
Optional: What is the overall predicted direction of bias for this outcome?		Towards null

This work is licensed under a Creative Commons Attribution-NonCommercial-NoDerivatives 4.0 International License.

Revised Cochrane risk-of-bias tool for randomized crossover trials
TEMPLATE FOR COMPLETION

Version of 18 March 2021

The development of the RoB 2 tool was supported by the MRC Network of Hubs for Trials Methodology Research (MR/L004933/2- N61), with the support of the host MRC ConDuCT-II Hub (Collaboration and innovation for Difficult and Complex randomised controlled Trials In Invasive procedures - MR/K025643/1), by MRC research grant MR/M025209/1, and by a grant from The Cochrane Collaboration.

Supplementary materials: *The immunogenicity of pneumococcal conjugate vaccinations in patients with asplenia and hyposplenia: a systematic review*

This work is licensed under a Creative Commons Attribution-NonCommercial-NoDerivatives 4.0 International License.

Study details

**Reference**	**Rezai MS, Ghaffari J, Mahdavi M, Bahari A, Ala S. Conjugate and 23-valent pneumococcal polysaccharide booster vaccination in asplenic patients with thalassemia major: A randomized clinical trial study. Caspian J Intern Med. 2017 Winter;8(1):16-22. PMID: 28503278; PMCID: PMC5412244.**

Study design

☐ Individually-randomized parallel-group trial
☐ Cluster-randomized parallel-group trial
X Individually randomized cross-over (or other matched) trial

For the purposes of this assessment, the interventions being compared are defined as

Experimental: PPV23 followed by PCV13 Comparator: PCV13 followed by PPV23

**Specify which outcome is being assessed for risk of bias**	Anti-pneumococcal IgG levels
**Specify the numerical result being assessed.** In case of multiple alternative analyses being presented, specify the numeric result (e.g. RR = 1.52 (95% CI 0.83 to 2.77) and/or a reference (e.g. to a table, figure or paragraph) that uniquely defines the result being assessed.	

Is the review team's aim for this result…?

☐ to assess the effect of *assignment to intervention* (the 'intention-to-treat' effect)
☐ ~~to assess the effect of *adhering to intervention* (the 'per-protocol' effect)~~

If the aim is to assess the effect of *adhering to intervention*, select the deviations from intended intervention that should be addressed (at least one must be checked):

☐ occurrence of non-protocol interventions
☐ failures in implementing the intervention that could have affected the outcome
☐ non-adherence to their assigned intervention by trial participants

Which of the following sources were <u>obtained</u> to help inform the risk-of-bias assessment? (tick as many as apply)

- ☐ **Journal article(s) with results of the trial**
- ☐ Trial protocol
- ☐ Statistical analysis plan (SAP)
- ☐ Non-commercial trial registry record (e.g. ClinicalTrials.gov record)
- ☐ Company-owned trial registry record (e.g. GSK Clinical Study Register record)
- ☐ "Grey literature" (e.g. unpublished thesis)
- ☐ Conference abstract(s) about the trial
- ☐ Regulatory document (e.g. Clinical Study Report, Drug Approval Package)
- ☐ Research ethics application
- ☐ Grant database summary (e.g. NIH RePORTER or Research Councils UK Gateway to Research)
- ☐ Personal communication with trialist
- ☐ Personal communication with the sponsor

Risk of bias assessment

Responses underlined in green are potential markers for low risk of bias, and responses in red are potential markers for a risk of bias. Where questions relate only to sign posts to other questions, no formatting is used.

Domain 1a: Risk of bias arising from the randomization process

Signalling questions	Comments	Response options
**1.1 Was the allocation sequence random?**	Unclear, method of randomization is not described in detail. (The eligible patients were divided into two groups (A and B) based on sequential and random.)<br><br>Therefore, second question cannot be answered as well.	NI
**1.2 Was the allocation sequence concealed until participants were enrolled and assigned to interventions?**		NI

**1.3 Did baseline differences between intervention groups at the start of the first period suggest a problem with the randomization process?**	Baseline characteristics are not displayed in a table. The distribution of gender and age are described. Therefore we do not have enough information to answer this question.	NI
**Risk-of-bias judgement**		Some concerns
Optional: What is the predicted direction of bias arising from the randomization process?		Unpredictable

Domain S: Risk of bias arising from period and carryover effects

Signalling questions	Comments	Response options
**S.1 Was the number of participants allocated to each of the two sequences equal or nearly equal?**	Yes, 1:1.	Y
**S.2 If N/PN/NI to S.1: Were period effects accounted for in the analysis?**	*N.A.*	NA
**S.3 Was there sufficient time for any carryover effects to have disappeared before outcome assessment in the second period?**	No, vaccine-induced immunity cannot wash out in 8 weeks. Therefore, I question the classification of this RCT as a cross-over trial instead of a regular RCT.	N

**Risk-of-bias judgement**		High
Optional: What is the predicted direction of bias arising from period and carryover effects?		NA

Domain 2: Risk of bias due to deviations from the intended interventions (effect of assignment to intervention)

Signalling questions	Comments	Response options
**2.1. Were participants aware of their assigned intervention during each period of the trial?**	It is stated that the trial was double blind.	N
**2.2. Were carers and people delivering the interventions aware of participants' assigned intervention during each period of the trial?**		N
**2.3. If Y/PY/NI to 2.1 or 2.2: Were there deviations from the intended intervention that arose because of the trial context?**	*N.A.*	NA
**2.4 If Y/PY to 2.3: Were these deviations likely to have affected the outcome?**	*N.A.*	NA
**2.5. If Y/PY/NI to 2.4: Were these deviations from intended intervention balanced between groups?**	*N.A.*	NA
**2.6 Was an appropriate analysis used to estimate the effect of assignment to intervention?**	It seems that participants were analyzed in their original group and that beside some loss to follow up no deviations appeard.	PY

**2.7 If N/PN/NI to 2.6: Was there potential for a substantial impact (on the result) of the failure to analyse participants in the group to which they were randomized?**	*N.A.*	NA
**Risk-of-bias judgement**		Low
Optional: What is the predicted direction of bias due to deviations from intended interventions?		Towards null

Domain 3: Risk of bias due to missing outcome data

**Signalling questions**	**Comments**	**Response options**
**3.1 Were data for this outcome available for all, or nearly all, participants randomized?**	50 participants were randomized and 43 completed the study. For a continuous outcome and a small sample size, this seems to be less then nearly all. Fortunately, loss to follow up is almost equal in both groups.	PN
**3.2 If N/PN/NI to 3.1: Is there evidence that the result was not biased by missing outcome data?**	No analysis has been performed.	N
**3.3 If N/PN to 3.2 Could missingness in the outcome depend on its true value?**	It could but has not been described.<br><br>It is not likely that the missingness depends on the true value, it has not been described that these participants were lost to follow up or died because of pneumococcal disease. Therefore, this question is scored by PN.	NI
**3.4 If Y/PY/NI to 3.3: Is it likely that missingness in the outcome depended on its true value?**		PN
**Risk-of-bias judgement**		Some concerns
Optional: What is the predicted direction of bias due to missing outcome data?		Towards null

Domain 4: Risk of bias in measurement of the outcome

Signalling questions	Comments	Response options
**4.1 Was the method of measuring the outcome inappropriate?**	Outcomes are pre-specified and logical. The measuring method is adequately referred to and customary for this endpoint.	N
**4.2 Could measurement or ascertainment of the outcome have differed between interventions within each sequence?**	Measurement timepoints and assay were similar for both groups	N
**4.3 If N/PN/NI to 4.1 and 4.2: Were outcome assessors aware of the intervention received by study participants?**	It is described that the study was double blinded, however it is unclear whether assessors performing the ELISA were also blinded but this seems likely. Therefore we choose PN.	PN
**4.4 If Y/PY/NI to 4.3: Could assessment of the outcome have been influenced by knowledge of intervention received?**	*N.A.*	NA
**4.5 If Y/PY/NI to 4.4: Is it likely that assessment of the outcome was influenced by knowledge of intervention received?**		NA
**Risk-of-bias judgement**		Low
Optional: What is the predicted direction of bias in measurement of the outcome?		Towards null

Domain 5: Risk of bias in selection of the reported result

Signalling questions	Comments	Response options

**5.1 Were the data that produced this result analysed in accordance with a pre-specified analysis plan that was finalized before unblinded outcome data were available for analysis?**	Uncertain as the protocol and statistical plan are not available. In the method section the type of statistical test are shortly described, but it is not described which test has been used for which outcome. Information is lacking to adequately answer this question.	NI
**Is the numerical result being assessed likely to have been selected, on the basis of the results, from...**		
**5.2. ... multiple eligible outcome measurements (e.g. scales, definitions, time points) within the outcome domain?**	It is not likely as the result is a simple outcome resulting from simple statistics.	PN
**5.3 ... multiple eligible analyses of the data?**		PN
**5.4 Is a result based on data from both periods sought, but unavailable on the basis of carryover having been identified?**	Data from both periods contribute to the endpoint.	N
**Risk-of-bias judgement**		Some concerns
Optional: What is the predicted direction of bias due to selection of the reported result?		Towards null

Overall risk of bias

**Risk-of-bias judgement**	High risk due to domain S. However, I question whether the classification of this trial is correct, when analysed with the Rob2 tool for individually clustered randomized parallel group trials, the risk of bias of this trial will be lower.	**High**
Optional: What is the overall predicted direction of bias for this outcome?		Unpredictable

This work is licensed under a Creative Commons Attribution-NonCommercial-NoDerivatives 4.0 International License.

3. Assessment of the Risk of Bias for nonrandomized (comparative and noncomparative trials) using the Newcastle-Ottawa scale for cohort studies

**Table 1. Supplementary Materials: Newcastle Ottawa risk of bias for cohort studies per domain**			
**Selection**			
**Representativeness exposed cohort**	**Selection non-exposed cohort**	**Ascertainment of exposure**	**Outcome not present at start study**
**Scored with max one star**<br><br>*We considered a sample size <30 patients as insufficient to allow generalization of the results (we based this on sample size calculations of our trials) and this will be scored with 0 stars. Sample sizes of 30-40 without additional factors that could compromise generalizability can be scored with one star.*<br><br>*<u>Other factors</u> that were used in this assessment:*<br>*-patients should be representative of according asplenic subpopulation*<br>*-there should be no concomitant comorbidities that compromise generalization such as additional immunocompromise*<br>*-there should be definitive asplenia (splenectomy) or hyposplenia (patients with SCD or patients with objectified reduced splenic function through measurement of pitted red cell count >4%, demonstration of Howell Jolly Bodies, or through scintigraphy*<br>*-age should not substantially range, as this can affect vaccine response and thereby contribute to heterogeneity*	**Scored with max one star**<br><br>*In case there was a control group with eusplenic or unvaccinated controls. This control group should have a size of at least 15% of patient sample size.*	**Scored with max one star**<br><br>*In case vaccination was performed during the study, or when vaccination was adequately and reliably registered.*	**Scored with max one star**<br><br>*In case baseline measurement of the investigated outcome was performed.*
**Comparability**			

**Comparability of cohorts through design or analysis**	**Can be score with max 2 stars**<br><br>*In case cohorts were compared and analyses were adjusted for underlying comorbidity when the group was mixed, adjusted for age, adjusted for other immunocompromising conditions when group was mixed.*	
**Outcome**		
**Assessment outcome**	**Follow up duration sufficient**	**Adequacy of follow up**
**Score with max one star**<br><br>*In case outcomes were predefined (at least in the method section and preferably in the protocol or statistical plan if available) and clearly described, when the immunogenicity endpoint chosen is usual (such as the WHO or AAAA thresholds, the GMC height, fold change, etc.), when the assay is sufficiently described, and when the outcome does not seem to be the result of multiple assays/tests that were not predefined.*	**Scored with max one star**<br><br>*In case assessment of primary response was performed at least 3 weeks after vaccination or primary series and/or booster response was assessed at least 1 week after vaccination. In case of follow up studies, when follow up duration was equal for all participants.*	**Scored with max one star**<br><br>*When loss to follow up was <5%.*
**Final assessment: poor (0 – 2 points), fair (3 – 5 points), or good quality (6 – 9 points)**		

<table>
<tr><td colspan="4">Study: Nowak-Wegrzyn A, Winkelstein JA, Swift AJ, Lederman HM. Serum opsonic activity in infants with sickle-cell disease immunized with pneumococcal polysaccharide protein conjugate vaccine. The Pneumococcal Conjugate Vaccine Study Group. Clin Diagn Lab Immunol. 2000 Sep;7(5):788-93. doi: 10.1128/CDLI.7.5.788-793.2000. PMID: 10973456; PMCID: PMC95957.</td></tr>
<tr><td colspan="4">Selection</td></tr>
<tr><td>Representativeness exposed cohort</td><td>Selection non-exposed cohort</td><td>Ascertainment of exposure</td><td>Outcome not present at start study</td></tr>
<tr><td>Scored with max one star
Scored with: 0 stars

Sample size was 38 and homogeneous regarding (absence of) other comorbidities and age range.</td><td>Scored with max one star
Scored with: 1 star

10 (26%) age matched African-American healthy controls were included which were also vaccinated.</td><td>Scored with max one star
Scored with: 1 star

Vaccination was administered during the trial.</td><td>Scored with max one star
Scored with: 1 star

Baseline measurements were performed.</td></tr>
<tr><td colspan="4">Comparability</td></tr>
<tr><td colspan="2">Comparability of cohorts through design or analysis</td><td colspan="2">Can be score with max 2 stars
Scored with: 1 star

Cohorts were compared but not adjusted for other factors.</td></tr>
<tr><td colspan="4">Outcome</td></tr>
<tr><td colspan="2">Assessment outcome</td><td>Follow up duration sufficient</td><td>Adequacy of follow up</td></tr>
<tr><td colspan="2">Score with max one star
Scored with: 1 star

Outcomes seem to be pre-defined and are clearly described. The assays used for outcome assessment are clearly described in detail. The chosen endpoint value is not very usual as opsonic activity is mostly displayed in geometric mean titers rather than %, however the assay has been described in detail and therefore this is unlikely to increase the risk of bias. It is not described whether assessment was blinded.</td><td>Scored with max one star
Scored with: 1 star

Is sufficient for initial response.</td><td>Scored with max one star
Scored with: 0 stars

Unclear as it is not described whether all participants completed the trial with all interventions and sampling moments.</td></tr>
<tr><td colspan="4">Final assessment: 6/9 stars (GOOD)</td></tr>
</table>

Study: *O'Brien KL, Swift AJ, Winkelstein JA, Santosham M, Stover B, Luddy R, Gootenberg JE, Nold JT, Eskenazi A, Snader SJ, Lederman HM. Safety and immunogenicity of heptavalent pneumococcal vaccine conjugated to CRM(197) among infants with sickle cell disease. Pneumococcal Conjugate Vaccine Study Group. Pediatrics. 2000 Nov;106(5):965-72. doi: 10.1542/peds.106.5.965. PMID: 11061761.*

Selection			
**Representativeness exposed cohort**	**Selection non-exposed cohort**	**Ascertainment of exposure**	**Outcome not present at start study**
**Scored with max one star**<br>Scored with: **1 star**<br><br>*Sample size was 34 small and homogeneous regarding (absence of) other comorbidities and age was not range.*	**Scored with max one star**<br>Scored with: **1 star**<br><br>*11 (32%) age matched African-American healthy controls were included which were also vaccinated.*	**Scored with max one star**<br>Scored with: **1 star**<br><br>*Vaccination was performed during the study.*	**Scored with max one star**<br>Scored with: **1 star**<br><br>*Baseline measurements were performed in this study.*
**Comparability**			
**Comparability of cohorts through design or analysis**		**Can be score with max 2 stars**<br>Scored with: **1 star**<br><br>*Cohorts were compared but not adjusted for other factors.*	
**Outcome**			
**Assessment outcome**		**Follow up duration sufficient**	**Adequacy of follow up**
**Score with max one star**<br>Scored with: **1 star**<br><br>No protocol or statistical plan are available but outcomes seem to be pre-defined and are usual. The chosen assay is well described and well known for this endpoint		**Scored with max one star**<br>Scored with: **1 star**<br><br>*Adequate for initial peak response.*	**Scored with max one star**<br>Scored with: **0 stars**<br><br>*Loss to follow up for the entire trial population was 19.1 % (9/47).*
**Final assessment: 7/9 stars (GOOD)**			

<table>
<tr><td colspan="4">Study: Stoehr GA, Rose MA, Eber SW, Heidemann K, Schubert R, Zielen S. Immunogenicity of sequential pneumococcal vaccination in subjects splenectomised for hereditary spherocytosis. Br J Haematol. 2006 Mar;132(6):788-90. doi: 10.1111/j.1365-2141.2005.05918.x. PMID: 16487181.</td></tr>
<tr><td colspan="4">Selection</td></tr>
<tr><td>Representativeness exposed cohort</td><td>Selection non-exposed cohort</td><td>Ascertainment of exposure</td><td>Outcome not present at start study</td></tr>
<tr><td>Scored with max one star
Scored with: 1 star

The trial population comprised patients with hereditary spherocytosis and complete or partial splenectomy, with an objectified loss of splenic function (through a Tc-whole Body scan). The age ranged from ca 4 – 22 years of age which seems adequate. Sample size was 39.</td><td>Scored with max one star
Scored with: 0 stars

A very small control group comprising healthy but splenectomised controls was included. This group comprised of only 3 (7%) participants and can therefore not contribute to decreasing the risk of bias.</td><td>Scored with max one star
Scored with: 1 star

Vaccination was performed during the study.</td><td>Scored with max one star
Scored with: 1 star

Baseline measurements have been performed at the beginning of the study.</td></tr>
<tr><td colspan="4">Comparability</td></tr>
<tr><td colspan="2">Comparability of cohorts through design or analysis</td><td colspan="2">Can be score with max 2 stars
Scored with: 0 stars

Not performed.</td></tr>
<tr><td colspan="4">Outcome</td></tr>
<tr><td colspan="2">Assessment outcome</td><td>Follow up duration sufficient</td><td>Adequacy of follow up</td></tr>
<tr><td colspan="2">Score with max one star
Scored with: 1 star

No protocol or statistical analysis plan were available. Outcomes are described in the method section, but not very extensively. However, the assay has been described in a related article where to a reference was made.</td><td>Scored with max one star
Scored with: 1 star

Sufficient</td><td>Scored with max one star
Scored with: 1 star

It is described that all participants completed the measurements.</td></tr>
<tr><td colspan="4">Final assessment: 6/9 stars (GOOD)</td></tr>
</table>

**Study:** *Reinert P, Benkerrou M, de Montalembert M, Lesprit E, Abadie I, Bernaudin F, Doit C, Bingen E, Tetelboum R, Bonnet E. Immunogenicity and safety of a pneumococcal conjugate 7-valent vaccine in infants with sickle cell disease. Pediatr Infect Dis J. 2007 Dec;26(12):1105-9. doi: 10.1097/INF.0b013e31814614c6. PMID: 18043446.*			
**Selection**			
**Representativeness exposed cohort**	**Selection non-exposed cohort**	**Ascertainment of exposure**	**Outcome not present at start study**
**Scored with max one star**<br>Scored with: **1 star**<br><br>*The trial population comprised 51 infants of about 2 months of age with sickle cell disease and was homogeneous regarding (absence of) other comorbidities and age range.*	**Scored with max one star**<br>Scored with: **0 stars**<br><br>*No control group was included.*	**Scored with max one star**<br>Scored with: **1 star**<br><br>*Vaccination was administered during the study.*	**Scored with max one star**<br>Scored with: **1 star**<br><br>*Baseline measurements were performed.*
**Comparability**			
**Comparability of cohorts through design or analysis**		**Can be score with max 2 stars**<br>Scored with: **0 stars**<br><br>*No cohorts were compared and no adjustments were made.*	
**Outcome**			
**Assessment outcome**		**Follow up duration sufficient**	**Adequacy of follow up**
**Score with max one star**<br>Scored with: **1 star**<br><br>*No protocol or statistical plan available. Outcomes seem predefined, are usual for assessment of immunogenicity and are described in the methods section. Additionally, performed assays have been described.*		**Scored with max one star**<br>Scored with: **1 star**<br><br>*Follow up duration is usual for this endpoint.*	**Scored with max one star**<br>Scored with: **0 stars**<br><br>*For the primary endpoint, data was available from 45/51 participants, with means a loss of 12% which exceeds the predefined 5%.*
**Final assessment: 5/9 stars (FAIR)**			

<table>
<tr><td colspan="4">Study: Mikoluc B, Kayhty H, Bernatowska E, Motkowski R. Immune response to the 7-valent pneumococcal conjugate vaccine in 30 asplenic children. Eur J Clin Microbiol Infect Dis. 2008 Oct;27(10):923-8. doi: 10.1007/s10096-008-0523-5. Epub 2008 Jun 27. PMID: 18584224.</td></tr>
<tr><td colspan="4">Selection</td></tr>
<tr><td>Representativeness exposed cohort</td><td>Selection non-exposed cohort</td><td>Ascertainment of exposure</td><td>Outcome not present at start study</td></tr>
<tr><td>Scored with max one star
Scored with: 0 stars

The trial population comprised patients with congenital asplenia or asplenia due to splenectomy for various underlying benign conditions without additional immunocompromise or other factors likely to influence the vaccine response. However, the sample size was small (30) and the age ranged substantially → children of 4 months of age may respond different compared to young adults of 19 years of age.</td><td>Scored with max one star
Scored with: 0 stars

No control group was included.</td><td>Scored with max one star
Scored with: 1 star

Vaccination was performed during the study.</td><td>Scored with max one star
Scored with: 1 star

Baseline measurement were performed during the study.</td></tr>
<tr><td colspan="4">Comparability</td></tr>
<tr><td colspan="2">Comparability of cohorts through design or analysis</td><td colspan="2">Can be score with max 2 stars
Scored with: 1 star

Two cohorts were compared, one with prior PPV23 and one without, both comprising 15 participants. Also, children with congenital asplenia, HS, and splenectomy for other reasons were compared. However, the sample size seems too small to enable adequate comparison. Therefore this section was scored with 1 star.</td></tr>
<tr><td colspan="4">Outcome</td></tr>
<tr><td colspan="2">Assessment outcome</td><td>Follow up duration sufficient</td><td>Adequacy of follow up</td></tr>
</table>

**Score with max one star**<br>Scored with: **1 star**<br><br>*No protocol or statistical analysis plan are available. Outcomes seem predefined, have been described in the methods section and are usual for the study endpoint. Also, the assay for endpoint assessment has been described.*	**Scored with max one star**<br>Scored with: **0 stars**<br><br>*Peak response was measured 6-12 weeks after vaccination. Which seems rather long and wide-ranged for this endpoint. Especially after only one dose.*	**Scored with max one star**<br>Scored with: **1 star**<br><br>*All participants completed the study and had available samples.*
**Final assessment: 5/9 stars (FAIR)**		

**Study:** *Stanford E, Print F, Falconer M, Lamden K, Ghebrehewet S, Phin N, Baxter D, Helbert M, McCann R, Andrews N, Balmer P, Borrow R, Kaczmarski E. Immune response to pneumococcal conjugate vaccination in asplenic individuals. Hum Vaccin. 2009 Feb;5(2):85-91. doi: 10.4161/hv.5.2.6557. Epub 2009 Feb 7. PMID: 18758242*			
**Selection**			
**Representativeness exposed cohort**	**Selection non-exposed cohort**	**Ascertainment of exposure**	**Outcome not present at start study**
**Scored with max one star**<br>Scored with: **0 stars**<br><br>*The trial population comprised an adult asplenic population with various underlying conditions, age (18-81 years of age), and range of time from splenectomy (0.6-62 years). Almost 60% of the population is reported as having ongoing chronic illness, however the underlying condition have not been described and numbered. It is also no clear whether participants had additional immunocompromise. All these factors taken together, it is complicated to generalize the outcomes to other asplenic populations; age can affect vaccine response and the immunological state 0.6 years after splenectomy varies from 62 years after splenectomy. It must be noted that this problem is usual in the research regarding asplenia. Sample size is relatively large (111).*	**Scored with max one star**<br>Scored with: **0 stars**<br><br>*No control group was included.*	**Scored with max one star**<br>Scored with: **1 star**<br><br>*Vaccination was performed during the study.*	**Scored with max one star**<br>Scored with: **1 star**<br><br>*Baseline measurements were performed.*
**Comparability**			

**Comparability of cohorts through design or analysis**	**Can be score with max 2 stars**<br>Scored with: **1 stars**<br><br>*Results were adjusted for underlying condition (categorized in 4 main categories), time since splenectomy, and time since last PPV23 dose. This increases generalizability.*	
**Outcome**		
**Assessment outcome**	**Follow up duration sufficient**	**Adequacy of follow up**
**Score with max one star**<br>Scored with: **1 star**<br><br>*No protocol or statistical analysis plan are available. Outcomes seem to be predefined and are described in the method section. It is unclear which is the primary endpoint as multiple ways of assessing the immunogenicity are described and carried out in the study. Nevertheless, a gold standard is not available to assess this endpoint, it seems that all data is provided and described and outcomes are usual.*	**Scored with max one star**<br>Scored with: **1 star**<br><br>*Peak response was measured 4-6 weeks after vaccination for the primary outcome.*	**Scored with max one star**<br>Scored with: **1 star**<br><br>*Data was available from 109/111 individuals.*
**Final assessment: 6/9 stars (GOOD)**		

**Study:** *Meerveld-Eggink A, de Weerdt O, van Velzen-Blad H, Biesma DH, Rijkers GT. Response to conjugate pneumococcal and Haemophilus influenzae type b vaccines in asplenic patients. Vaccine. 2011 Jan 17;29(4):675-80. doi: 10.1016/j.vaccine.2010.11.034. Epub 2010 Nov 27. PMID: 21115060.*			
**Selection**			
**Representativeness exposed cohort**	**Selection non-exposed cohort**	**Ascertainment of exposure**	**Outcome not present at start study**
**Scored with max one star**<br>Scored with: **0 star**<br><br>*The trial population comprised 52 asplenic individuals (30% trauma, ITP (15%), Hodgkin's (19%), HS (11%) and other reasons) and 2 hyposplenic individuals (due to irradiation of the spleen for Hodgkin's lymphoma and Beta-thalassemia. Age range was 23-77 years and time since splenectomy was 5-76 years). It is unclear whether there were participants with additional immunocompromise as medication use and presence of active disease have not been described. Taken together the uncertainly regarding presence of additional immunocompromise during vaccination, it seems difficult to generalize these results to other asplenic cohorts.*	**Scored with max one star**<br>Scored with: **0 star**<br><br>*No control group has been included.*	**Scored with max one star**<br>Scored with: **1 star**<br><br>*Vaccination was performed during the study.*	**Scored with max one star**<br>Scored with: **1 star**<br><br>*Baseline measurements have been performed.*
**Comparability**			
**Comparability of cohorts through design or analysis**		**Can be score with max 2 stars**<br>Scored with: **1 star**<br><br>*Univariate analysis has been carried out for age at splenectomy, age at vaccination, reason for splenectomy (trauma vs other), prior PPV23 doses was performed. However, it is not described whether there were participants with additional immunocompromise and this has also not been tested.*	
**Outcome**			

Assessment outcome	Follow up duration sufficient	Adequacy of follow up
**Score with max one star**<br>Scored with: **1 star**<br><br>*No protocol or statistical analysis plan are available. Outcomes seem predefined and are usual for this endpoint, also the assay used has been sufficiently described. As can be observed in similar articles, multiple outcomes are considered for the endpoint and is has not been clearly described what the primary endpoint is. However, there is no gold standard and it seems that all information has been provided regarding different analyses.*	**Scored with max one star**<br>Scored with: **1 star**<br><br>*Sufficient, carried out after 3 weeks.*	**Scored with max one star**<br>Scored with: **0 star**<br><br>*It is described that all participants received the catch up vaccination, therefore it seems there was no loss to follow up. However, nr of included have not been displayed in the tables.*
**Final assessment: 5/9 stars (Fair)**		

Study: *Rosado MM, Gesualdo F, Marcellini V, Di Sabatino A, Corazza GR, Smacchia MP, Nobili B, Baronci C, Russo L, Rossi F, Vito RD, Nicolosi L, Inserra A, Locatelli F, Tozzi AE, Carsetti R. Preserved antibody levels and loss of memory B cells against pneumococcus and tetanus after splenectomy: tailoring better vaccination strategies. Eur J Immunol. 2013 Oct;43(10):2659-70. doi: 10.1002/eji.201343577. Epub 2013 Jul 25. PMID: 23813052.*

**Selection**			
**Representativeness exposed cohort**	**Selection non-exposed cohort**	**Ascertainment of exposure**	**Outcome not present at start study**
**Scored with max one star**<br>Scored with: **0 stars**<br><br>*The trial population consisted of 57 adult (age range 18-82 years) and 21 pediatric (age range 1-18 years) asplenic patients. Underlying conditions were mostly thalassemia, trauma, hereditary spherocytosis and nonhematological neoplasia. Time since splenectomy varied from 3-66 years for adults. Vaccination history and time since the last vaccination varied considerably, some being vaccinated before and others after splenectomy. Taken this together, it seems difficult to generalize the outcomes to other asplenic populations.*	**Scored with max one star**<br>Scored with: **1 star**<br><br>*Age matched healthy controls were included.*	**Scored with max one star**<br>Scored with: **1 star**<br><br>*Vaccination was adequately registered.*	**Scored with max one star**<br>Scored with: **0 stars**<br><br>*Baseline measurements were not performed due to study design.*
**Comparability**			
**Comparability of cohorts through design or analysis**		**Can be score with max 2 stars**<br>Scored with: **1 stars**<br><br>*Cohorts were compared with healthy controls and per vaccination history. .*	
**Outcome**			
**Assessment outcome**		**Follow up duration sufficient**	**Adequacy of follow up**

**Score with max one star**<br>Scored with: **1 star**<br><br>*Protocol and statistical analysis plan were not available. However, outcomes and objectives are usual and the assays were clearly described in detail.*	**Scored with max one star**<br>Scored with: **0 stars**<br><br>*Time since last vaccination varied considerably and was not clear described.*	**Scored with max one star**<br>Scored with: **1 star**<br><br>*It seems that for all participants the samples were available.*
**Final assessment: 5/9 (FAIR)**		

Study: *Rivera-Olivero IA, Del Nogal B, Fuentes M, Cortez R, Bogaert D, Hermans PW, Waard JH. Immunogenicity of a 7-valent pneumococcal conjugate vaccine (PCV7) and impact on carriage in Venezuelan children at risk of invasive pneumococcal diseases. Vaccine. 2014 Jun 30;32(31):4006-11. doi: 10.1016/j.vaccine.2014.04.027. Epub 2014 May 14. PMID: 24837505.*

Selection			
**Representativeness exposed cohort**	**Selection non-exposed cohort**	**Ascertainment of exposure**	**Outcome not present at start study**
**Scored with max one star**<br>Scored with: **0 stars**<br><br>*The sub trial population (of a total of 82 children at risk for IPD) of interest for this review comprised 22 young children with SCD. Demographics were only described for the total trial population and not for the subpopulation. Taken this together with the small sample size, it is unlikely that these results can be generalized to other populations with sickle cell disease.*	**Scored with max one star**<br>Scored with: **0 stars**<br><br>*No control group was included.*	**Scored with max one star**<br>Scored with: **1 star**<br><br>*Vaccination was performed during the study.*	**Scored with max one star**<br>Scored with: **1 star**<br><br>*Baseline characteristics were measured.*

Comparability	
**Comparability of cohorts through design or analysis**	**Can be score with max 2 stars**<br>Scored with: **0 stars**<br><br>*Not performed.*

Outcome		
**Assessment outcome**	**Follow up duration sufficient**	**Adequacy of follow up**
**Score with max one star**<br>Scored with: **1 star**<br><br>*Protocol and statistical analysis plan are not available. Outcomes are usual and seem predefined and are described in the methods section. As often observed in studies in this field, the primary endpoint has not been clearly defined and multiple assessments have been done. However, these are usual and seem reported with all information.*	**Scored with max one star**<br>Scored with: **0 stars**<br><br>*Peak response was measured 8 weeks after vaccination, which seem late.*	**Scored with max one star**<br>Scored with: **1 star**<br><br>*It is reported that all children completed the study and had available samples.*

Final assessment: 4/9 (FAIR)

**Study:** *Papadatou I, Piperi C, Alexandraki K, Kattamis A, Theodoridou M, Spoulou V. Antigen-specific B-cell response to 13-valent pneumococcal conjugate vaccine in asplenic individuals with β-thalassemia previously immunized with 23-valent pneumococcal polysaccharide vaccine. Clin Infect Dis. 2014 Sep 15;59(6):862-5. doi: 10.1093/cid/ciu409. Epub 2014 May 30. PMID: 24879786.*			
**Selection**			
**Representativeness exposed cohort**	**Selection non-exposed cohort**	**Ascertainment of exposure**	**Outcome not present at start study**
**Scored with max one star**<br>Scored with: **1 star**<br><br>*The trial population consisted of 39 adults with splenectomy and Beta-thalassemia 19-48 years of age, with the same history of PCV7 receipt, without other immunocompromise.*	**Scored with max one star**<br>Scored with: **0 stars**<br><br>*No control group was included.*	**Scored with max one star**<br>Scored with: **1 star**<br><br>*Vaccination was performed during the trial.*	**Scored with max one star**<br>Scored with: **1 star**<br><br>*Baseline measurements were taken.*
**Comparability**			
**Comparability of cohorts through design or analysis**		**Can be score with max 2 stars**<br>Scored with: **1 star**<br><br>*Sub analyses have been performed regarding number of previous PPV23 solely.*	
**Outcome**			
**Assessment outcome**		**Follow up duration sufficient**	**Adequacy of follow up**
**Score with max one star**<br>Scored with: **1 star**<br><br>*The protocol and statistical analyses plan are not available. outcomes seem predefined and are described in the method section. Also, a limited number of analyses have been performed. The assay has been described and adequately referred to.*		**Scored with max one star**<br>Scored with: **1 star**<br><br>*Sufficient at 28 days.*	**Scored with max one star**<br>Scored with: **1 star**<br><br>*It is described that all participants completed the schedule and had available samples.*
**Final assessment: 7/9 (GOOD)**			

**Study:** *Papadatou I, Orthopoulos G, Theodoridou M, Spoulou V. Long-lasting hyporesponsivenss induced by the 23-valent pneumococcal polysaccharide vaccine (PPV23) in asplenic patients with β-thalassemia major. Vaccine. 2015 Jul 31;33(32):3779-83. doi: 10.1016/j.vaccine.2015.06.100. Epub 2015 Jul 2. PMID: 26144903.*			
**Selection**			
**Representativeness exposed cohort**	**Selection non-exposed cohort**	**Ascertainment of exposure**	**Outcome not present at start study**
**Scored with max one star**<br>Scored with: **0 stars**<br><br>*Trial population comprised 24 splenectomised individuals with beta-thalassemia with a mean age of 26 years. This sample size is too small to allow generalization of the outcomes.*	**Scored with max one star**<br>Scored with: **0 stars**<br><br>*No control group has been included.*	**Scored with max one star**<br>Scored with: **1 star**<br><br>*Vaccination was performed during the study.*	**Scored with max one star**<br>Scored with: **1 star**<br><br>*Baseline measurements were performed.*
**Comparability**			
**Comparability of cohorts through design or analysis**		**Can be score with max 2 stars**<br>Scored with: **1 star**<br><br>*Sub analysis of the effect of age, sex, and vaccination history were performed.*	
**Outcome**			
**Assessment outcome**		**Follow up duration sufficient**	**Adequacy of follow up**
**Score with max one star**<br>Scored with: **1 star**<br><br>*The protocol and statistical analyses plan are not available. Outcomes are described in the methods section and are usual for this endpoint, and seem predefined.*		**Scored with max one star**<br>Scored with: **1 star**<br><br>*Sufficient, at 1 month.*	**Scored with max one star**<br>Scored with: **1 star**<br><br>*All participants completed the study.*
**Final assessment: 6/9 (GOOD)**			

<table>
<tr><td colspan="4">Study: De Montalembert M, Abboud MR, Fiquet A, Inati A, Lebensburger JD, Kaddah N, Mokhtar G, Piga A, Halasa N, Inusa B, Rees DC, Heath PT, Telfer P, Driscoll C, Al Hajjar S, Tozzi A, Jiang Q, Emini EA, Gruber WC, Gurtman A, Scott DA. 13-valent pneumococcal conjugate vaccine (PCV13) is immunogenic and safe in children 6-17 years of age with sickle cell disease previously vaccinated with 23-valent pneumococcal polysaccharide vaccine (PPSV23): Results of a phase 3 study. Pediatr Blood Cancer. 2015 Aug;62(8):1427-36. doi: 10.1002/pbc.25502. Epub 2015 Mar 23. Erratum in: Pediatr Blood Cancer. 2019 Feb;66(2):e27554. doi: 10.1002/pbc.27554. PMID: 25810327.</td></tr>
<tr><td colspan="4">Selection</td></tr>
<tr><td>Representativeness exposed cohort</td><td>Selection non-exposed cohort</td><td>Ascertainment of exposure</td><td>Outcome not present at start study</td></tr>
<tr><td>Scored with max one star
Scored with: 1 star

The trial population comprised 158 children with sickle cell disease in multiple countries (however, not countries in which a very high incidence of SCD is found). This seems to be a sufficient sample size to allow generalizability of the results to other populations with SCD.</td><td>Scored with max one star
Scored with: 0 stars

No control group was included.</td><td>Scored with max one star
Scored with: 1 star

Vaccination was performed during the trial.</td><td>Scored with max one star
Scored with: 1 star

Baseline measurements were performed.</td></tr>
<tr><td colspan="4">Comparability</td></tr>
<tr><td colspan="2">Comparability of cohorts through design or analysis</td><td colspan="2">Can be score with max 2 stars
Scored with: 1 star

Sub analysis of use of hydroxycarbamide and interval since last dose of PPV23.</td></tr>
<tr><td colspan="4">Outcome</td></tr>
<tr><td colspan="2">Assessment outcome</td><td>Follow up duration sufficient</td><td>Adequacy of follow up</td></tr>
<tr><td colspan="2">Score with max one star
Scored with: 1 star

No protocol or statistical analysis plan are available. Outcomes and study objectives are clearly described in the method section, are usual and seem predefined. The protocol was amended with addition of the 1 year follow up.</td><td>Scored with max one star
Scored with: 1 star

Peak response was measured one month after peak response.</td><td>Scored with max one star
Scored with: 0 stars

87% of the population was available for the immunogenicity endpoint.</td></tr>
<tr><td colspan="4">Final assessment: 6/9 (GOOD)</td></tr>
</table>

**Study:** *Nived P, Jørgensen CS, Settergren B. Vaccination status and immune response to 13-valent pneumococcal conjugate vaccine in asplenic individuals. Vaccine. 2015 Mar 30;33(14):1688-94. doi: 10.1016/j.vaccine.2015.02.026. Epub 2015 Feb 21. PMID: 25707692.*			
**Selection**			
**Representativeness exposed cohort**	**Selection non-exposed cohort**	**Ascertainment of exposure**	**Outcome not present at start study**
**Scored with max one star**<br>Scored with: **0 stars**<br><br>*For the prospective immunogenicity endpoint, the trial population comprised of 24 individuals with asplenia after splenectomy due to various conditions (malignant and benign). 9 of these were immunosuppressed and are displayed separately. Age varied from 22-80 years of age. The sample size seems too small to allow for generalization to other asplenic populations.*	**Scored with max one star**<br>Scored with: **0 stars**<br><br>*No control group was included.*	**Scored with max one star**<br>Scored with: **1 star**<br><br>*For the prospective part of the study design, the vaccination was administered during the study.*	**Scored with max one star**<br>Scored with: **1 star**<br><br>*Baseline measurements have been performed.*
**Comparability**			
**Comparability of cohorts through design or analysis**		**Can be score with max 2 stars**<br>Scored with: **0 stars**	
**Outcome**			
**Assessment outcome**		**Follow up duration sufficient**	**Adequacy of follow up**
**Score with max one star**<br>Scored with: **1 star**<br><br>*The protocol and statistical analysis plan are not available. Outcomes are usual and seem pre-defined, and have been described in the methods section. Also, the assay has been described and referred to.*		**Scored with max one star**<br>Scored with: **1 star**<br><br>*Sufficient, 4-6 weeks after vaccination.*	**Scored with max one star**<br>Scored with: **0 stars**<br><br>*91% completed the schedule and had available samples.*
**Final assessment: 4/9 (FAIR)**			

**Study:** Disu EA, Akodu SO, Arinola OG, Diaku-Akinwumi IN, Adedokun B, Olopade CO, Njokanma OF. Pneumococcal-specific IgG levels after 13-valent pneumococcal conjugate vaccination in Nigerian children with sickle cell disease. Paediatr Int Child Health. 2016 Nov;36(4):296-299. doi: 10.1080/20469047.2015.1106076. Epub 2016 Jan 8. PMID: 26745438.			
**Selection**			
**Representativeness exposed cohort**	**Selection non-exposed cohort**	**Ascertainment of exposure**	**Outcome not present at start study**
**Scored with max one star**<br>Scored with: **1 star**<br><br>*The trial population comprised 151 children between 5 months and 5 years of age with sickle cell disease. Also, the study was performed in Nigeria, which has a high incidence of SCD.*	**Scored with max one star**<br>Scored with: **1 star**<br><br>*50 (1/3) healthy controls were included.*	**Scored with max one star**<br>Scored with: **1 star**<br><br>*Vaccination was performed during the trial.*	**Scored with max one star**<br>Scored with: **1 star**<br><br>*Baseline characteristics were performed.*
**Comparability**			
**Comparability of cohorts through design or analysis**		**Can be score with max 2 stars**<br>Scored with: **1 star**<br><br>*Children with SCD are compared to HCs and within age groups.*	
**Outcome**			
**Assessment outcome**		**Follow up duration sufficient**	**Adequacy of follow up**
**Score with max one star**<br>Scored with: **1 star**<br><br>*No protocol or statistical analysis plan was available. however, outcomes are usual and described in the methods section, and seem predefined. The assay measures anti-pneumococcal rather than serotype specific antibodies. This seems also a clinically relevant endpoint, however less precise.*		**Scored with max one star**<br>Scored with: **0 stars**<br><br>*Two months after vaccination seems a bit late to capture the peak response.*	**Scored with max one star**<br>Scored with: **1 star**<br><br>*It is described that all children completed the study.*
**Final assessment: 7/9 (GOOD)**			

**Study:** *Sirima SB, Tiono A, Gansané Z, Siribié M, Zongo A, Ouédraogo A, François N, Strezova A, Dobbelaere K, Borys D. Immunogenicity and Safety of 10-valent Pneumococcal Nontypeable Haemophilus influenzae Protein D Conjugate Vaccine (PHiD-CV) Administered to Children With Sickle Cell Disease Between 8 Weeks and 2 Years of Age: A Phase III, Open, Controlled Study. Pediatr Infect Dis J. 2017 May;36(5):e136-e150. doi: 10.1097/INF.0000000000001518. PMID: 28403055.*			
**Selection**			
**Representativeness exposed cohort**	**Selection non-exposed cohort**	**Ascertainment of exposure**	**Outcome not present at start study**
**Scored with max one star**<br>Scored with: **1 star**<br><br>*The trial population comprised 150 children with sickle cell disease, included in different age categories (each 50). The study is performed in Burkina Fase, which has a high incidence of SCD.*	**Scored with max one star**<br>Scored with: **1 star**<br><br>*150 healthy age-matched controls were included.*	**Scored with max one star**<br>Scored with: **1 star**<br><br>*Vaccination was performed during the study.*	**Scored with max one star**<br>Scored with: **1 star**<br><br>*Baseline measurements were performed.*
**Comparability**			
**Comparability of cohorts through design or analysis**		**Can be score with max 2 stars**<br>Scored with: **1 star**<br><br>*Children with sickle cell disease were compared per age group and with healthy age-matched controls. Also IgG was compared to OPA..*	
**Outcome**			
**Assessment outcome**		**Follow up duration sufficient**	**Adequacy of follow up**

<table>
<tr><td colspan="2">Score with max one star
Scored with: 1 star

The protocol and statistical analysis plan were not available. Outcomes and objectives are described in the method section and seem usual and predefined. The assays performed are described and referred to.</td><td>Scored with max one star
Scored with: 1 star

Peak response was measured after 1 month after completion of primary schedule (which is adequate in case one aims to investigate the immunogenicity of the entire schedule) and after booster.</td><td>Scored with max one star
Scored with: 1 star

94.7% had available samples</td></tr>
<tr><td colspan="4">Final assessment: 8/9 (GOOD)</td></tr>
<tr><td colspan="4">Study: Gazi U, Karasartova D, Sahiner IT, Gureser AS, Tosun O, Derici MK, Dolapci M, Taylan Ozkan A. The effect of splenectomy on the levels of PCV-13-induced memory B- and T cells. Int J Clin Pract. 2018 May;72(5):e13077. doi: 10.1111/ijcp.13077. Epub 2018 Mar 13. PMID: 29532980.</td></tr>
<tr><td colspan="4">Selection</td></tr>
<tr><td>Representativeness exposed cohort</td><td>Selection non-exposed cohort</td><td>Ascertainment of exposure</td><td>Outcome not present at start study</td></tr>
<tr><td>Scored with max one star
Scored with: 0 stars

The trial population comprised of 14 splenectomised adults, 25-48 years of age, 3-36 years after splenectomy. This sample size does not allow for generalization of the outcomes to other asplenic populations.</td><td>Scored with max one star
Scored with: 1 star

A small group of 5 healthy controls was included.</td><td>Scored with max one star
Scored with: 1 star

Vaccination was performed during the study.</td><td>Scored with max one star
Scored with: 1 star

Baseline measurements have been performed.</td></tr>
<tr><td colspan="4">Comparability</td></tr>
<tr><td colspan="2">Comparability of cohorts through design or analysis</td><td colspan="2">Can be score with max 2 stars
Scored with: 1 stars

Due to the small sample size of both groups, the risk of bias does not seem to be lowered by comparison between both groups.</td></tr>
<tr><td colspan="4">Outcome</td></tr>
<tr><td colspan="2">Assessment outcome</td><td>Follow up duration sufficient</td><td>Adequacy of follow up</td></tr>
</table>

**Score with max one star**<br>Scored with: **1 star**<br><br>*The protocol and statistical analysis plan are not available. Outcomes and objectives seem predefined and the assays are described in detail.*	**Scored with max one star**<br>Scored with: **0 stars**<br><br>*8 weeks after the first vaccine seems a bit long and difficult to compare when after the second vaccine the peak response was measured after 4 weeks.*	**Scored with max one star**<br>Scored with: **1 star**<br><br>It seems that all participants completed the study
**Final assessment: 6/9 (FAIR)**		

Study: *Szenborn L, Osipova IV, Czajka H, Kharit SM, Jackowska T, François N, Habib MA, Borys D. Immunogenicity, safety and reactogenicity of the pneumococcal non-typeable Haemophilus influenzae protein D conjugate vaccine (PHiD-CV) in 2-17-year-old children with asplenia or splenic dysfunction: A phase 3 study. Vaccine. 2017 Sep 25;35(40):5331-5338. doi: 10.1016/j.vaccine.2017.08.039. Epub 2017 Aug 31. PMID: 28866290.*

Selection			
**Representativeness exposed cohort**	**Selection non-exposed cohort**	**Ascertainment of exposure**	**Outcome not present at start study**
**Scored with max one star**<br>Scored with: **0 stars**<br><br>*The trial population comprised 52 children in three age groups (2-4 year, 5-10 year, and 11-17 year old) of which 34 had asplenia an 15 had functional asplenia with various underlying conditions (1 SCD, gastro intestinal diseases, liver diseases and other hematological diseases). Degree of hyposplenia was not determined. It has not been described whether participants had additional immunocompromise (this was only a contra indication for the HCs). Taken this together, it is complicated to generalize the outcomes to other asplenic populations.*	**Scored with max one star**<br>Scored with: **0 stars**<br><br>*For each age group, investigators intended to include a HC group, however this has not been achieved for all groups and only in small numbers, it is therefore not likely that this has contributed to lowering the risk of bias.*	**Scored with max one star**<br>Scored with: **1 star**<br><br>*Vaccination was performed during the study.*	**Scored with max one star**<br>Scored with: **1 star**<br><br>*Baseline measurements have been performed.*
**Comparability**			
**Comparability of cohorts through design or analysis**		**Can be score with max 2 stars**<br>Scored with: **1 stars**<br><br>*Cohorts were compared within the age group.*	
**Outcome**			
**Assessment outcome**		**Follow up duration sufficient**	**Adequacy of follow up**

**Score with max one star**<br>Scored with: **1 star**<br><br>*No protocol or statistical analysis plan are available. however, outcomes are usual, described in the methods section and seem predefined. The assays have been sufficiently described and referred to. Notably, the immunogenicity was evaluated with a per-protocol analysis which would be less appropriate in case this study was randomized.*	**Scored with max one star**<br>Scored with: **1 star**<br><br>*Sufficient, peak response was measured 1 month after each dose.*	**Scored with max one star**<br>Scored with: **0 stars**<br><br>*86.5% (45/52) were included in the analysis.*
**Final assessment: 5/9 (FAIR)**		

**Study:** *Papadatou I, Lagousi T, Kattamis A, Spoulou V. Antibody persistence 5 years after a 13-valent pneumococcal conjugate vaccine in asplenic patients with β-thalassemia: assessing the need for booster. Ann Hematol. 2019 Mar;98(3):775-779. doi: 10.1007/s00277-019-03615-z. Epub 2019 Jan 25. PMID: 30683996.*			
**Selection**			
**Representativeness exposed cohort**	**Selection non-exposed cohort**	**Ascertainment of exposure**	**Outcome not present at start study**
**Scored with max one star**<br>Scored with: **1 star**<br><br>*The trial population comprised 34 splenectomised patients with beta-thalassemia (aged 24-53 years) who were vaccinated with PCV13 5 years previously in a study from which the current is a follow on study. Although sample size is borderline, this seems to be sufficient to generalize the outcome to other young- and middle adult populations. Also, vaccination history of PCV7 and PCV13 were similar for the entire cohort.*	**Scored with max one star**<br>Scored with: **0 stars**<br><br>*No control group was included.*	**Scored with max one star**<br>Scored with: **1 star**<br><br>*Vaccination was adequately recorded in the previous study.*	**Scored with max one star**<br>Scored with: **1 star**<br><br>*Baseline characteristics were performed during the previous study and described numerically in this publication.*
**Comparability**			
**Comparability of cohorts through design or analysis**		**Can be score with max 2 stars**<br>Scored with: **1 star**<br><br>*The effect of previous PPV23 doses and peak response 5 years previously were analyzed.*	
**Outcome**			
**Assessment outcome**		**Follow up duration sufficient**	**Adequacy of follow up**

**Score with max one star**<br>Scored with: **1 star**<br><br>*The study was registered on Clinical Trials.gov. Outcomes seem predefined and are usual. The assay is described.*	**Scored with max one star**<br>Scored with: **1 star**<br><br>*Follow up was 5 years after vaccination for the longevity of PCV13 induced immune response, according the trial objective. This was equal for all participants.*	**Scored with max one star**<br>Scored with: **1 star**<br><br>The trial had one sampling moment. For all participants enrolled, data was available.
**Final assessment: 7/9 (GOOD)**		

<table>
<tr><td colspan="4">Study: Takeshita K, Takeuchi N, Takahashi Y, Fukasawa C, Hishiki H, Hoshino T, Ishiwada N, Shimojo N. Pneumococcal serotype-specific IgG and opsonophagocytic activity in young Japanese patients with asplenia. Hum Vaccin Immunother. 2021 Oct 3;17(10):3687-3691. doi: 10.1080/21645515.2021.1943989. Epub 2021 Jul 2. PMID: 34213395; PMCID: PMC8437487.</td></tr>
<tr><th colspan="4">Selection</th></tr>
<tr><th>Representativeness exposed cohort</th><th>Selection non-exposed cohort</th><th>Ascertainment of exposure</th><th>Outcome not present at start study</th></tr>
<tr><td>Scored with max one star
Scored with: 0 stars

The trial population comprised 23 young asplenic patients with a heterogeneous vaccination history consisting of PCV7/13 and PPV23, with various time since last vaccination. Two patients were splenectomised and 21 had congenital asplenia. Taken this together, is seems complicated to generalize the outcomes to other asplenic populations.</td><td>Scored with max one star
Scored with: 0 stars

No control group was included and with only 5 participants that appeared to never have received vaccination,</td><td>Scored with max one star
Scored with: 1 star

Vaccination was adequately registered, however very variable.</td><td>Scored with max one star
Scored with: 0 stars

No clear baseline measurements are available.</td></tr>
<tr><th colspan="4">Comparability</th></tr>
<tr><td colspan="2">Comparability of cohorts through design or analysis</td><td colspan="2">Can be score with max 2 stars
Scored with: 0 star

Seen the very small sample sizes per subgroup, is seems unlikely that these comparisons have contributed to the decrease of bias/risk of bias.</td></tr>
<tr><th colspan="4">Outcome</th></tr>
<tr><th colspan="2">Assessment outcome</th><th>Follow up duration sufficient</th><th>Adequacy of follow up</th></tr>
<tr><td colspan="2">Score with max one star
Scored with: 1 star

The protocol and statistical analysis plan are not available. the assays have been clearly described.</td><td>Scored with max one star
Scored with: 0 stars

Follow up was very variable and therefore the clinical relevance is difficult to quantitate.</td><td>Scored with max one star
Scored with: 1 star

As the study consisted of one measurement and all samples were available.</td></tr>
<tr><td colspan="4">Final assessment: 3/9 (FAIR)</td></tr>
</table>

PRISMA checklist 2020

Section and Topic	Item #	Checklist item	Location where item is reported
**TITLE**			
Title	1	Identify the report as a systematic review.	abstract
**ABSTRACT**			
Abstract	2	See the PRISMA 2020 for Abstracts checklist.	
**INTRODUCTION**			
Rationale	3	Describe the rationale for the review in the context of existing knowledge.	Pg 2. Introduction
Objectives	4	Provide an explicit statement of the objective(s) or question(s) the review addresses.	Pg 3. Introduction
**METHODS**			
Eligibility criteria	5	Specify the inclusion and exclusion criteria for the review and how studies were grouped for the syntheses.	Section 2.3
Information sources	6	Specify all databases, registers, websites, organisations, reference lists and other sources searched or consulted to identify studies. Specify the date when each source was last searched or consulted.	Section 2.4
Search strategy	7	Present the full search strategies for all databases, registers and websites, including any filters and limits used.	Supplementary materials
Selection process	8	Specify the methods used to decide whether a study met the inclusion criteria of the review, including how many reviewers screened each record and each report retrieved, whether they worked independently, and if applicable, details of automation tools used in the process.	Section 2.4 and protocol (PROSPERO)
Data collection process	9	Specify the methods used to collect data from reports, including how many reviewers collected data from each report, whether they worked independently, any processes for obtaining or confirming data from study investigators, and if applicable, details of automation tools used in the process.	Section 2.4 and protocol (PROSPERO) pg 7.
Data items	10a	List and define all outcomes for which data were sought. Specify whether all results that were compatible with each outcome domain in each study were sought (e.g. for all measures, time points, analyses), and if not, the methods used to decide which results to collect.	Section 2.3 and protocol (PROSPERO) Pg 4 and 5
	10b	List and define all other variables for which data were sought (e.g. participant and intervention characteristics, funding sources). Describe any assumptions made about any missing or unclear information.	protocol (PROSPERO) pg 7.
Study risk of bias assessment	11	Specify the methods used to assess risk of bias in the included studies, including details of the tool(s) used, how many reviewers assessed each study and whether they worked independently, and if applicable, details of automation tools used in the process.	Section 2.5, supplementary materials and

Section and Topic	Item #	Checklist item	Location where item is reported
			protocol (PROSPERO) Pg 6
Effect measures	12	Specify for each outcome the effect measure(s) (e.g. risk ratio, mean difference) used in the synthesis or presentation of results.	Section 2.4 and protocol (PROSPERO) Pg 3-5
Synthesis methods	13a	Describe the processes used to decide which studies were eligible for each synthesis (e.g. tabulating the study intervention characteristics and comparing against the planned groups for each synthesis (item #5)).	protocol (PROSPERO) Pg 6, section 2.3
	13b	Describe any methods required to prepare the data for presentation or synthesis, such as handling of missing summary statistics, or data conversions.	Not applicable
	13c	Describe any methods used to tabulate or visually display results of individual studies and syntheses.	protocol (PROSPERO) Pg 6
	13d	Describe any methods used to synthesize results and provide a rationale for the choice(s). If meta-analysis was performed, describe the model(s), method(s) to identify the presence and extent of statistical heterogeneity, and software package(s) used.	Section 2.6
	13e	Describe any methods used to explore possible causes of heterogeneity among study results (e.g. subgroup analysis, meta-regression).	Not applicable
	13f	Describe any sensitivity analyses conducted to assess robustness of the synthesized results.	Not applicable
Reporting bias assessment	14	Describe any methods used to assess risk of bias due to missing results in a synthesis (arising from reporting biases).	Section 2.5
Certainty assessment	15	Describe any methods used to assess certainty (or confidence) in the body of evidence for an outcome.	Not applicable
**RESULTS**			
Study selection	16a	Describe the results of the search and selection process, from the number of records identified in the search to the number of studies included in the review, ideally using a flow diagram.	Section 3.1
	16b	Cite studies that might appear to meet the inclusion criteria, but which were excluded, and explain why they were excluded.	Not applicable, we did not have these kind of studies
Study characteristics	17	Cite each included study and present its characteristics.	Table 1.

Section and Topic	Item #	Checklist item	Location where item is reported
Risk of bias in studies	18	Present assessments of risk of bias for each included study.	Section 3.2 and table 2
Results of individual studies	19	For all outcomes, present, for each study: (a) summary statistics for each group (where appropriate) and (b) an effect estimate and its precision (e.g. confidence/credible interval), ideally using structured tables or plots.	Table 4
Results of syntheses	20a	For each synthesis, briefly summarise the characteristics and risk of bias among contributing studies.	Results section
	20b	Present results of all statistical syntheses conducted. If meta-analysis was done, present for each the summary estimate and its precision (e.g. confidence/credible interval) and measures of statistical heterogeneity. If comparing groups, describe the direction of the effect.	Not applicable
	20c	Present results of all investigations of possible causes of heterogeneity among study results.	Results section
	20d	Present results of all sensitivity analyses conducted to assess the robustness of the synthesized results.	Results section
Reporting biases	21	Present assessments of risk of bias due to missing results (arising from reporting biases) for each synthesis assessed.	Results and discussion
Certainty of evidence	22	Present assessments of certainty (or confidence) in the body of evidence for each outcome assessed.	Not applicable
**DISCUSSION**			
Discussion	23a	Provide a general interpretation of the results in the context of other evidence.	Discussion
	23b	Discuss any limitations of the evidence included in the review.	Discussion
	23c	Discuss any limitations of the review processes used.	Discussion
	23d	Discuss implications of the results for practice, policy, and future research.	Discussion
**OTHER INFORMATION**			
Registration and protocol	24a	Provide registration information for the review, including register name and registration number, or state that the review was not registered.	Methods
	24b	Indicate where the review protocol can be accessed, or state that a protocol was not prepared.	Methods
	24c	Describe and explain any amendments to information provided at registration or in the protocol.	PROSPERO
Support	25	Describe sources of financial or non-financial support for the review, and the role of the funders or sponsors in the review.	Section 2.7
Competing interests	26	Declare any competing interests of review authors.	Discussion
Availability of data, code and other materials	27	Report which of the following are publicly available and where they can be found: template data collection forms; data extracted from included studies; data used for all analyses; analytic code; any other materials used in the review.	All available content is contained in the

Supplementary materials: *The immunogenicity of pneumococcal conjugate vaccinations in patients with asplenia and hyposplenia: a systematic review*

Section and Topic	Item #	Checklist item	Location where item is reported
			manuscript, protocol or supplementary materials

From: Page MJ, McKenzie JE, Bossuyt PM, Boutron I, Hoffmann TC, Mulrow CD, et al. The PRISMA 2020 statement: an updated guideline for reporting systematic reviews. BMJ 2021;372:n71. doi: 10.1136/bmj.n71. This work is licensed under CC BY 4.0. To view a copy of this license, visit https://creativecommons.org/licenses/by/4.0/

Supplementary Materials 2: Outcomes

1. Table S1. Characteristics of included studied (patients and design)
2. Table S2, Outcomes of included studies
3. Table S3, Immunogenicity outcomes (extensive) per study: Serology outcomes <0.35 ug/mL are reported in red, 0.35-1.00 ug/mL are reported in orange. OPA outcomes <8 are reported in red. Details regarding the trials can be found in the main article.

Supplementary materials: *The immunogenicity of pneumococcal conjugate vaccinations in patients with asplenia and hyposplenia: a systematic review*

Characteristics of included studies:

Table S1. Study characteristics (randomized and nonrandomized trials)

Study	N	Population	Outcomes	Assays	Definition adequate response	Measurements
*1a. Studies assessing immunogenicity of PCV in splenectomised/hyposplenic individuals with mixed comorbidity**						
			**PCV7**			
Smets et al. (2007) Belgium<br><br>**RCT, single center**<br><br>***Vaccine***	21	Children < 5 years of age with asplenia or hyposplenia<br><br>Vaccination history: PPV23 >3 years	PS specific IgG GMCs after **PCV7 or PPV23**	Serology: modified ELISA	≥ 4x fold rise<br><br>Protective: IgG GMC ≥0.2 μg/mL (PCV) or ≥1.0 μg/mL (PPV)	Serotypes: 4, 6B, 9V, 14, 18C, 19F, and 23F<br><br>Measurements: baseline, and 1 and 6 months after vaccination.
Mikoluc et al (2008) Poland<br><br>**Prospective clinical trial, single center**<br><br>***European Journal of Clinical Microbiology and Infectious Disease.***	30	Splenectomised or congenitally asplenic children 4 months-19 years of age<br><br>Vaccination history: 15/30 received previous PPV23	PS specific IgG GMCs after **PCV7**<br><br>Effect of previous receipt of PPV23	Serology: ELISA with 22F pre-adsorption	IgG GMC ≥ 0.35 μg/mL	Serotypes: 4, 6B, 9V, 14, 18C, 19F, and 23F<br><br>Measurements: baseline, and 6-12 weeks after vaccination.

Stanford et al (2009)<br>***United Kingdom***<br><br>**Prospective clinical trial, multi center**<br><br>***Human vaccines***	111	Splenectomised adults	PS specific IgG GMCs after **PCV7 and PPV23**	Serology: ELISA with pneumococcal cell wall polysaccharide and 22F pre-adsorption (WHO)	Not defined, IgG GMC ≥0.35 μg/mL and ≥1.00 μg/mL mentioned	Serotypes: 4, 6B, 9V, 14, 18C, 19F, 23F (PCV7) and 1 and 5 (non PCV7)<br><br>Measurements: baseline, and at 3-6 weeks after both vaccines.
Meerveld-Eggink et al (2011)<br>***The Netherlands***<br><br>**Prospective clinical trial, multi center**<br><br>***Vaccine***	54	Splenectomised or radiation-induced hyposplenic adults	PS specific IgG GMCs after **2 doses PCV7 and PPV23**	Serology: Luminex xMap technology	IgG GMC ≥0.35 μg/mL and ≥1.00 μg/mL	Serotypes: 3, 4, 6B, 9V, 14, 18C, 19F, and 23F, subset: 1, 7F, 8, 9N, 12F and 19A<br><br>Measurements: baseline, and at 3 weeks after each vaccination.
Rosado et al (2013)<br>***Italy***<br><br>**Cross sectional trial with retrospective analysis of vaccination history**<br><br>***European Journal of Immunology***	144	Splenectomised adults and children<br><br>*Vs*<br><br>Healthy age-matched controls<br><br>Vaccination history:<br>**Adults**: PPV23 before or after splenectomy<br>**Children**: PPV23 and/or PCV, before or after splenectomy	Antipneumococ-cal specific IgM and IgG memory B-cells and IgG GMCs after **previous PCV7 and/or PPV23**	B-cell counts, IgG and IgM secretion and pneumococcal specificity: ELISPOT and flowcytometry analysis (staining for CD19, CD27, IgM, and IgD)<br><br>Serology: VaccZyme™ Anti-PCP IgG Enzyme Immunoassay kit.	Not defined	Serotypes: not separately measured<br><br>Measurement: cross sectionally, for adults 3.3-65.7 years after splenectomy, for children this was not described.
**PCV10, PCV13**						

Nived et al (2015) Sweden<br><br>**Prospective observational trial with retrospective analysis, single center**<br><br>***Vaccine***	24	Splenectomised children (>11 yrs) and adults	PS specific IgG GMCs after **PCV13** or **previous PCV7 and/or PPV23**	Serology: Luminex (nonaplex assay)	IgG GMC ≥0.35 µg/mL	Serotypes: 1, 3, 4, 5, 6B, 7F, 9V, 14, 18C, 19A, 19F, and 23F<br><br>Measurements:<br>**Group 1**: baseline, and at 4-6 weeks after PCV13<br>**Group 2**: cross sectionally, various time point after PCV7 and/or PPV23
Gazi et al (2017) Turkey<br>**Prospective clinical trial, single center**<br><br>***International journal of clinical practice***	19	Splenectomised adults<br><br>*Vs*<br><br>Healthy adult controls	PCV13-specific memory IgG B-cells and memory IFN-y T-cells after **PCV13 and PPV23**	PCV13 specific B-cells, T-cells and cytokines: ELISPOT	Not defined	Serotypes: Not individually tested<br><br>Measurements: baseline, at 8 weeks, and 12 weeks.
Szenborn et al (2017) Poland and Russian Federation<br><br>**Open label prospective controlled trial, phase 3, multi center**<br><br>***Vaccine***	52	Children 2-17 years of age with asplenia, hyposplenia or complement deficiencies	PS specific IgG GMCs and PS specific OPA GMTs after **PCV10**	Serology: ELISA with 22F pre-adsorption<br>OPA: multiplex opsonophagocytic killing assay	IgG GMC ≥0.2* µg/mL<br><br>OPA GMT ≥8 µg/mL<br><br>*equivalent to ≥0.35 µg/mL, by non-22F-ELISA WHO reference lab	Serotypes: 1, 4, 5, 6B, 7F, 9V, 14, 18C, 19F, and 23F (PCV10) and PS 6A and 19A (non-PCV10)<br><br>Measurements: baseline, and at 1 month after each dose.
Takeshita et al (2021) Japan<br><br>**Observational, cross sectional trial, single center**<br><br>***Human vaccines and immuno-therapeutics***	123	Infants, children and young-adults with asplenia<br><br>*Vs*<br><br>Healthy age-matched controls	PS specific IgG GMCs and PS specific OPA GMTs after **previous PCV7, PCV13 or PPV23**.	Serology: ELISA with C-polysaccharide and 22F pre-adsorption (WHO)<br><br>OPA: multiplex opsonophagocytic killing assay	IgG GMC ≥0.2* µg/mL<br><br>OPA GMT ≥8 µg/mL<br><br>*equivalent to ≥0.35 µg/mL when using ELISA without 22F absorption	Serotypes: 1, 3, 5, 6A, 7 F, 19A (serology), 3, 5, 19[a] (opsonophagocytic activity)<br><br>Measurement: cross sectionally, various time after vaccination.

1b. Studies assessing immunogenicity of PCV in splenectomised individuals with spherocytosis and hemoglobinopathies

Study		Population	Outcomes	Assays	Definition adequate response	Measurements
**PCV7**						
Stoehr et al (2005) Germany<br><br>**Prospective open clinical trial, multi center**<br><br>***British Journal of Haematology***	42	Splenectomised children 2-18 yrs of age with Hereditary Spherocytosis<br><br>*vs*<br><br>Healthy splenectomised controls 12-24 yrs of age<br><br>Vaccination history: all received PPV23 prior to splenectomy	PS specific IgG GMCs after **PCV7 and PPV23**	Serology: assay not described	IgG GMC ≥1.0 µg/mL	Serotypes: 4, 6B, 9V, 14, 18C, 19F, and 23F<br><br>Measurements baseline, 8 weeks after PCV, and 4 weeks after PPV23.
Orthopoulos et al (2009) Greece<br><br>**RCT, single center**<br><br>***Vaccine***	35	Splenectomised adults and children (>12 yrs) with β-thalassemia<br><br>*vs*<br><br>Non-splenectomised PPV naïve controls with β-thalassemia >12 yrs of age	PS specific IgG GMCs after **PCV7 and 2 doses PPV23 or 2 doses PCV7 and PPV23**	Serology and avidity: modified ELISA	Not defined	Serotypes: 6B, 9V, 14, 19F and 23F<br><br>Measurements: baseline, and 4-6 weeks after each vaccination.

			**PCV13**			
Papadatou et al (2014) Greece ***Follow up Orthopoulos 2009** <br>**Prospective clinical trial** <br>***Clinical infectious diseases***	39	Splenectomised adults with β-thalassemia major <br>Vaccination history: PCV7 and 1-4 PPV23 doses (1-11 years ago)	PS specific IgG memory B-cell and IgG GMCs after **PCV13**	Serology and memory B-cells: ELISPOT	IgG GMC ≥ 0.35 μg/mL	Serotypes: 3, 9V, 19A, 19F, and 23F (serology and B-cells) <br>Measurements: baseline, at day 7, and day 28.
Papadatou et al (2015) Greece ***Follow up Orthopoulos 2009** <br>**Prospective clinical trial** <br>***Vaccine***	24	Splenectomised adults with β-thalassemia major <br>Vaccination history: (7 years earlier) PCV7+PCV7/ PPV23	PS specific IgG GMCs after **PCV13**	Serology: ELISA (WHO)	IgG GMC ≥ 0.35 μg/mL	Serotypes*: 9V, 19F and 23F <br>Measurements: baseline, and one month after PCV13. <br>*PS with hypo-responsiveness in Orthopoulos 2009
Rezai et al (2016) Iran <br>**RCT, crossover, single center** <br>***Caspian Journal of Internal Medicine***	47	Splenectomised adults with thalassemia major <br>Vaccination history: previous vaccination > 5 years	Antipneumococ-cal IgG GMCs (not PS specific) after **PCV13 and PPV23**	Serology: VaccZyme anti-PCP IgG ELISA	IgG GMC ≥ 0.35 μg/mL	Serotypes: not individually tested <br>Measurements: baseline, at 8 weeks, and 16 weeks.

Sari et al (2017) ***Indonesia*** <br>**RCT, single center** <br>***Vaccine***	56	Splenectomised children (>12 yrs) and adults with β-thalassemia major	Antipneumococ-cal IgG GMCs (not PS specific) after **PCV13 and zinc or placebo**	Serology: Pneumococcus Immunopotency level/E-DG-MZ-001/04-04 kit (ZenTech)	IgG GMC ≥250 mU/mL	Serotypes: not individually tested <br>Measurements: baseline, and 12 weeks.
Papadatou et al (2019) ***Greece*** ***Follow up Papadatou 2014/2015** <br>**Prospective observational trial** <br>***Annals of Hematology***	34	Splenectomised adults with β-thalassemia major <br>Vaccination history: PCV13 5 years previously, PCV7 12 years previously, 1-4 doses of PPV23	PS specific IgG GMCs 5 years after **PCV13**	Serology: ELISA (WHO)	IgG GMC ≥ 1.0 μg/mL	Serotypes: 3, 9V, 19A, 19F, and 23F <br>Measurements: cross sectionally, approximately 5 years after PCV13.

1c. Studies assessing PCV immunogenicity in individuals with sickle cell disease

Study	N	Population	Outcomes	Assays	Definition adequate response	Measurements
**PCV7**						
Vernacchio et al (1998) ***United States*** <br>**RCT, single center** <br>***The journal of Pediatrics***	23	Children ≥ 2 years of age with SCD	PS specific IgG GMCs after **2 PCV7 doses and PPV23**	Serology: ELISA with pre-adsorption of C-polysaccharide	Not defined	Serotypes:4, 6B, 9V, 14, 18C, 19F, 23F, 23V, 1, 15B <br>Measurements: before each vaccination, and 3-6 weeks after PPV23.

Vernacchio et al (2000) ***United States*** <br>**RCT, single center** <br>*The Journal of Infectious Diseases*	23	Children ≥ 2 years of age with SCD	PS specific OPA GMTs after **2 PCV7 doses and PPV23** <br>Correlation of OPA GMTs and IgG GMCs.	OPA: flowcytometry	Not defined	Serotypes: 4, 6B, 9V, 14, 18C, 19F, and 23F <br>Measurements: prior to each vaccination, and 3-6 weeks after PPV23.
Nowak-Wegrzyn et al (2000) ***United States*** <br>**Prospective clinical trial, multi center** <br>*Clinical and diagnostic laboratory immunology*	48	Infants 2-12 months of age with SCD <br>*vs* <br>African-American age matched controls without SCD	Opsonophagocytic activity in % after **sequential PCV7 and PPV23, and correlation with PS specific IgG GMCs**	Serology: modified ELISA <br>OPA: assay using heat inactivation	Not defined	Serotypes: 6B and 14 (serology and OPA) <br>Measurements: **Group 1**: at 2, 4, 6, 7, 12, 24, and 25 months of age. **Group 2**: at 12, 13, 24, and 25 months of age.
O'Brien et al (2000) ***The United States*** <br>**Prospective open label clinical trial, multi center** <br>*Pediatrics*	61	Infants with SCD 2-12 months of age <br>*vs* <br>Healthy age-matched controls	PS specific IgG GMCs after **sequential PCV7 and PPV23**	Serology: modified ELISA	IgG GMC ≥0.15 μg/mL	Serotypes: 4, 6B, 9V, 14, 18C, 19F, and 23F <br>Measurements: before each vaccination, and one month after primary series, at 12 months of age, and 1 month after PPV23.

Reinert et al (2007) ***France*** <br>**Open label prospective clinical trial, multi center** <br>***The pediatric infectious disease journal***	51	Infants ≥ 2 months of age with SCD	PS specific IgG GMCs and PS specific OPA GMTs after **sequential PCV7 and PPV23**	Serology: ELISA <br>OPA: assay not described	IgG GMC ≥0.35 μg/mL	Serotypes: 4, 6B, 9V, 14, 18C, 19F, and 23F (serology) and PS 6B, 14, 19F and 23F (OPA) <br>Measurements: baseline, at month 5, baseline prior to booster, and at month 1 after booster.
Rivera-Olivero et al (2014) ***Venezuela*** <br>**Prospective open label clinical trial, single center** <br>***Vaccine***	22	Children with SCD (subgroup of a larger cohort comprising children at high risk for IPD	PS specific IgG GMCs after **sequential PCV7**	Serology: ELISA with pre-adsorption of C-polysaccharide and 22F (WHO)	IgG GMC ≥ 0.35 μg/mL	Serotypes: 4, 6B, 9V, 14, 18C, 19F, and 23F <br>Measurements: baseline, and 1 month after completion of the schedule.
**PCV10, PCV13, PCV15**						
De Montalembert et al. (2015) ***US, UK, Lebanon, Egypt, France, Saudi Arabia*** <br>**Open-label, single-arm, prospective clinical trial, phase 3, multi center** <br>***Pediatric Blood Cancer***	158	Children 6-17 years of age with SCD	PS specific IgG GMCs and PS specific OPA GMTs after **2 doses of PCV13**	Serology: assay not described <br>OPA: assay not described	Not defined	Serotypes: 1, 3, 4, 5, 6A, 6B, 7F, 9V, 14, 18C, 19A, 19F, and 23F (serology and OPA) <br>Measurements: baseline, month 1, month 7, and 1 year after completion of the schedule.

Disu et al. (2016) Nigeria<br><br>**Prospective open label clinical trial, multi center**<br><br>***Paediatrics and International Child Health***	200	Children with SCD 5 months-5 years of age<br><br>*vs*<br><br>Healthy age-matched controls	Antipneumococ- cal IgG GMCs (not PS specific) after **PCV13**	Serology: ELIZEN kit (ZenTech)	Seroconversion: IgG GMC fold rise of ≥4<br><br>Protective: IgG GMC ≥200 µg/mL	Serotypes: not individually tested.<br><br>Measurements: baseline and at 2 months.
Sirima et al (2017) Burkina Faso<br>**Open label prospective clinical trial, phase 3, single center**<br><br>***Pediatric Infectious Disease Journal***	300	Infants 2-23 months of age with SCD<br><br>*vs*<br><br>Healthy age-matched controls	PS specific IgG GMCs after **sequential PCV10**	Serology: ELISA with pre-adsorption 22F<br><br>OPA: assay not described	IgG GMC ≥0.2* µg/mL<br><br>*Equivalent to ≥0.35 µg/mL threshold with this ELISA	Serotypes: 1, 4, 5, 6B, 7F, 9V, 14, 18C, 19F, 23F (PCV10), 6A, 19A (PCV10 related) (serology and OPA)<br><br>Measurements: baseline, at month 1 after primary and booster vaccination.
Melica et al. (2022) France<br><br>**RCT, open label, phase 2, single center**<br><br>***Clinical infectious Diseases***	128	Adults with major SCD (SS, SC, Sβ-thalassemia)	PS specific IgG GMCs and PS specific OPA GMTs after **PCV13 and PPV23**	Serology: Luminex-based assay<br><br>OPA: microcolony opsonophagocytic assay	IgG GMC fold rise of ≥4 to ≥10 PS (shared PS).	Serotypes: 1, 3, 4, 5, 6A, 6B, 7F, 9V, 14, 18C, 19A, 19F, and 23F (both assays)<br><br>Measurements: baseline, at week 4, 8, 24, and 96

Quinn et al (2023) Brazil, Colombia, Dominican Republic, Greece, Italy, Panama, and the US<br><br>**RCT, phase 3, multi center**<br><br>***Blood Advances***	103	Children 2-17 years of age with SCD or sickle cell-beta thalassemia	PS specific IgG GMCs and PS specific OPA GMTs after **PCV13 or PCV15**	Serology: pneumococcal electrochemi-luminescence v2.0 assay<br><br>OPA: multiplex OPA assay	Not defined	Serotypes: 1, 3, 4, 5, 6A, 7F, 9V, 14, 18C, 19A, 19F, 23F and 6B (shared) and 22F and 33F (PCV15 PS) (both assays)<br><br>Measurements: baseline, at day 30

Smets: splenectomy due to hereditary spherocytosis, immune thrombocytopenic purpura, pyruvate kinase deficiency, or asplenia due to sickle cell disease. **Mikoluc**: splenectomy due to hereditary spherocytosis, portal hypertension, pancreas tumor, splenic cyst, or asplenia due to congenital asplenia **Szenborn**: splenectomy due to trauma, hematological diseases, auto-immune disease or asplenic due to SCD **Stanford:** (splenectomy-vaccination 0.6-62.7 years): trauma, benign processes requiring resection, malignant disease, mixed pathology – during vaccination 57.7% had unspecified ongoing chronic disease requiring treatment **Meerveld-Eggink** (splenectomy-vaccination 1-51 years): trauma (30%), thrombotic thrombocytopenic purpura, hereditary spherocytosis, functional (n=radiation-induced). **Nived:** all splenectomy and immunocompetent (solid organ malignancy, benign process requiring resection, other hematological disease, trauma) **Rosado:** splenectomy due to β-thalassemia, trauma, hereditary spherocytosis, solid organ malignancy, congenital asplenia (n=1)
Abbreviations: PCV7/10/13/15= 7-valent, 10-valent, 13-valent, 15-valent pneumococcal conjugate vaccine, RCT= randomized controlled trial, PPV23= 23 valent pneumococcal polysaccharide vaccine, ELISA= enzyme-linked immunosorbent assay, PS=pneumococcal serotype, GMC=Geometric Mean Concentration, WHO= World Health Organization, vs=versus, OPA= opsonophagocytic activity, GMT= Geometric Mean Titer, SCD= sickle cell disease, IPD= invasive pneumococcal disease, US= United States, UK= United Kingdom, HS=Hereditary spherocytosis

S2. Outcomes per study

Table S2. Main outcomes and conclusions for all included studies

Trial population	Vaccination schedule	Findings and conclusion
4a. Main outcomes: splenectomised/hyposplenic with mixed comorbidity		
Smets et al. (2007)<br><br>Total N = 21*<br>**Group 1 N=11**<br>Median age: 12.6y<br>**Group 2 N=8**<br>Median age: 11.7y<br>**19 accessible for analysis*	**Group 1**: PCV7<br>**Group 2**: PPV23	Baseline: no significant differences in % of GMCs ≥1.00 μg/mL between groups<br><br>At month 1:<br>**Group 1:** GMCs increased significantly for PS 6B and 23F, 78-100% of GMCs were ≥1.00 μg/mL, for PS 6B GMCs significantly more ≥4 fold increase<br>**Group 2:** no significant increase in GMC for any PS, 75-100% of GMCs were ≥1.00 μg/mL<br><br>At month 6:<br>**Group 1:** 80-100% of GMCs were ≥1.00 μg/mL<br>**Group 2:** 62-100% of GMCs were ≥1.00 μg/mL<br><br>**Conclusion**: PCV7 induced a broader and higher IgG response for PCV7-PS compared to PPV23.
Mikoluc et al. (2008)<br><br>Total N=30<br>Median age: 12y (4.5m -19.5y)	**All**: PCV7	Baseline: 36-80% of GMCs ≥0.35 μg/mL<br><br>At week 6-12: 28/30 had an PS-specific IgG ≥0.35 μg/mL for all PS, GMCs increased significantly for all PS, a ≥2 fold increase was found in 57-84% depending on the PS. Previous PPV23 (5.2y ± 3.4 previously) did not affect the response<br><br>**Conclusion**: PCV7 induced a significant IgG response for all PCV7-PS.

Stanford et al. (2009)<br><br>Total N=111<br>Median age: 54.8y (18.1-81.8y)	**All**: PCV7 + PPV23*<br>(at month 0, 6)<br><br>**if not received in last 5 years*	Baseline: 77-97% of GMCs was ≥0.35 µg/mL<br><br>After PCV: for PCV7-PS 91-99% of GMCs was ≥0.35 µg/mL and 71-95% was ≥1.00 µg/mL, GMCs for all PS increased significantly except for PS 1 and 5 (non PCV7), a ≥2 fold rise was found in 27-69% of participants<br><br>After PPV23 at 7-8 months (N=33): GMCs and % with PS-specific IgG ≥1.0 µg/mL did not significantly increase<br><br>Time (years) since splenectomy and last dose negatively affected post-vaccination IgG levels, for PS 9V and 18C respectively.<br><br>**Conclusion**: PCV7 induced a significant IgG response for all PCV7-PS but not for non-PCV7 PS, no significant contribution of PPV23.
Meerveld-Eggink et al. (2011)<br><br>Total N=54<br>Median age: 51.5y (23 -77y)	**All**: PCV7 + PCV7+ PPV23<br>(at week 0, 6, and month 6)	At baseline: 56% of GMCs ≥0.35 µg/mL, and 17% ≥1.0 µg/mL for all PS<br><br>After 1st PCV7: GMCs significantly increased for all PS, and 46% of GMCs ≥1.0 µg/mL.<br>After 2nd PCV7: only additional significant increase in GMC for PS 4<br>After PPV23: no further significant increase in GMCs<br><br>**Conclusion**: PCV7 induced a significant IgG response for all PCV7-PS, minor contribution of 2nd PCV7 and no contribution of PPV23.
Nived et al (2015)<br><br>Total N=24*<br>Mean age: 52y (22 - 80y)<br>**subgroup of total trial population, only (otherwise) immunocompetent participants included*	**Group 1 (PCV naïve*):** PCV13<br>**Group 2 (prior PCV13):** no vaccine, only cross sectional measurement.<br><br>*no prior PCV, no PPV ≤1 years, or ≥2 PS specific-IgG <0.35 µg/mL	**Group 1 (N=15)**<br>At baseline: GMCs were ≥0.35 µg/mL for PS 1,4, 6B, 7F, 9V, 14, 18C, 19A, 19F, and 23F.<br>After PCV13 at week 4-6: GMCs increased significantly for PS 4, 7F, 19F, 19A, 23F, 5, 9V, 14, 18C, 19A and slightly decreased for PS 6B. All GMCs were ≥0.35 µg/mL and all but PS 3 were ≥1.00 µg/mL<br><br>**Group 2 (N=9)**<br>Cross sectional: GMCs were ≥0.35 µg/mL for all PS, 86-100% had PS-specific IgG ≥0.35 µg/mL<br><br>**Conclusion**: PCV13 significantly increased a IgG response for most PCV13-PS, except for PS 6B.

Rosado et al (2013)<br><br>Total N=144<br>**Adults asplenia N=57** Median age: 35y (18.2-82.9y)<br>**Adults HCs=47**<br>Median age: 31.7y (21.5-80.5y)<br><br>**Children asplenia N=21**<br>Median age: 13.6y (1.2-18y)<br>**Children HCs=19**<br>Median age: 7.6y (2.8-16y)	No vaccination	In asplenic individuals (children and adults), the IgM memory B-cell count and secretion was significantly reduced compared to HCs.<br><br>In asplenic adults vaccinated with PPV23, the proportion of IgM (3.4% vs 16.1%) and switched (9% vs 16.1%) memory B-cells was severely and significantly reduced compared to HCs. Pneumococcal-specific IgM and IgG secreting cells were similarly reduced.<br><br>In asplenic children vaccinated with PPV23 before/after or PCV before splenectomy, anti-pneumococcal specific IgG memory B-cell count was reduced. When vaccinated with PCV after splenectomy, these cell counts were comparable to HCs and Pneumococcal-specific IgG was higher.<br><br>**Conclusion**: A reduced number of pneumococcal specific IgM memory B-cells was detected in asplenic patients compared to healthy controls. Pneumococcal IgG secreting cells were reduced or normal, varying by age and timing of vaccination.
Gazi et al (2017)<br><br>Total N= 19<br>**Asplenia N=14**<br>Mean age: 37.3y (20-53y)<br><br>**HCs N= 5**<br>Mean age: 36y (25-48y)	**All**: PCV13 + PPV23 (at week 0 and 8)<br><br>*0.5 mL subcutaneously administered*	The numbers of PCV13-specific memory B-cells were significantly lower in asplenic individuals at baseline compared to HCs and increased after PCV13. Levels did not further increase after PPV23.<br><br>**Conclusion**: Asplenic participants had lower levels of anti-PCV13 IgG producing memory B-cells at baseline compared to healthy controls and these cells were significantly increased by PCV13 similarly in patients and HCs.

Szenborn et al. (2017)<br><br>Total N= 52<br>Asplenia N=46<br>**Primed N=18**<br>Mean age=11.3y<br>**Unprimed=28**<br>Mean age=8.6y<br><br>HCs=6<br>Mean age: 2.8y<br>**Anticipated N was 100, complicated inclusion*	PCV10<br><br>Stratified in 3 age groups	Baseline: in the primed group 83-100% of GMCs were ≥0.2 µg/mL, in the unprimed group 11/12 tested GMCs were <0.2 µg/mL<br><br>One month after (last) PCV10<br>**Primed**: GMCs increased for al PCV10-PS and non PCV10 PS (PS6A and 19A). OPA GMTs did not increase for PS 6B, 14 and 23F. 100% of GMCs for all PCV10-PS and PS 6A and 94-95% of GMCs for PS 19A were ≥0.2 µg/mL. Also, 100% of OPA GMTs was ≥8<br><br>**HCs:** all GMCs were ≥0.2 after the 2nd PCV10, and OPA GMTs were all ≥8 except for PS1<br><br>**Conclusion**: PCV10 increased the IgG GMCs for all PCV10-PS and non-PCV10 (PS6A and 19A) and induced opsonophagocytic activity. OPA response was higher and broader after 2 doses compared to 1 dose of PCV10. No differences between patients and HCs apparent.
Takeshita et al (2021)<br><br>Total N =123<br>**Asplenia N=23**<br>Median age: 11y (0-22y)<br>Total HCs=100	No vaccination	**Vaccination history of asplenic patients**: 1 patient received PCV13, 1 received PCV13 and PPV23 <5 yrs ago, 1 patient received PPV23 <5 yrs ago, 12 patients received PPV23 ≥5 yrs ago, and 5 patients never received any vaccine.<br><br>Cross sectional measurement: GMCs were ≥0.2 µg/mL for all 6 tested PS, but significantly lower for PS 1, 3 and 6A compared to HCs<br><br>When never received pneumococcal vaccination: GMCs did not significantly differ from vaccinated individuals<br><br>When only received PPV23: GMCs were significantly lower for PS 1, 6A, 7F and 19A and OPA GMTs were significantly lower for PS 3, 5, and 19A, when compared to patients who received PCV13<br><br>**Conclusion**: complicated due to extensive heterogeneity regarding trial population and time since vaccination.

4b. Main outcomes: splenectomised with hemoglobinopathies (randomized and non-randomized studies)

Trial population	Vaccination schedule	Findings and conclusion

Stoehr et al (2005)<br><br>Total N=42<br>**Asplenia TS N=16**<br>Mean age =18y (7-26y)<br>**Asplenia PS N=23**<br>Mean age =13y (5-24y)<br><br>**HCs=3**<br>Mean age: 23y (14-25y)	**All**: PCV7+ PPV23<br>(at week 0 and 8)	Baseline: 46.3% of GMCs was not ≥1.0 µg/mL<br><br>At week 8 after PCV7: GMCs increased significantly for all PS, and 2-14% did not achieve GMCs ≥1.0 µg/mL<br>A week 12 (4 weeks after PPV23): GMCs increased significantly for PS 5 and 7 in most participants<br><br>No significant difference between patients and healthy controls was reported at any time point.<br><br>**Conclusion**: PCV7 significantly increased IgG response for all PCV7-PS and PPV23 broadened response for non-PCV7 PS.
Orthopoulos et al (2009)<br><br><br><br><br><br>**Total aplenia N=35**<br>Median age: 30y (12-41y)<br><br>**Total Controls* = 23**<br>Median age: 30 (13-40y)<br>**all male*	**Group 1:** PCV7 + PPV23 + PPV23<br>(at month 0, 1, and 12)<br>**Group 2:** PCV7 + PCV7 + PPV23<br>(at month 0, 1, and 12)<br>**HCs:** PCV7 + PPV23<br>(at month 0 and 1)	Baseline: GMCs were similar in both groups<br><br>After 1st dose (PCV7): a significant increase in GMCs for most PS was observed for both groups<br>After the 2nd dose (PCV or PPV23): only minor changes were observed, no differences between groups<br>At 12 months: GMCs significantly decreased, but after PPV23 booster GMCs increased equally in both groups, and kinetics of avidity were similar between groups<br><br>Participants with higher nr. of PPV23 doses had lower fold increases and for PS 9V and 19F hypo-responsiveness was observed.<br><br>**Conclusion**: PCV7 significantly increased IgG response for most PCV7-PS, minor contribution of a second dose of PCV7 or PPV23. After PPV23 booster no difference in both groups was observed. No difference between patients and healthy controls.

Papadatou et al (2014)<br>***Follow up Orthopoulos 2009**<br><br>Total N=39<br>Mean age: 36.5y (19-48y)	**All**: PCV13	Baseline: all had detectable IgM and IgG memory B-cells against at least one PS, all GMCs were ≥0.35 μg/mL<br><br>On day 7: IgG memory B-cells increased significantly for PS 9V, 19F, 23F and 3, and IgG GMCs increased significantly for PS 9V, 19F, 23F, and 19A<br>On day 28: IgG memory B-cells and IgG GMCs for all PS increased significantly compared to baseline<br><br>Nr. of previous PPV23s was negatively correlated with IgG GMC (PS23F) and IgM (PS 19F and 3) memory B-cell count and fold increase of GMC for PS 19F and 23F on day 28 after PCV13.<br><br>**Conclusion**: PCV13 induced a significant PS-specific IgG memory B-cell and IgG response. A higher nr. of PPV23 in the past negatively influenced both B-cell and IgG response.
Papadatou et al (2015)<br>***Follow up Orthopoulos 2009**<br><br>Total N=24<br>Mean age: 36y	**All**: PCV13	Baseline: all GMCs were ≥0.35μg/mL<br><br>At month 1: all GMCs increased significantly, but these were significantly lower than after PCV7, 7 years previously<br><br>No effect of age, sex, and vaccination history was detected. GMCs at baseline were negatively correlated with fold increase at month 1 for PS 9V, 19F, and 23F<br><br>**Conclusion**: PCV13 induced a significant PS-specific IgG response, and higher baseline GMCs negatively influenced post vaccination IgG GMCs.
Rezai et al (2016)<br><br>Total N=47<br>Mean age: 29.6y (20-44y)	**Group 1:** PCV13 + PPV23<br>**Group 2:** PPV23 + PCV13<br>(at month 0 and 2)	After the 2nd vaccine (PCV13 or PPV23): GMCs were significantly increased in both groups, and GMCs were significantly higher when PCV was administered first<br><br>**Conclusion**: PCV23 prior to PCV13 negatively influenced anti-pneumococcal IgG GMCs.

Sari et al (2017)<br><br>Total N=56<br>Median age: 21y (12-38y)<br><br>50 participants received also PPV23, according to local guidelines.	**Group 1**: PCV13 + placebo*<br>**Group 2**: PCV13 + zinc*<br><br>*zinc/placebo liquid daily for 4 weeks after each vaccine<br><br>PPV23 at 8 weeks was indicated when never received PPV23 before.	Baseline: GMCs were >250mU/mL in both groups<br><br>At week 12 after PCV13: a significant increase in anti-pneumococcal GMC was observed in all patients and all anti-pneumococcal IgG levels were >250 mU/mL<br><br>**Conclusion**: PCV13 significantly increased total anti-pneumococcal IgG response. Zinc supplementation did not affect serological response after PCV13.
Papadatou et al (2019)<br>***Follow up Papadatou 2014/2015**<br><br>Total N=34<br>Age range= 24-53y	No vaccination	5 years after PCV13: All GMCs were >1.0 µg/mL except for PS 3 (0.93 µg/mL). GMCs decreased significantly compared to day 28 after PCV13 remaining above baseline for PS 9V, 19A and 19F, dropping to baseline for PS 23F and dropping below baseline for PS 3<br><br>GMCs 5 years after PCV13 were positively correlated with GMCs at day 28 after PCV13. The memory B-cell response and number of previous PPV23s were not correlated.<br><br>**Conclusion**: 5 years after PCV13 IgG GMCs significantly decreased, and participants with a higher initial peak response had higher GMCs 5 years after vaccination.

Table 4c. Main outcomes: individuals with sickle cell disease (randomized and non-randomized studies)

Trial population	Vaccination schedule	Findings and conclusion

Vernacchio et al (1998)<br><br>Total N=23<br>**Group 1 N= 11**<br>Median age: 14y (4-30y)<br>**Group 2 N=12**<br>Median age: 13y (4-27y)	**Group 1**: PCV7 + PCV7 + PPV23<br>(at week 0, 8, and 16)<br>**Group 2:** PPV23<br><br>Concomitant with Hib and meningococcal ACWY vaccine	Baseline: no differences between groups were detected, all GMCs were ≥0.35 µg/mL<br><br>After 1st PCV7: GMCs increased significantly for all PS except 6B<br>After 2nd PCV7: GMCs only significantly increased for PS 6B + 14<br><br>After PPV23: GMCs were significantly higher for PS14 and PS19F in group 1. Fold increases ranged from 4.1-22.3 and 2.2-5.4 for group 1 and 2, respectively. GMCs were all ≥1.0 µg/mL<br><br>**Conclusion**: PCV7 significantly increased IgG GMCs for PCV7-PS with a minor contribution after a 2nd PCV7. PCV7 induced a significantly higher response compared to PPV23.
Vernacchio et al (2000)<br><br>Total N=23<br>**Group 1 N= 11**<br>Median age: 14y (4-30y)<br>**Group 2 N=12**<br>Median age: 13y (4-27y)	**Group 1**: PCV7 + PCV7 + PPV23<br>(at week 0, 8, and 16)<br>**Group 2:** PPV23<br><br>Concomitant with Hib and meningococcal ACWY vaccine	Baseline: no differences in OPA GMTs between groups<br><br>After PPV23: OPA GMTs were significantly higher for 5/7 PS (6B, 14, 18C, 19F, and 23F) in group 1 compared to group 2. Notably, IgG GMCs were only significantly higher for 2/7 PS (PS14 and PS19F). Fold increases of OPA GMTs ranged from 64-226 and 1.5-38.1 in group 1 and 2, respectively.<br><br>A moderate significant correlation was found between the IgG and OPA response<br><br>**Conclusion**: PCV7+PPV23 induced a PS-specific OPA response which was broader and higher than after PPV23. The IgG response differed less extensively between groups. IgG and OPA we not completely concordant.

Nowak-Wegrzyn et al. (2000)<br><br><u>Total N=48</u><br>**<u>Group 1</u>**<br>**SCD N=28**<br>**HCs=7**<br>All 2 months of age<br><br>**<u>Group 2</u>**<br>**SCD N=10**<br>**HCs=3**<br>All 10 months of age	**Group 1**: PCV7 + PCV7 + PCV7 + PPV23 (at age 2, 4, 6, and 24m)<br>**Group 2**: PCV7 +PPV23 (at age 12 and 24m)	**Group 1:**<br><u>Baseline:</u> for both PS GMCs were <0.35 µg/mL<br><u>After primary series (month 7)</u>: OPA activity for PS 6B increased significantly from 4.8% to 33.5% for children with SCD<br><u>At month 12:</u> OPA activity decreased to 8.1% (PS 6B) for children with SCD<br><u>At month 24:</u> OPA activity decreased to 7.5% (PS 6B) for children with SCD<br><u>After PPV23 at month 25:</u> OPA activity increased significantly to 30.7% for PS 6B and to 27.3% for PS14, for children with SCD.<br><br>No difference was found between children with SCD and healthy controls.<br><br>**Group 2:**<br><u>After PCV:</u> OPA activity increased significantly from 4.3% to 14.4% for children with SCD<br><u>At month 24</u>: OPA activity decreased to 4.6% (for PS 6B)<br><u>After PPV23 at month 25:</u> OPA activity significantly increased to 35% for PS 6B for children with SCD.<br><br>GMCs correlated significantly but not strongly with serum OPA activity.<br><br>A comparable was observed for PS 6B and PS14.<br><br>**<u>Conclusion</u>**: PCV7 induces opsonophagocytic activity for PS 6B and 14 in children with SCD similarly to healthy controls. Primary levels decreased and were boosted by PPV23.

O'Brien et al. (2000)<br><br>Total N=61<br>**Schedule A N=34**<br>Mean age: 2.3m<br>**Schedule A HCs=11**<br>Mean age: 2.1m<br><br>**Schedule B N=13**<br>Mean age: 12.3m<br>**Schedule B HCs=3**<br>Mean age: 13.4m	**Group 1 (<2 months)**: PCV7+ PCV7 + PCV7 + PPV23 (at age 2, 4, 6, and 24)<br>**Group 2 (2-12 months)**: PCV7 + PPV23 (at age 12 and 24m)	**Schedule A (starting at 2 months of age)**:<br>Baseline: GMCs were <0.35 μg/mL for all PS in children with SCD and for 3/7 PS for HCs<br>After primary series at month 7: all GMCs significantly increased and were ≥1.0 μg/mL, the GMCs of children with SCD were comparable to HCs, except for PS 18C and 19F<br>At 12 months: GMCs remained significantly higher than baseline for all PS except PS 14<br>At 24 months: GMCs significantly decreased, 56-100% of GMCs ≥ 1.0 μg/mL<br>After PCV23 at 25 months: GMCs for all PS significantly increased, and were ≥1.0 μg/mL<br><br>**Schedule B (starting at 12 months of age):**<br>Baseline: for all participants, all GMCs were <0.35 μg/mL<br>After PCV7: GMCs for all PS significantly increased<br>At 24 months: GMCs significantly decreased for PS 9V, 14, and 18C, with 31-71% of GMCs ≥ 1.0 μg/mL<br>After PPV23 at 25 months: GMCs increased for all PS, and 32/33 participants had GMCs of ≥1.0 μg/mL for all PS<br><br>Similar trends were observed in SCD and HCs.<br><br>For PS 14, high GMCs at baseline led to lower (but ≥1.0 μg/mL) GMCs post vaccination.<br><br>**Conclusion**: PCV7 (1 and 3 doses) significantly increased IgG GMCs for PCV7-PS, which decreased and were boosted with PPV23. No differences between patients and healthy controls.
Reinert et al. (2007)<br><br>Total N=51*<br>All started at 2 m of age<br>**49 were vaccinated*	**All**: PCV7 + PCV7 + PCV7 + PPV23 (at month 2, 3, 4, 15-18).	Baseline: GMCs were <0.35 μg/mL for 5/7 PS<br>After primary series at month 5: 95.6% had IgG levels ≥0.35 μg/mL for all PCV7-PS and 32/34 had OPA titers ≥8 for 4/4 tested PS<br>Prior to PPV23 (15-18m): GMCs decreased and remained slightly above baseline<br>After PPV23 (15-18 + 1m): GMCs for all PS increased and 90% was ≥1.0 μg/mL<br><br>**Conclusion**: 3 doses of PCV7 increased significantly IgG GMCs for PCV7-PS, which declined and were significantly boosted with PPV23.

Rivera-Olivero et al. (2014)<br><br>Total N=22*<br>**subset (children with SCD) of the total trial population of 82 of children with increased risk for IPD*	**2-6 m of age:** PCV7 + PCV7 + PCV7<br>**7-23 m of age:** PCV7 + PCV7<br>**≥24 m of age:**<br>**- previous PCV7:** one PCV7<br>**-no previous PCV7:** PCV7 + PCV7<br>*All groups: interval of 6-8 weeks*	At baseline: GMCs were ≥0.35μg/mL for all PS and ≥1.0 μg/mL for 3/7 PS<br><br>At week 8 after last PCV7: GMCs were ≥1.0 μg/mL for all PS, colonization with PS 6B resulted in significantly lower post vaccine anti-PS 6B GMCs<br><br>**Conclusion**: PCV7 (1, 2, or 3 doses) increased IgG GMCs for PCV7-PS. Colonization might affect IgG response for the same PS after vaccination.
De Montalembert et al. (2015)<br><br>Total N=158<br>Mean age: 13.3y	**All**: PCV13 + PCV13 (at month 0 and 6)	At baseline: IgG GMCs for PCV13-PS were all ≥1.0 μg/mL, except for PS 4<br><br>After 1st PCV13: IgG GMCs and OPA GMTs significantly increased<br>Month 6: IgG GMCs and OPA GMTs decreased<br><br>After 2nd PCV13 (N=146): GMCs and OPA GMTs increased significantly, but the GMCs and fold change were significantly lower than after 1st dose, OPA GMTs were similar for 3 PS and higher for PS 1, 7F, 9V, and 23F than after 1st dose<br>1 year after 2nd PCV13 (n=87): GMCs were significantly higher compared to baseline except for PS 3 and 5. OPA GMTs remained significantly higher compared to baseline for all PS<br><br>**Conclusion**: PCV13 significantly increased IgG and OPA response for PCV13-PS, the 2nd dose after 6 months significantly increased OPA GMTs and, to lesser extent, IgG GMCs.
Disu et al. (2016)<br><br>Total N=200<br>Patients N=151<br>**Mean age: 38.91 m**<br>Age range: 5-60 m<br>HCs=49<br>**Mean age: 16.39 m**<br>Age range: 4-60 m	**All:** PCV13	At baseline: 2/3 of participants had GMCs ≥200 μg/mL, children with SCD had higher anti-pneumococcal IgG baseline GMCs compared to HCs<br><br>After PCV13 at month 2: GMCs significantly increased in children with SCD and HCs, significant more HCs reached a ≥4 fold increase<br><br>**Conclusion**: PCV13 significantly increased anti-pneumococcal IgG GMCs in children with SCD. More healthy controls reached a 4 fold increase (however, patients had higher baseline GMCs)

Sirima et al (2017)<br><br>Total N=300 (each group N=50)<br>**<6 months SCD**<br>Mean age: 8.4w<br>**<6 months HC**<br>Mean age: 8.6w<br>**7-11 months SCD**<br>Mean age: 8.6w<br>**7-11 months HC**<br>Mean age: 8.3w<br>**12-23 months SCD**<br>Mean age: 16.6w<br>**12-23 months HCs**<br>Mean age: 16.7w	6 groups (SCD and HCs):<br>**<6 months SCD**: PCV10 + PCV10 + PCV10 + PCV10 (at week 8-11, 12, 16, and month 9-10)<br>**Aged 7-11 months**: PCV10 + PCV10 + PCV10 (at month 7-11, 8-12, and 10-14)<br>**Aged 12-23 months**: PCV10 +PCV10 (at month 12-23 and 14-25)	At baseline: most GMCs were <0.2 μg/mL<br><br>**Starting at <6 months of age**<br>After primary series + 1 month: 97.8% of GMCs ≥0.2 μg/mL for all PS except PS 6B and 23F, ≥93.2% of children with SCD and 89.7% of HCs had OPA titers ≥8 for all PS except PS 1, 6B and 23F<br>At booster + 1 month: 100% of GMCs ≥0.2 μg/mL for all PS except PS 14 and 23F, and 97.5% of children with SCD and 93.9% of HCs had OPA titers ≥8<br><br>**Starting at 7-11 months of age**<br>After primary series + 1 month: 98% of children with SCD and 93.9% of HCs had PS-specific IgG of ≥0.2 μg/mL for all PS except PS 6B and 23F, 87.5% of children with SCD and 84.4% of HCs had OPA GMTs of ≥8<br>At booster + 1 month: 89.8% of children with SCD and 91.8% of HCs had GMCs ≥0.2 μg/mL, and 89.4% of children with SCD and 95.1% of HCs had OPA GMTs ≥8<br><br>**Starting at 12-23 months of age**<br>After two doses + 1 month: 91.7% of children with SCD and HCs had PS-specific IgG levels ≥0.2 μg/mL and 88.6% had OPA titers ≥8 against all except PS 1<br><br>No significant differences between children with SCD and healthy controls.<br><br>**Conclusion**: PCV10 (4, 3, or 2 doses) significantly increased IgG and OPA response against most PCV10-PS and PS 6A and 19A (non-PCV10-PS). No differences between children with SCD and healthy controls.
Melica et al. (2023)<br><br>Total N=128<br>Mean age: 38.9y (SD 10.4)<br>**Group 1 N=63**<br>Mean age: 39.2y<br>**Group 2 N=65**<br>Mean age: 38.7y	**Group 1**: PCV13 + PCV23 (at month 0, 1)<br>**Group 2**: PPV23 (month 1)	Baseline: GMCs were ≥0.35 μg/mL for 10/13 PS in group 1 and for 8/13 PS in group 2<br><br>At week 8: group 1 had significantly higher response rate compared to group 2, with 24.6% vs 8.2% showing a ≥4 fold increase in GMCs for ≥10 PS, respectively. For OPA GMTs, these numbers were 36.2% vs 8.7%, respectively. Also, GMCs were significantly higher in group 1 for 7/13 shared PS and for 10/13 PS in the OPA GMTs<br>At week 96: GMCs were higher in group 1 compared to group 2 for 5/13 shared PS<br><br>**Conclusion**: PCV13 followed by PPV23 induced higher and broader PCV13-PS specific IgG response as compared to solely PPV23.

Quinn et al (2023)<br><br>Total N=103<br>**Group 1 N=69** Mean age: 10.8y (SD 3.5)<br>**Group 2 N=34** Mean age: 10.8y (SD 3.3)	**Group 1:** PCV15<br>**Group 2:** PCV13	Baseline: GMCs were ≥0.35 μg/mL for 12/14 PS in both groups, and ≥1.00 μg/mL for 4/14 PS in group 1 and 10/12 PS in group 2<br><br>At day 30: GMCs and OPA GMTs were comparable between groups for shared PS, and higher in group 1 for PCV15-PS (PS 22F and 33F)<br><br>**Conclusion**: PCV15 induced a comparable increase in IgG and OPA response compared to PCV13 for shared PS and broadened the response to PCV15 unique PS.

Abbreviations: N= trial population size, y=years, m=months, GMC= Geometric Mean Concentration (always IgG), PS= pneumococcal serotype, PCV7/10/13/15= 7-valent/10-valent/ 13-valent/ 15-valent pneumococcal conjugate vaccine, PPV23= 23 valent pneumococcal polysaccharide vaccine, PCV7/10/13/15-PS= pneumococcal serotypes contained in PCV7/10/13/15, HCs=healthy controls, non-PCV7/10/13/15-PS= pneumococcal serotypes not included in PCV7/PCV10/PCV13/PCV15, vs=versus, INF-y= interferon- y, Th1= T 1 helper cell, OPA= opsonophagocytic assay, GMT= Geometric Mean Titer, Nr. = number, Hib= *Haemophilus influenzae type b*, SCD= sickle cell disease, m=months, IPD= Invasive Pneumococcal Disease, TS= Total Splenectomy, PS= Partial Splenectomy, SD= standard deviation

Table S3. Immunological outcomes per study (<0.35 indicated in red (IgG), <8 indicated in red (OPA), 0.35-1.00 indicated in orange (IgG))

1a. Studies assessing splenectomised patients with mixed comorbidity

Study	Endpoint	Timepoint	Anti-pneu	4	6B	9V	14	18C	19F	23F	1	3	5	6A	7F	19A
Smets (2007)	ELISA PS-specific GMCs in ug/mL	Month 1:														
		Group PPV23	-	75%	25%	62%	43%	43%	33%	43%	-	-	-	-	-	-
		Group PCV7	-	64%	82%	55%	50%	78%	22%	70%	-	-	-	-	-	-
		Month 6:														
	**≥4 fold %**	Group PPV23	-	62%	25%	62%	29%	43%	50%	29%	-	-	-	-	-	-
		Group PCV7	-	70%	50%	30%	33%	89%	25%	56%	-	-	-	-	-	-
Mikoluc (2008)	ELISA PS-specific GMCs in ug/mL	Baseline	-	0.33	0.21	0.63	1.37	0.73	1.45	0.63	-	-	-	-	-	-
		Week 8	-	3.07	1.52	3.23	10.05	3.85	4.38	5.01	-	-	-	-	-	-
		%≥2 fold	-	*83%*	*77%*	*84%*	*64%*	*67%*	*57%*	*80%*	-	-	-	-	-	-
Stanford (2009)	ELISA PS-specific IgG GMC in ug/mL	**All participants**														
		Baseline	-	1.10	1.67	1.48	8.18	3.75	3.11	1.78	1.27	-	2.03	-	-	-
		Week 3-6	-	2.68	3.96	3.30	13.88	7.68	4.73	6.61	1.25	-	1.96	-	-	-
		≥ 2 fold rise (%)	-	*52%*	*44%*	*43%*	*27%*	*43%*	*32%*	*69%*	*9%*	-	*8%*	-	-	-
		≥ 0.35 (%)		*91%*	*90%*	*94%*	*99%*	*97%*	*97%*	*97%*	*90%*	-	*92%*	-	-	-
		≥ 1.00 (%)		*74%*	*76%*	*78%*	*95%*	*95%*	*88%*	*87%*	*61%*	-	*66%*	-	-	-
		**After PPV23**														
		**(N=31)**	-	1.65	2.08	2.16	7.55	4.70	2.96	4.23	0.98	-	2.19	-	-	-
		Baseline (M6)	-	2.78	2.79	3.31	12.76	6.04	6.16	4.58	1.99	-	3.66	-	-	-
		M6+3-6 weeks	-	*32%*	*23%*	*19%*	*29%*	*19%*	*55%*	*13%*	*48%*	-	*42%*	-	-	-
		≥2 fold rise (%)	-	*94%*	*97%*	*94%*	*100%*	*97%*	*100%*	*97%*	*88%*	-	*97%*	-	-	-
		≥ 0.35 (%)	-	*76%*	*79%*	*82%*	*94%*	*94%*	*94%*	*88%*	*64%*	-	*88%*	-	-	-
		≥ 1.00 (%)														
Meerveld-Eggink (2011)	Luminex PS-specific IgG GMCs in ug/mL	Baseline		0.6	1.1	1.3	6.2	1.9	2.6	1.3	-	-	-	-	-	-
		Week 3		1.7	3.0	4.8	12.7	5.6	4.7	6.8	-	-	-	-	-	-
		Week 9		2	4.5	4.7	12.2	5.6	5.4	9.1	-	-	-	-	-	-
		Month 6+3 wk		2.1	3.8	4.1	14.7	4.1	5.9	10.7	-	-	-	-	-	-
Nived (2015)	Luminex assay GMC in ug/mL	**No vaccination**														
		Cross sectional	-	-	-	-	-	-	-	-	-	-	-	-	-	-
		**PCV13***														
		Baseline		0.51	2.07	2.14	6.21	0.71	3.57	2.68	5.54	0.28	0.24	-	1.69	5.60
		Week 4-6		1.48	1.70	2.68	8.39	2.07	5.96	8.62	9.29	0.53	1.10	-	4.60	9.19

		*only immuno-competent considered here														
Gazi (2017)	ELISpot,	Baseline	-	-	-	-	-	-	-	-	-	-	-	-	-	-
	Nr of spots	Week 8	-	-	-	-	-	-	-	-	-	-	-	-	-	-
		Week 12	-	-	-	-	-	-	-	-	-	-	-	-	-	-
Szenborn	22F-ELISA	**Primed group**														
(2017)	PS-specific	Baseline	-	0.75	1.24	1.25	8.26	1.19	2.79	1.36	0.60	-	0.73	1.99	0.99	1.75
	GMC in	≥0.2 (%)	-	88.9%	94.4%	83.3%	100%	88.9%	100%	88.9%	88.9%	-	83.3%	100%	94.4%	94.4%
	ug/mL	Month 1	-	6.16	3.66	2.87	21.08	10.84	18.22	2.24	1.94	-	2.44	3.76	3.34	4.44
		≥0.2 (%)	-	100%	100%	100%	100%	100%	100%	100%	100%	-	100%	100%	100%	94.4%
	Multiplex															
	OPA assay	**Unprimed group**														
	PS-	Baseline	-	0.13	0.17	0.20	1.00	0.22	0.84	0.15	0.14	-	0.15	0.29	0.21	0.30
	specific	≥0.2 (%)	-	26.1%	39.1%	43.5%	82.6%	39.1%	73.9%	39.1%	39.1%	-	43.5%	60.9%	56.5%	56.5%
	OPA titer	Month 1	-	9.26	1.21	1.89	7.78	13.04	12.41	1.09	1.52	-	2.74	1.02	3.25	2.18
		≥0.2 (%)	-	100%	95.7%	100%	100%	100%	95.7%	87%	100%	-	100%	87%	100%	87%
		Month 3	-	7.67	2.19	3.23	13.64	24.15	20.53	2.26	2.52	-	3.93	1.50	5.60	4.05
		≥0.2 (%)	-	100%	100%	100%	100%	100%	100%	100%	100%	-	100%	100%	100%	95.2%
		OPA titer														
		**Primed group**														
		Baseline	-	240.4	1147.9	812.2	2639	256.9	96.7	1843.2	8.5	-	13.2	496.9	1992.5	176.3
		≥8 (%)	-	73.3%	93.3%	92.3%	100%	76.9%	66.7%	100%	20%	-	35.7%	80%	100%	78.6%
		Month 1	-	1291	698.1	1955.3	2495.7	4421.4	3272.4	613.8	12.4	-	75.9	2590.9	2787.4	961.2
		≥8 (%)	-	100%	%	100%	100%	100%	100%	93.3%	42.9%	-	93.3%	100%	100%	93.3%
					100%											
		**Unprimed group**														
		Baseline	-	6.6		161	298.1	13.5	45.9	70	4.0	-	4.8	51	615.9	19.5
		≥8 (%)	-	10.5%	132.4	68.4%	76.5%	27.8%	52.6%	50%	0%	-	11.1%	47.1%	83.3%	35.3%
		Month 1	-	1497	61.1%	1076.5	2724.6	8665.8	4569.1	1326.4	8.6	-	112.5	1982.6	4433.6	1037.4
		≥8 (%)	-	100%	1188.8	88.2%	100%	100%	100%	100%	28.6%	-	93.8%	93.8%	100%	93.8%
		Month 3	-	2657	100%	1319.5	6590.5	9939.8	3441.8	1949.3	29.1	-	212.9	1908.3	6724.3	2769.3
		≥8 (%)	-	100%	2384.3	100%	100%	100%	100%	100%	66.7%	-	100%	100%	100%	100%
					100%											
Takeshita	22F-ELISA	**Patient**														
(2021)	PS-specific	**participants**	-	-	-	-	-	-	-	-	0.78	0.31	1.57	1.00	1.07	1.91
	GMC in	All	-	-	-	-	-	-	-	-	100%	70%	100%	96%	96%	100%
	ug/mL	≥0.2 (%)	-	-	-	-	-	-	-	-	0.60	0.26	1.25	0.76	0.65	1.26

		PPV23 only	-	-	-	-	-	-	-	-	*100%*	*69.2%*	*100%*	*92.3%*	*92.3%*	*100%*
	Multiplexed	≥0.2 (%)	-	-	-	-	-	-	-	-	0.87	0.32	1.96	1.39	1.66	2.7
	opsonopha	No vaccine	-	-	-	-	-	-	-	-	*100%*	*60%*	*100%*	*100%*	*100%*	*100%*
	gocytic	≥0.2 (%)														
	killing															
	assay		-	-	-	-	-	-	-	-	1.5	1.38	1.95	2.07	0.89	2.66
	(MOPA)	**Controls:**	-	-	-	-	-	-	-	-	*100%*	*100%*	*100%*	*100%*	95%	*100%*
	PS-specific	≥0.2 ug (%)														
	OPA titer															
		**OPA:**	-	-	-	-	-	-	-	-	-	50	11	-	-	281
		All	-	-	-	-	-	-	-	-	-	*91%*	*48%*	-	-	*87%*
		≥8 (%)	-	-	-	-	-	-	-	-	-	39	7	-	-	111
		PPV23 only	-	-	-	-	-	-	-	-	-	*92%*	*31%*	-	-	*77%*
		≥8 (%)	-	-	-	-	-	-	-	-	-	31	9	-	-	450
		No vaccine	-	-	-	-	-	-	-	-	-	*89%*	*40%*	-	-	*100%*
		≥8 (%)														

1b. Studies assessing splenectomised patients with hemoglobinopathies

Study	Endpoint	Timepoint	Anti-pneu	4	6B	9V	14	18C	19F	23F	1	3	5	6A	7F	19A
Stoehr (2005)	PS-specific GMCs in ug/mL, assay not described.<br><br>Exact titers are not shown.	Baseline<br>Week 8<br>Week 12	-<br>-<br>-	-<br>-<br>-	-<br>-<br>-	-<br>-<br>-	-<br>-<br>-	-<br>-<br>-	-<br>-<br>-	-<br>-<br>-	-<br>-<br>-	-<br>-<br>-	-<br>-<br>-	-<br>-<br>-	-<br>-<br>-	-<br>-<br>-
Orthopoulos (2009)	ELISA PS-specific IgG GMCs.<br><br>Avidity Index	**Group A**														
		Baseline	-	-	6.3	5.7	12.8	-	9.4	4.5						
		Month 1	-	-	22.3	9.3	17.2	-	14.4	14.8						
		Month 2	-	-	26.2	12.4	16.2	-	16.5	13.1						
		Month 12	-	-	10.6	8.2	11.6	-	5.5	6.2						
		Month 13	-	-	17.2	9.2	16.7	-	7.4	6.7						
		**Group B**														
		Baseline	-	-	4.5	5.9	10.2	-	9.4	5.6						
		Month 1	-	-	15.9	11.0	16.9	-	14.4	19.0						
		Month 2	-	-	18.8	12.6	22.2	-	10.2	23.7						
		Month 12	-	-	8.8	6.4	15.2	-	2.8	10.8						

		Month 13<br><br>**Controls:**<br>Data not shown	-	-	14.5	7.8	19.2	-	9.2	11.8						
Papadatou (2014)	ELISpot PS specific IgM and IgG Memory B cell counts, GMC in ug/mL	**IgG**<br>Baseline*<br>Day 7<br>Day 28<br><br>*Exact titers not shown	 -<br>-<br>-	 -<br>-<br>-	 -<br>-<br>-	 >0.35<br>-<br>7.76	 -<br>-<br>-	 -<br>-<br>-	 >0.35<br>-<br>18.42	 >0.35<br>-<br>17.47	 -<br>-<br>-	 >0.35<br>-<br>4.02	 -<br>-<br>-	 -<br>-<br>-	 -<br>-<br>-	 >0.35<br>-<br>15.01
Papadatou (2015)	ELISA PS specific GMC in ug/mL	Baseline<br>Month 1	-<br>-	-<br>-	-<br>-	2.16<br>5.12	-<br>-	-<br>-	6.98<br>11.86	4.52<br>9.62	-<br>-	-<br>-	-<br>-	-<br>-	-<br>-	-<br>-
Rezai (2016)	ELISA pneumococcal-specific IgG in ug/mL	**PCV+PPV**<br>Baseline<br>Week 8<br>Week 16<br><br>**PPV+PCV**<br>Baseline<br>Week 8<br>Week 16	 114.5<br>844<br>1049<br><br><br>115<br>1089.8<br>1497.3	 -<br>-<br>-<br><br><br>-<br>-<br>-	 -<br>-<br>-<br><br><br>-<br>-<br>-	 -<br>-<br>-<br><br><br>-<br>-<br>-	 -<br>-<br>-<br><br><br>-<br>-<br>-	 -<br>-<br>-<br><br><br>-<br>-<br>-	 -<br>-<br>-<br><br><br>-<br>-<br>-	 -<br>-<br>-<br><br><br>-<br>-<br>-	 -<br>-<br>-<br><br><br>-<br>-<br>-	 -<br>-<br>-<br><br><br>-<br>-<br>-	 -<br>-<br>-<br><br><br>-<br>-<br>-	 -<br>-<br>-<br><br><br>-<br>-<br>-	 -<br>-<br>-<br><br><br>-<br>-<br>-	 -<br>-<br>-<br><br><br>-<br>-<br>-
Sari (2017)	Pneumococcus IgG Immunopotency level/E-DG-MZ-001/04-04 kit in mU/mL.	**Zinc +**<br>PCV13(+PPV23*)<br>Baseline<br><br>All groups<br>Baseline<br>Week 12<br><br>**Zinc -**<br>PCV13(+PPV23**)<br>Baseline<br><br>All groups<br>Baseline<br>Week 12	 <br>1333<br><br><br>325<br>1812.5<br><br><br><br>880<br><br><br>338.5<br>1857.5	 <br>-<br><br><br>-<br>-<br><br><br><br>-<br><br><br>-<br>-	 <br>-<br><br><br>-<br>-<br><br><br><br>-<br><br><br>-<br>-	 <br>-<br><br><br>-<br>-<br><br><br><br>-<br><br><br>-<br>-	 <br>-<br><br><br>-<br>-<br><br><br><br>-<br><br><br>-<br>-	 <br>-<br><br><br>-<br>-<br><br><br><br>-<br><br><br>-<br>-	 <br>-<br><br><br>-<br>-<br><br><br><br>-<br><br><br>-<br>-	 <br>-<br><br><br>-<br>-<br><br><br><br>-<br><br><br>-<br>-	 <br>-<br><br><br>-<br>-<br><br><br><br>-<br><br><br>-<br>-	 <br>-<br><br><br>-<br>-<br><br><br><br>-<br><br><br>-<br>-	 <br>-<br><br><br>-<br>-<br><br><br><br>-<br><br><br>-<br>-	 <br>-<br><br><br>-<br>-<br><br><br><br>-<br><br><br>-<br>-	 <br>-<br><br><br>-<br>-<br><br><br><br>-<br><br><br>-<br>-	 <br>-<br><br><br>-<br>-<br><br><br><br>-<br><br><br>-<br>-

		*previous PPV23 (N=4)<br>** previous PPV23 (N=2)														
Papadatou (2019) N=34	ELISA GMCs in Ug/mL	28 days<br>5 years		-	-	7.76<br>2.76	-	-	18.42<br>8.53	17.47<br>8.75	-	4.02<br>0.93	-	-	-	15.01<br>12.15
**1b. Studies assessing patients with sickle cell disease**																
**Study**	**Endpoint**	**Timepoint**	**Anti-pneu**	**4**	**6B**	**9V**	**14**	**18C**	**19F**	**23F**	**1**	**3**	**5**	**6A**	**7F**	**19A**
Vernacchio (1998)	ELISA PS-specific IgG GMCs in ug/mL	**Group PCV+PPV**<br>Baseline<br>Week 3-6<br>Week 11-14<br>Week 19-22<br><br>**Group PPV**<br>Baseline<br>Week 3-6	 -<br>-<br>-<br>-<br><br><br>-<br>-	 0.40<br>2.23<br>2.24<br>2.94<br><br><br>0.59<br>2.39	 0.36<br>1.23<br>3.31<br>4.12<br><br><br>0.68<br>2.45	 1.56<br>5.08<br>4.69<br>7.63<br><br><br>0.80<br>3.96	 0.98<br>23.3<br>14.1<br>21.8<br><br><br>1.23<br>5.16	 0.75<br>2.84<br>3.99<br>3.08<br><br><br>0.54<br>2.92	 2.16<br>11.5<br>13.8<br>16.7<br><br><br>1.88<br>4.10	 0.59<br>5.40<br>5.53<br>5.99<br><br><br>0.67<br>2.08	 0.69<br>-<br>-<br>6.49<br><br><br>0.94<br>6.33	 -<br>-<br>-<br>-<br><br><br>-<br>-	 -<br>-<br>-<br>-<br><br><br>-<br>-	 -<br>-<br>-<br>-<br><br><br>-<br>-	 -<br>-<br>-<br>-<br><br><br>-<br>-	 -<br>-<br>-<br>-<br><br><br>-<br>-
Vernacchio (2000)	Flowcytometry OPA assay PS-specific OPA titers	**Group PCV+PPV**<br>Baseline<br>Week 19-22<br><br>**Group PPV**<br>Baseline<br>Week 3-6	 -<br>-<br><br><br>-<br>-	 -<br>-<br><br><br>-<br>-	 -<br>659<br><br><br>-<br>42.7	 -<br>-<br><br><br>-<br>-	 -<br>1810<br><br><br>-<br>215	 -<br>512<br><br><br>-<br>12.7	 -<br>290<br><br><br>-<br>30.2	 -<br>659<br><br><br>-<br>6.00	 -<br>-<br><br><br>-<br>-	 -<br>-<br><br><br>-<br>-	 -<br>-<br><br><br>-<br>-	 -<br>-<br><br><br>-<br>-	 -<br>-<br><br><br>-<br>-	 -<br>-<br><br><br>-<br>-
Nowak-Wegrzyn (2000)	ELISA PS specific IgG GMCs in ug/mL<br><br>OPA assay using heat inactivation, OPA activity in %	**Group 1 (2M)**<br>**IgG**<br>Baseline<br>7 months<br>12 months<br>24 months<br>25 months<br><br>**OPA activity**<br>Baseline<br>7 months	 <br>-<br>-<br>-<br>-<br>-<br><br><br><br>-	 <br>-<br>-<br>-<br>-<br>-<br><br><br><br>-	 <br>0.11<br>4.11<br>1.37<br>1.61<br>25.49<br><br><br>4.8%<br>33.5%	 <br>-<br>-<br>-<br>-<br>-<br><br><br><br>-	 <br>0.18<br>6.09<br>3.86<br>1.91<br>19.71<br><br><br>9.4%<br>24.9%	 <br>-<br>-<br>-<br>-<br>-<br><br><br><br>-	 <br>-<br>-<br>-<br>-<br>-<br><br><br><br>-	 <br>-<br>-<br>-<br>-<br>-<br><br><br><br>-	 <br>-<br>-<br>-<br>-<br>-<br><br><br><br>-	 <br>-<br>-<br>-<br>-<br>-<br><br><br><br>-	 <br>-<br>-<br>-<br>-<br>-<br><br><br><br>-	 <br>-<br>-<br>-<br>-<br>-<br><br><br><br>-	 <br>-<br>-<br>-<br>-<br>-<br><br><br><br>-	 <br>-<br>-<br>-<br>-<br>-<br><br><br><br>-

		12 months	-	-	8.1%	-	16.5%	-	-	-	-	-	-	-	-	-
		24 months	-	-	7.5%	-	12.6%	-	-	-	-	-	-	-	-	-
		25 months	-	-	30.7%	-	27.3%	-	-	-	-	-	-	-	-	-
			-	-		-		-	-	-	-	-	-	-	-	-
		**Group 2 (12M)**														
		Data not shown														
		N=10, HCS=3														
O'Brien (2000)	ELISA PS specific IgG GMCs	**Schedule A**														
		**SCD**														
		Baseline	-	0.05	0.10	0.13	0.20	0.13	0.23	0.12	-	-	-	-	-	-
		Month 7	-	2.67	4.03	2.26	5.93	3.10	6.08	5.2	-	-	-	-	-	-
		Month 12	-	0.84	1.30	0.84	3.83	0.96	2.54	1.07	-	-	-	-	-	-
		Month 24	-	0.68	1.63	0.46	1.91	0.65	2.59	0.85	-	-	-	-	-	-
		Month 25	-	15.04	24.64	9.29	18.02	16.33	44.19	13.77	-	-	-	-	-	-
		**HCs**														
		Baseline	-	0.15	0.38	0.44	0.51	0.24	0.79	0.31	-	-	-	-	-	-
		Month 7	-	1.80	2.92	1.18	4.71	1.30	2.04	2.16	-	-	-	-	-	-
		Month 12	-	0.73	1.55	0.60	1.89	0.82	1.10	0.59	-	-	-	-	-	-
		Month 24	-	0.75	0.64	0.39	1.00	0.57	1.14	0.33	-	-	-	-	-	-
		**Schedule B**														
		**SCD**														
		Baseline	-	0.02	0.05	0.08	0.11	0.03	0.08	0.04	-	-	-	-	-	-
		Month 13	-	1.67	0.82	2.8	0.32	3.54	1.16	0.98	-	-	-	-	-	-
		Month 24	-	0.64	0.44	0.64	2.31	0.41	2.17	0.58	-	-	-	-	-	-
		Month 25	-	38.05	19.06	32.55	58.10	13.29	90.35	13.59	-	-	-	-	-	-
		**HCs**														
		Baseline	-	0.03	0.13	0.18	0.04	0.03	0.21	0.09	-	-	-	-	-	-
		Month 13	-	2.20	0.45	1.59	2.08	2.62	2.27	0.55	-	-	-	-	-	-
		Month 24	-	0.83	1.0	2.32	2.88	0.90	3.33	2.00	-	-	-	-	-	-
Reinert (2007)	ELISA PS-specific IgG GMCs in ug/mL	**Primary series**														
		Baseline	-	0.08	0.23	0.25	0.49	0.16	0.35	0.13	-	-	-	-	-	-
		Month 5	-	7.81	5.93	5.23	14.76	4.59	8.15	4.55	-	-	-	-	-	-
		Fold rise		*98.63*	*24.42*	*20.59*	*31.24*	*28.13*	*22.80*	*34.04*	-	-	-	-	-	-
	OPA PS-specific OPA GMT	**Booster (N=46)**														
		Baseline	-	0.71	1.60	0.74	3.76	0.47	1.20	0.75	0.02	0.14	0.26	-	-	-
		Month 1	-	29.49	26.66	14.65	21.79	6.32	28.98	12.96	2.82	2.46	2.03	-	-	-

	(assay not described and data not shown)	*Fold rise*<br>*≥0.35 (%)*<br>*≥1.0 (%)*	-<br>-<br>-	*39.81*<br>-<br>-	*16.48*<br>-<br>-	*19.36*<br>-<br>-	*5.68*<br>-<br>-	*13.08*<br>-<br>-	*23.85*<br>-<br>-	*16.69*<br>-<br>-	-<br>*95.3%*<br>*79.1%*	-<br>*100%*<br>*86%*	-<br>*90.7%*<br>*69.8%*	-<br>-<br>-	-<br>-<br>-	-<br>-<br>-
Rivera-Olivero (2014)	ELISA PS-specific GMCs in ug/mL.	Baseline<br>≥1 m last dose	-<br>-	0.53<br>4.63	1.8<br>5.28	0.73<br>4.68	3.14<br>17.16	0.71<br>4.54	2.59<br>6.81	1.03<br>5.93	-<br>-	-<br>-	-<br>-	-<br>-	-<br>-	-<br>-
De Montalembert (2015)	PS-specific IgG GMCs, assay not described.<br><br>OPA assay not described.	**IgG**<br>**1st PCV13**<br>Baseline<br>Month 1	-<br>-	0.95<br>6.81	5.49<br>27.14	3.00<br>9.04	5.58<br>34.74	1.29<br>7.74	4.29<br>19.61	2.92<br>17.80	1.44<br>5.15	1.04<br>2.03	4.33<br>7.05	5.23<br>17.25	2.03<br>9.37	7.86<br>26.73
		**2nd PCV13**<br>(N=146)<br>Month 1<br>Month 12	-<br>-	5.09<br>2.02	24.24<br>11.64	7.46<br>4.49	26.19<br>16.96	5.44<br>2.72	20.56<br>11.29	16.18<br>7.75	4.51<br>2.24	1.67<br>1.03	6.33<br>4.33	16.37<br>8.89	7.06<br>3.34	22.19<br>13.55
		**OPA**<br>**1st PCV13**<br>Baseline<br>Month 1	-<br>-	269<br>2700	888<br>7624	384<br>2639	749<br>2295	601<br>4537	105<br>1610	29<br>1670	6<br>55	13<br>106	11<br>262	286<br>7611	376<br>3224	117<br>1424
		**2nd PCV13**<br>(N=146)<br>Month 1<br>Month 12	-<br>-	2936<br>1091	7705<br>3446	3354<br>1543	2096<br>1741	5462<br>1469	1523<br>537	2202<br>844	77<br>24	105<br>34	280<br>99	7127<br>2296	3692<br>2264	1300<br>630
Disu (2016)	ELISA Pneumococcal-specific IgG levels in ug/mL	**SCD**<br>Baseline<br>Month 2<br>≥4 (%)	420.6<br>2088.6<br>*85%*	-<br>-<br>-	-<br>-<br>-	-<br>-<br>-	-<br>-<br>-	-<br>-<br>-	-<br>-<br>-	-<br>-<br>-	-<br>-<br>-	-<br>-<br>-	-<br>-<br>-	-<br>-<br>-	-<br>-<br>-	-<br>-<br>-
		**HCS**<br>Baseline<br>Month 2<br>≥4 (%)	327.5<br>1996.3<br>*37%*	-<br>-<br>-	-<br>-<br>-	-<br>-<br>-	-<br>-<br>-	-<br>-<br>-	-<br>-<br>-	-<br>-<br>-	-<br>-<br>-	-<br>-<br>-	-<br>-<br>-	-<br>-<br>-	-<br>-<br>-	-<br>-<br>-
Sirima (2017)	ELISA PS-specific IgG	**≤6 SCD**<br>**IgG**	-													

	GMCs in ug/mL	Baseline	-	0.04	0.07	0.12	0.77	0.13	0.34	0.13	0.09	-	0.06	0.10	0.08	0.24
		20 weeks	-	2.25	1.29	4.56	4.30	14.60	11.87	1.32	3.51	-	5.15	0.12	4.91	0.26
		9-10 months	-	1.20	1.94	1.61	2.58	3.82	3.31	0.67	0.67	-	1.13	0.40	1.83	0.23
	OPA GMTs in ug/mL	10-11 months		7.04	5.07	8.19	8.66	16.52	10.96	4.86	5.34	-	6.30	0.48	9.03	1.09
		**OPA**														
		20 weeks	-	1316	1043.3	1438.6	1689.9	873.8	558.9	705.1	106.4	-	106.9	24.7	4644.1	9.4
		9-10 months	-	222.1	285.3	215.5	215.5	46.5	60.7	85.2	11.7	-	14.9	18.2	1747.2	6.3
		10-11 months	-	2064.2	952.2	2062.3	1571.5	1246	652.9	4231.2	930.5	-	273.2	35.3	7262.9	22.1
		**<6 HCs**														
		**IgG**														
		Baseline	-	0.06	0.11	0.12	0.65	0.13	0.30	0.10	0.09	-	0.07	0.15	0.13	0.23
		20 weeks	-	3.52	1.13	4.59	5.95	11.33	9.78	1.41	3.63	-	5.94	0.10	4.28	0.25
		9-10 months	-	1.08	1.62	1.44	2.10	3.16	3.27	0.77	0.69	-	1.23	0.18	1.57	0.21
		10-11 months	-	6.02	5.00	7.95	7.43	16.74	11.27	4.54	5.14	-	6.91	0.36	8.19	0.85
		**OPA**														
		20 weeks	-	992.5	446.2	1116.8	1062.3	524.7	266.1	759.7	94.6	-	119.4	9.4	4924.2	5.0
		9-10 months	-	108.3	245.6	24.5	132.5	28.7	43.4	41.6	10.9	-	15.8	17.6	1585.7	5.8
		10-11 months	-	2079.3	989.2	2987.2	1454.1	1011.8	486.7	1454	750.4	-	277.5	30.7	8120.3	19.4
		**7-11 SCD**														
		**IgG**														
		Baseline	-	0.03	0.03	0.04	0.08	0.03	0.05	0.03	0.03	-	0.04	0.03	0.04	0.04
		9-12 months	-	10.88	1.61	2.55	4.91	12.92	11.13	1.29	5.48	-	6.76	0.18	8.51	0.46
		10-14 months	-	5.72	1.51	1.90	4.71	8.43	6.54	1.00	2.68	-	3.68	0.23	5.46	0.44
		11-15 months	-	9.75	3.12	4.73	10.24	23.57	15.59	3.11	5.51	-	7.75	0.44	11.08	1.49
		**OPA**														
		Baseline	-	5.1	7.9	16.9	5.5	4.1	4.2	7.6	4.9	-	4.4	4.8	215.3	4.5
		9-12 months	-	1636.3	702.5	2858.2	2109.5	744.4	393.4	1545.1	134.7	-	105.7	40.6	6694.3	15.2
		10-14 months	-	812.7	708.3	1982.9	1431.6	411.4	160.2	1168.8	74.2	-	54.1	38.1	7776.9	14.6
		11-15 months	-	2130.5	1360	3047.9	3414.9	2218.9	1347.7	2038.8	516	-	277.4	77.1	10855	76.1
		**7-11 HCs**														
		**IgG**														
		Baseline	-	0.03	0.03	0.04	0.07	0.03	0.04	0.04	0.04	-	0.06	0.03	0.04	0.03
		9-12 months	-	7.55	1.48	1.67	4.81	14.62	9.77	0.93	3.99	-	4.59	0.16	6.67	0.16
		10-14 months	-	4.32	1.35	1.52	4.98	11.49	7.39	1.08	2.44	-	3.40	0.22	4.68	0.22

		11-15 months	-	7.88	2.93	3.76	10.69	31.88	15.85	3.17	4.92	-	7.87	0.43	10.29	0.43
		**OPA**														
		Baseline	-	11.8	8.5	20.3	5.9	4.6	4.0	15.3	4.5	-	4.0	5.0	643.5	4.3
		9-12 months	-	1771.1	696.6	2667.9	4009.9	1605.6	491.5	2107.5	88.1	-	85.8	51.2	10452	108.3
		10-14 months	-	1048.3	615.5	1986.2	2128.3	746.4	279.5	1026.2	48	-	49.2	36.3	9336.3	19.4
		11-15 months	-	2415.5	1305.4	2719.2	3717.3	3238.7	1174.4	3525.1	511.3	-	289.6	98.2	9362.8	449.1
		**12-23 SCD**														
		**IgG**														
		Baseline	-	0.04	0.03	0.08	0.11	0.04	0.06	0.03	0.04	-	0.06	0.03	0.07	0.06
		14 25 months	-	4.22	0.41	0.99	1.87	6.21	5.88	0.50	1.39	-	1.06	0.15	2.74	0.77
		15-26 months	-	8.87	1.37	2.35	7.59	25.52	18.00	1.95	4.67	-	5.52	0.39	6.81	3.15
		**OPA**														
		Baseline	-	8.0	13.6	119.5	16.3	4.9	5.5	68.1	6.9	-	5.5	9.3	1436.6	5.8
		14 25 months	-	1086.3	345.1	1976.2	711.2	1035.5	229.1	2572.2	9.3	-	9.8	82.8	5462.3	28.4
		15-26 months	-	2089.7	748.4	3778.2	2704.5	2873.1	1845.3	5016.6	195.7	-	151.3	147.9	10279	214.2
		**12-23 HCs**														
		**IgG**														
		Baseline	-	0.04	0.04	0.05	0.09	0.05	0.08	0.05	0.04	-	0.08	0.04	0.05	0.07
		14 25 months	-	3.74	0.34	0.83	1.24	6.12	5.12	0.35	1.49	-	1.10	0.12	3.17	0.75
		15-26 months	-	7.02	1.25	1.72	5.75	22.64	14.46	1.40	4.26	-	4.07	0.31	6.36	2.79
		**OPA**														
		Baseline	-	7.3	9.9	81.3	12	4.0	4.0	37.6	5.6	-	4.4	6.3	1228.1	6.3
		14 25 months	-	1539.4	278.4	2359	865.6	449.2	248	2433.6	10.9	-	9.6	77.2	5802.2	25.7
		15-26 months	-	3193.9	866.4	4276.8	2737.5	2126.8	1271.4	5325.4	115.1	-	84	157.4	10131	321.1

Study	Endpoint	Timepoint	Anti-pneu	4	6B	9V	14	18C	19F	23F	1	3	5	6A	7F	19A
Melica (2023)	Luminex	**PCV13+PPV23**														
	PS-specific	Baseline	-	0.41	0.42	0.38	5.51	0.46	1.06	0.52	0.70	0.11	0.28	0.25	0.57	1.25
	IgG GMCs	Week 8	-	1.64	3.56	1.96	18.33	2.60	5.63	6.77	2.30	0.45	1.18	4.20	2.62	7.47
	in ug/mL	Week 24	-	1.16	2.55	1.34	15.23	2.11	3.82	3.80	1.72	0.28	0.96	2.68	1.86	4.81
		Week 96	-	0.70	2.19	1.06	14.45	1.56	3.95	4.42	1.38	0.21	0.80	2.43	1.61	5.15
	OPA PS-specific	**PPV23**														
		Baseline	-	0.23	0.61	0.34	4.49	0.56	0.99	0.59	0.73	0.11	0.24	0.25	0.55	1.56

	GMTs in ug/mL	Week 8	-	0.62	1.48	0.88	9.91	1.56	2.46	1.32	2.49	0.30	0.82	0.41	1.64	2.87
		Week 24	-	0.46	1.17	0.70	7.67	1.26	1.89	0.95	1.86	0.22	0.59	0.34	1.24	2.11
		Week 96	-	0.30	0.89	0.50	6.65	0.96	1.33	0.63	1.25	0.15	0.39	0.25	0.89	1.45
		**OPA**														
		**PCV13+PPV23**														
		Baseline	-	197.03	339.54	382.28	892.84	210.39	157.64	52.28	10.9	8.77	18.72	128.25	529.31	269.57
		Week 8	-	2013.2	4996.8	1992	1916.7	3136	1452.2	1566.9	101.39	96.56	152.17	5101.2	2426	1443.1
		**PPV23**														
		Baseline	-	71.14	339.75	350.11	572.26	312.02	101.17	72.07	12.79	8.69	18.30	57.96	505.64	149.50
		Week 8	-	647	1059.4	837.66	1325.6	1420.3	482.65	266.76	87.19	44.88	70.46	303.59	1461.8	387.63

Study	Endpoint	Timepoint	PS 33F	4	6B	9V	14	18C	19F	23F	1	3	5	6A	7F	19A
Quinn et al (2023)	Pneumococcal electro-chemiluminescence (PnECL) v2.0 assay, PS-specific GMCs in ug/mL. Multiplex opsonophagocytic assay, OPA titers in ug/mL	**Group PCV15**														
		**(N=69)**	0.49													
		Baseline	4.46	0.26	1.01	0.60	1.51	0.56	1.70	0.56	0.35	0.21	0.86	0.43	0.51	2.44
		Day 30	*75.8%*	1.58	38.38	4.46	16.03	6.11	13.88	5.38	2.12	1.09	4.44	23.29	5.81	19.86
		≥4 (%)		*56.1%*	*93.9%*	*62.1%*	*63.1%*	*71.2%*	*65.2%*	*69.8%*	*62.1%*	*53%*	*53%*	*93.9%*	*74.2%*	*57.6%*
		**Group PCV13**														
		**(N=34)**	0.66													
		Baseline	0.97	0.28	0.79	0.58	2.59	0.56	1.73	0.48	0.44	0.21	0.98	0.35	0.47	2.66
		Day 30	*6.7%*	2.90	22.94	5.36	20.53	4.20	12.80	6.88	2.76	1.07	6.56	15.97	4.65	21.65
		≥4 (%)		*72.4%*	*89.7%*	*60%*	*55.2%*	*60%*	*66.7%*	*72.4%*	*60%*	*48.3%*	*58.6%*	*93.1%*	*83.3%*	*63.3%*
		OPA														
		**PCV15**	6273.7													
		Baseline	24013.6	447.4	1688.6	1022.3	910.4	680.6	700.8	1583.2	15.8	39.7	38.5	1381.8	3619	1608.9
		Day 30	*42%*	4670.8	31560	4561.8	6597.6	9684.6	4931.8	17191	484	264.8	1383.9	27306	19412	14068
		≥4 (%)		*65.3%*	*83%*	*43.8%*	*56.0%*	*75.6%*	*56.0%*	*58.3%*	*79.6%*	*62.0%*	*81.6%*	*85.1%*	*58.0%*	*64%*
		**PCV13**	6474.2													
		Baseline	4824.8	271.1	1176.4	681.5	1350.3	1008.5	567.2	842.3	30.8	56.4	66.4	1506.2	3335.8	1439.5
		Day 30	*0%*	7015.5	18531	3941.7	8112.2	5685.1	3313.3	19197	504	234.3	1198.2	20277	16928	9224.9
		≥4 (%)		*81%*	*84.2%*	*55%*	*38.1%*	*40.0%*	*55%*	*85%*	*70%*	*42.9%*	*76.2%*	*66.7%*	*52.4%*	*66.7%*
